# Synthesis of rigid *p*-terphenyl-linked carbohydrate mimetics

**DOI:** 10.3762/bjoc.10.182

**Published:** 2014-07-30

**Authors:** Maja Kandziora, Hans-Ulrich Reissig

**Affiliations:** 1 Freie Universität Berlin, Institut für Chemie und Biochemie, Takustraße 3, D-14195 Berlin, Germany

**Keywords:** carbohydrate mimetics, hydrogenolysis, multivalent glycosystems, 1,2-oxazines, samarium diiodide, Suzuki cross-coupling

## Abstract

An approach to β-D-2-aminotalose- and β-D-2-aminoidose-configured carbohydrate mimetics bearing a phenyl substituent is described. Unnatural divalent rigid *p*-terphenyl-linked *C*-aryl glycosides with 2.0 nm dimension are available using Suzuki cross-couplings. The key compound, a *p*-bromophenyl-substituted 1,2-oxazine, was prepared by a stereoselective [3 + 3]-cyclization of a D-isoascorbic acid-derived (*Z*)-nitrone and lithiated TMSE-allene. The Lewis acid-induced rearrangement of this heterocycle provided the corresponding bicyclic 1,2-oxazine derivative that may be regarded as internally protected amino sugar analogue. After subsequent reduction of the carbonyl group, the resulting bicyclic compound was used for Suzuki cross-couplings to form biphenyl aminopyran or *p*-terphenyl-linked dimers. Hydrogenolysis afforded new unnatural aminosugar mimetics. Zinc in the presence of acid or samarium diiodide were examined for the N–O bond cleavage in order to obtain the rigid *p*-terphenyl-linked *C*-glycosyl dimers.

## Introduction

Carbohydrates are the class of biomolecules with the highest structural diversity [1–2]. Specific carbohydrates are responsible for cell-type specific interactions [3] and they are involved in different diseases such as cancer [4], inflammation [5], and infections [6]. However, the use of carbohydrates as drugs has been strongly limited due to the hydrolytic lability of the glycosidic bond [7] and the weak binding affinities of single molecules. With the development of artificial *C*-glycosides which possess structural and functional aspects of the corresponding carbohydrates, these disadvantages can be overcome, resulting in an improved bioavailability, higher affinities and improved selectivities [8–13]. Recent results indicate that divalent rigid carbohydrate conjugates may have even higher binding affinities and specificities than their flexible multivalent equivalents [14–15]. The rigidity of the system is supposed to improve the overall activity of the ligands by overcoming the entropic penalty of flexible multivalent scaffolds [16].

**Scheme 1:**
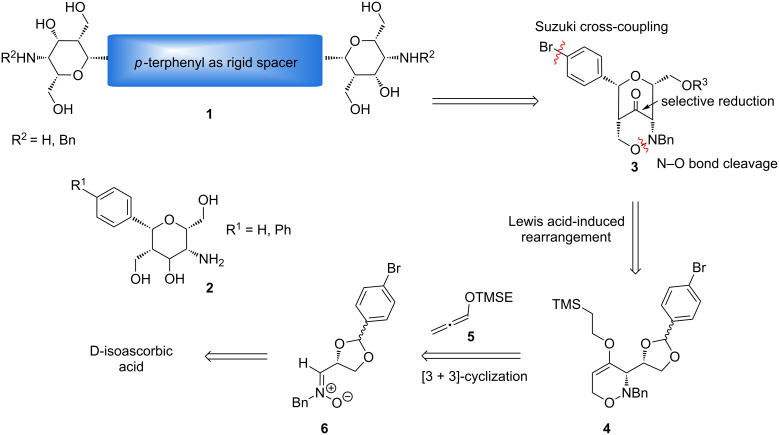
Approach to divalent carbohydrate mimetics **1** with rigid spacer and monovalent analogues **2**.

Cross-coupling reactions are among the best methods to prepare *C*-arylglycosides, *C*-nucleosides and *C*-glycosidic oligomers when new artificial pharmacophores are approached [17]. With Suzuki cross-couplings *C*-glycoside analogues of phloriain with antidiabetic properties [18] or aryl-scaffolded dimers and trimers were successfully prepared [19]. The Suzuki cross-coupling is particularly suitable for carbohydrate chemistry due to the mild reaction conditions and its tolerance to a variety of functional groups [20]. In addition, the reactions are easy to perform and the required boronic acids exhibit exceptional stabilities to heat, air and moisture compared to other organometallic reagents [21]. Up to now there are not many examples of nanorod-like carbohydrate dimers with aryl-linked divalent glycosides. A mannopyranoside dimer was generated by a palladium-catalyzed Ullmann-type reductive homocoupling [22] and biphenyl-linked dimers were prepared by Lewis acid-catalyzed glycosidations [23]. All these examples possess an acid-labile *O*-glycosidic bond and are labile to hydrolysis and enzymes. Therefore, new approaches to the synthesis of rigid multivalent *C*-arylglycosides should be a valuable extension of compounds with potential biological activity. In order to achieve this goal we investigated the synthesis of divalent compounds of general structure **1** and their monovalent analogues **2** (Scheme 1). Structurally similar aminopyrans without aryl groups have intensively been studied as carbohydrate mimetics in our group [24–25]. When they are coupled by amide bonds to gold nanoparticle and O-sulfated these conjugates gave extremely high binding affinities towards L- and P-selectin in sub-nanomolar concentrations. These results were achieved by a multivalent presentation (ca. 1000–1200 ligands per nanoparticle) of the sulfated pyrans [26–27]. We were therefore interested to prepare inhibitors offering only a small number of ligands to get better information about structure–activity relationships and to study the influence of the flexible and rigid spacer units.

In this report we present methods for the synthesis of divalent compounds **1** with *p*-terphenyl spacers and of β-D-2-aminotalose- or β-D-2-aminoidose-configured carbohydrate mimetics **2** (Scheme 1). These novel carbohydrate mimetics represent unique structures, combining the features of *C*-aryl-glycosides and aminosugars. The *p*-bromophenyl-substituted bicyclic 1,2-oxazine derivative **3** was used as key building block for the Suzuki cross-coupling reaction to synthesize *p*-terphenyl-linked derivatives **1**. The key intermediate **3** was prepared by a Lewis acid-induced rearrangement of 3,6-dihydro-2*H*-oxazine **4**, that origins from a stereoselective [3 + 3]-cyclization of D-isoascorbic acid-derived (*Z*)-nitrone **6** and lithiated TMSE-allene **5**.

## Results and Discussion

**Scheme 2:**
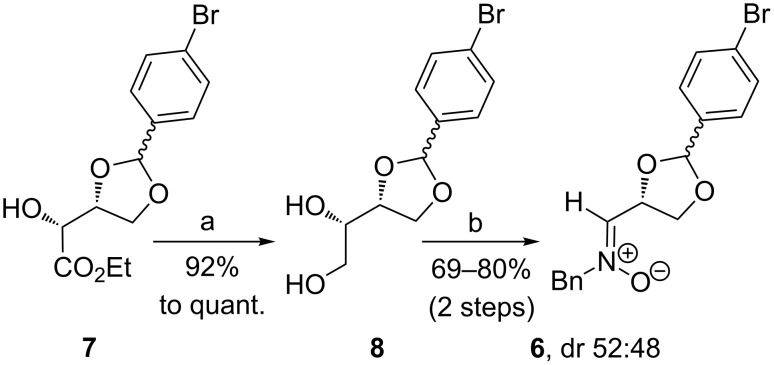
Synthesis of (*Z)*-nitrone **6**. Conditions: a) LiAlH_4_, THF, 1 h, rt; b) 1. NaIO_4_, CH_3_CN/H_2_O, 1 h, rt; 2. *N*-benzylhydroxylamine, MgSO_4_, CH_2_Cl_2_, 18 h, rt.

For our synthesis of new divalent carbohydrate mimetics we required 1,2-oxazine derivatives derived from (*Z*)-nitrone **6** and lithiated alkoxyallenes. The 4-bromophenyl group should allow transition metal-promoted coupling reactions to a variety of new compounds. For this purpose the D-erythrose-configured ester **7**, easily available from D-isoascorbic acid [28], was converted into nitrone **6** in a three step procedure (Scheme 2). Its reduction with lithium aluminum hydride was performed under standard conditions providing diol **8** in excellent yield in multigram scale (up to 20 g). Attention should be paid to a possible reductive removal of the bromine substituent that can occur at higher temperature or longer reaction times as the resulting debrominated product is hard to remove from diol **8** by column chromatography. According to the protocol of Dondoni et al. [29] glycol cleavage of diol **8** afforded the corresponding aldehyde that was directly treated with *N*-benzylhydroxylamine to furnish the desired (*Z*)-nitrone **6**. All compounds in this sequence of reactions are mixtures of the two diastereomers at the dioxolane C-2 (ratios close to 1:1).

**Scheme 3:**
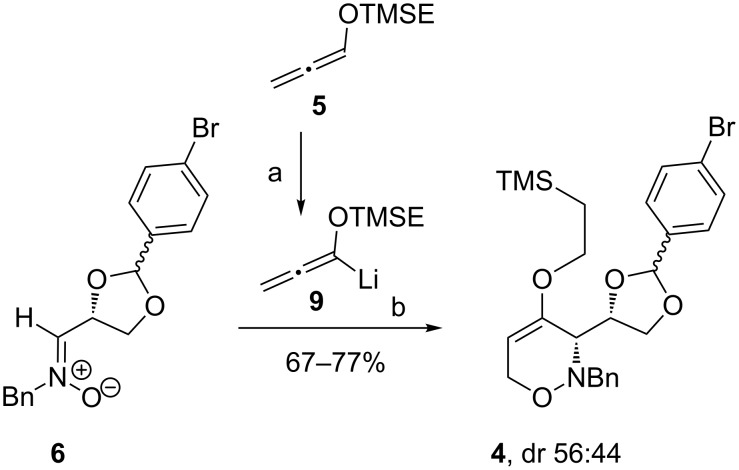
[3 + 3]-Cyclization of (*Z)*-nitrone **6** with lithiated allene **9**. Conditions: a) *n-*BuLi, THF, 15 min, −40 °C; b) 1. THF, 2 h, −78 °C; 2. H_2_O, 1 h, −78 °C → rt.

The preparation of *syn*-1,2-oxazine **4** was achieved in good yields ranging from 67–77% by stereocontrolled addition of lithiated (trimethylsilyl)ethoxyallene **9** to (*Z*)-nitrone **6** at −78 °C (Scheme 3). Although the formation of four stereoisomers is possible only two were observed. Due to the complexation of lithiated allene **9** to the nitrone **6** an exclusive formation of the two *syn*-1,2-oxazines **4** was observed. This result suggests that the configuration at C-2 of the dioxolane moiety has no influence on the stereochemical outcome of the reaction. The model suggested by Dondoni et al. [30] can also be employed for this process to rationalize the observed diastereoselectivity of the addition step. The subsequent [3 + 3]-cyclization to 3,6-dihydro-2*H*-1,2-oxazine **4** follows the previously reported mechanism [31]. In the presented sequence *syn*-1,2-oxazine **4** was successfully prepared in six steps with an overall yield of 46%. The diastereomers can easily be separated by column chromatography, but this turned out not to be mandatory. The next step of our anticipated sequence, the Lewis acid-induced rearrangement, converts the dioxolane C-2 carbon into an sp^2^-hybridized carbon and hence the configuration of the precursor does not play a role for this reaction.

**Scheme 4:**
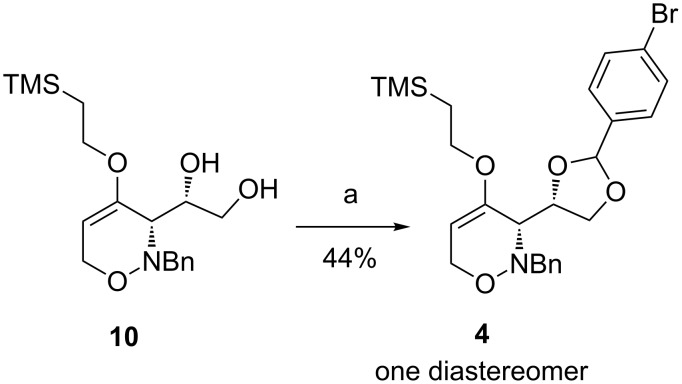
Synthesis of 1,2-oxazine **4** by acetal formation from **10**. Conditions: a) 1-bromo-4-(dimethoxymethyl)benzene (10 equiv.), CAN, CH_2_Cl_2_, 3 d, rt.

An alternative route to prepare 1,2-oxazine **4** is depicted in Scheme 4. The preparation of the 4-bromophenyl-1,3-dioxolane moiety started from diol **10** that has successfully been used earlier for the preparation of phenylthio-substituted 1,2-oxazine derivatives [32]. Compound **10** is easily accessible by a mild cleavage of the corresponding acetonide by an indium trichloride-mediated hydrolysis [33]. By using cerium ammonium nitrate as Lewis acid [34] in high concentration (13 mmol/mL) as well as an excess of 1-bromo-4-(dimethoxymethyl)benzene enabled the synthesis of *syn*-1,2-oxazine **4**. The conversion of this reaction was high (>80%) giving the two diastereomers of **4** (ca. 1:1), but only one diastereomer was isolated in pure form. The second diastereomer could hardly be separated from the excess of 1-bromo-4-(dimethoxymethyl)benzene by column chromatography or distillation. Besides, Brønsted acids like trifluoroacetic acid, *p-*toluenesulfonic acid, that are usually used to generate ketals, or weaker acids like pyridine/hydrogen fluoride led to a side product [35].

**Scheme 5:**
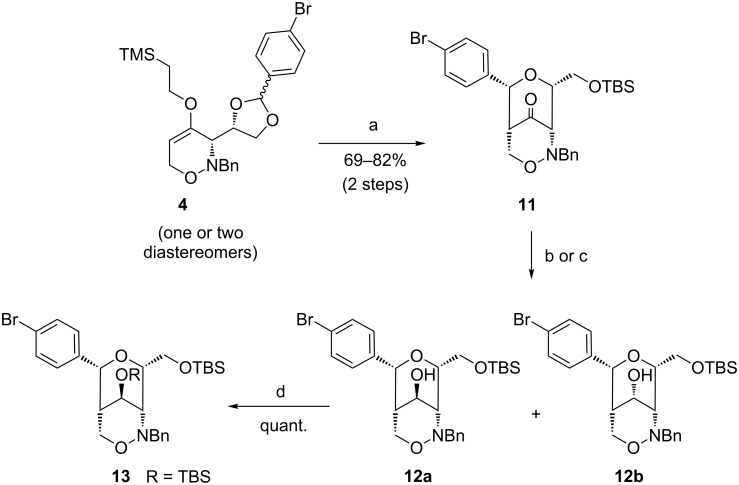
Synthesis of bicyclic ketone **11** by Lewis acid-induced rearrangement and reduction to alcohols **12a** and **12b** and protection of **12a** to 1,2-oxazine derivative **13**. Conditions: a) 1. SnCl_4_, CH_3_CN, 4 h, −30 °C → rt; 2. TBSCl, imidazole, THF, 4 h, rt; b) NaBH_4_, EtOH, 4 h, −40 °C, 72%, dr 81:19; c) L-selectride, THF, 2 h, −10 °C, 73%, only **12a**; d) TBSOTf, 2,6-lutidine, THF, 2 h, 0 °C.

The Lewis acid-promoted rearrangement of 1,3-dioxolanyl-substituted 1,2-oxazines to bicyclic ketones has been described in many examples [24]. Gratifyingly, starting from 1,2-oxazine **4** with tin(IV) chloride as Lewis acidic promoter the corresponding ketone was obtained in excellent stereoselectivity. The subsequent protection of the primary hydroxy group as TBS ether under standard conditions provided **11** in very good yields of up to 82% (Scheme 5). In order to perform the Lewis acid-promoted rearrangement and the protection in one step, we also employed TBSOTf as Lewis acid for the rearrangement step [24], however, no product formation could be observed in this case. The mechanism of the rearrangement **4** → **11** can be described as an aldol-type cyclization process. The Lewis acid coordinates to the sterically less hindered oxygen atom of the dioxolane ring of **4** opening this ring and forming a carbenium ion that intramolecularly attacks the enol ether moiety. A six-membered chair-like transition state with the bulky 4-bromophenyl group in an equatorial position for this crucial step rationalizes the product configuration as shown.

TBS-protected bicyclic ketone **11** was subsequently reduced with sodium borohydride at −40 °C to form alcohols **12a** and **12b** as 81:19 mixture of diastereomers in 72% yield (Scheme 5). In contrast, the reduction with L-selectride at −10 °C selectively furnished pure diastereomer **12a** in 73% yield. In accordance with previous observations of reductions of related phenylthio-substituted bicyclic compounds [25], a hydride attack from the side of the pyran moiety is assumed since the 1,2-oxazine side is more hindered by the bulky *N*-benzyl moiety. The secondary hydroxy group of **12a** was protected employing *t*-butyldimethylsilyl trifluoromethanesulfonate and 2,6-lutidine in quantitative yield.

**Scheme 6:**
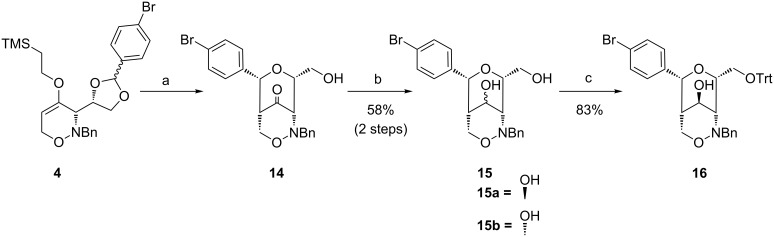
Synthesis of bicyclic diols **15** and of trityl-protected bicyclic 1,2-oxazine **16**. Conditions: a) SnCl_4_, CH_3_CN, 18 h, −30 °C → rt, b) 1) L-selectride, THF, 4 h, −15 °C, dr 72:28; 2) separation by column chromatography (silica gel, hexanes/EtOAc 1:1 → 1:2); c) TrtCl, DMAP, pyridine, 3 d, rt.

The Lewis acid-promoted rearrangement of 1,2-oxazine derivative **4** and the direct reduction of the unpurified ketone **14** furnished bicyclic diol **15** in good overall yield of 58% (Scheme 6). The reduction of unprotected ketone **14** with L-selectride was less stereoselective and provided a 72:28 mixture of **15**. The higher selectivity of the TBS-protected compound **11** may be explained by an indirect effect of the bulky TBS-group, possibly pushing the *N*-benzyl moiety to the top of the ring shielding the 1,2-oxazine side more efficiently. Separation of the two diastereomers and protection of the primary hydroxy group of **15a** with trityl chloride provided compound **16** in 83% yield. This protecting group should allow its removal together with the benzyl group during hydrogenolysis. Surprisingly, a benzyl protection under the same conditions was not possible.

**Scheme 7:**
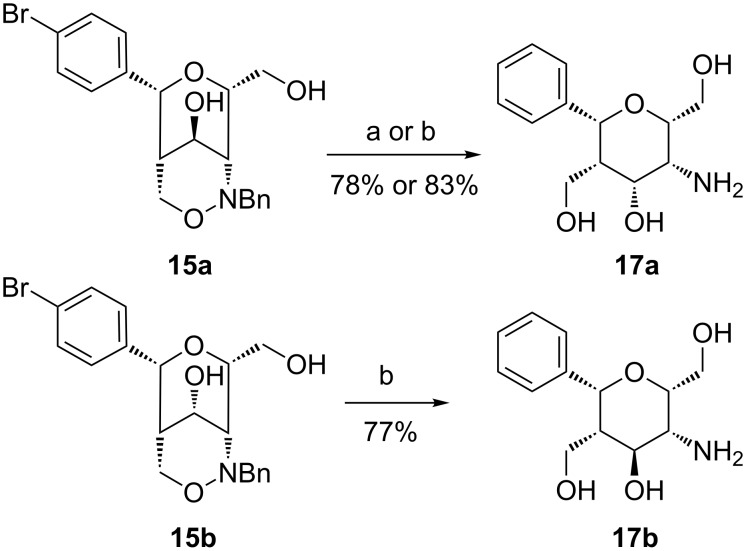
Hydrogenolyses of bicyclic 1,2-oxazine derivatives **15a** and **15b**. Conditions: a) H_2_, Pd/C, MeOH, EtOAc, 20 h, rt; b) H_2_, Pd/C, iPrOH, EtOAc, NEt_3_, 18 h, rt.

Before approaching divalent compounds such as **1** we wanted to convert our building blocks into simple monocyclic carbohydrate mimetics. To prepare phenyl-substituted aminopyrans the N–O bond of bicyclic compounds **15a** and **15b** was cleaved by hydrogenolysis. These reactions are challenging because the resulting aminopyrans are apparently poisoning the catalyst and hence large amounts of palladium on charcoal are required for full conversion. We did not add acid to diminish catalyst poisoning since we were afraid of other side reactions of the complex product. In addition, the resulting products are very polar and difficult to purify. In our recent report [28] related compounds were reduced in methanol as solvent providing several side products and the yield of the reactions were not fully reproducible. Nevertheless, 1,2-oxazine **15a** was converted into aminopyran **17a** (Scheme 7) by hydrogenolysis under standard conditions in methanol in a yield of 78%, but this yield was not fully reproducible and the conditions were optimized. We found that isopropanol as solvent and addition of one equivalent triethylamine were more reliable and the yield of **17a** could be slightly improved. Triethylamine was added to neutralize the formed acid [36] that is generated in the first step by a very fast debromination. The debenzylation and the N–O bond cleavage occur as next steps. Under these improved conditions the isomeric bicyclic 1,2-oxazine **15b** was converted into aminopyran **17b** in a good yield of 77%. The formed aminopyrans **17a** and **17b** can be regarded as amino *C*-glycosides. Compound **17a** is related to compounds with β-D-talose configuration that are rarely found in nature, an exception being the antibiotic amino glycoside hygromycin B [37]. Aminopyran **17b** correlates to β-D-idopyranose; iduronic acid is a component of sulfated glycosamine glycans such as chrondroitin sulfate and heparan sulfate [38].

**Scheme 8:**
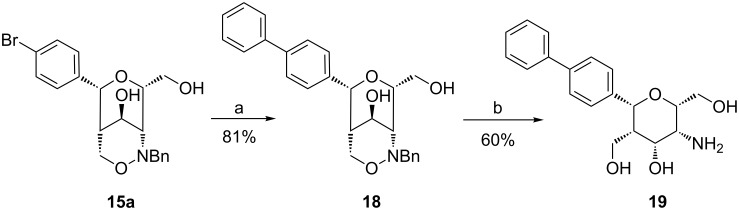
Suzuki cross-coupling of **15a** leading to biphenyl derivative **18** and hydrogenolysis to **19**. Conditions: a) phenylboronic acid, Pd(PPh_3_)_4_, 2 M Na_2_CO_3_ aq, THF, 70 °C, 48 h; b) H_2_, Pd/C, iPrOH, THF, rt, 24 h.

The prepared *p*-bromophenyl-substituted bicyclic 1,2-oxazine derivatives **12**, **13**, **15** and **16** provide options to perform cross-coupling reactions such as Buchwald/Hartwig, Heck, Hiyama, Kumada, Sonogashira or Stille couplings. In order to examine the conditions for Suzuki cross-couplings we subjected bicyclic compound **15a** to phenylboronic acid under standard conditions of this reaction. The desired product **18** was obtained in 81% yield (Scheme 8) and the subsequent hydrogenolysis furnished carbohydrate mimetic **19** bearing a biphenyl substituent.

**Scheme 9:**
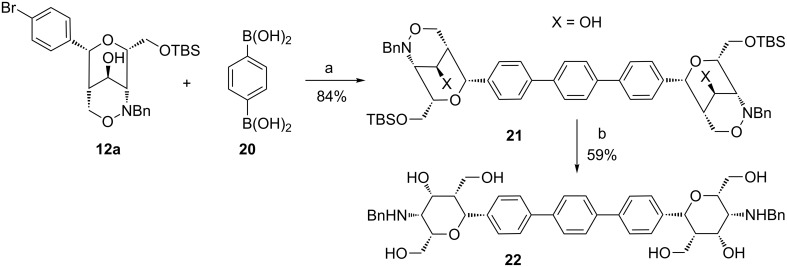
Synthesis of *N*-benzylated *p*-terphenyl derivative **21** by Suzuki cross-coupling of **12a** with **20** and subsequent reduction with zinc. Conditions: a) Pd(PPh_3_)_4_, 2 M Na_2_CO_3_ aq, THF/DMF (8:2), 70 °C, 48 h; b) Zn, AcOH, THF, 60 °C, 18 h.

The smooth transformation of bicyclic compound **15a** into a biphenyl compound by Suzuki cross-coupling encouraged us to aim the synthesis of divalent compound **21**. 1,4-Phenylenediboronic acid (**20**) was used as precursor and coupled to two equivalents of **12a** to afford the desired *p*-terphenyl compound **21** in excellent 84% yield (Scheme 9). Unfortunately, the subsequent hydrogenolysis of this compound under conditions as above led to a complex product mixture and no product could be observed. An alternative method for N–O cleavage employs elemental zinc in the presence of acid [39], conditions that should simultaneously cleave the TBS protective groups. For the conversion of **21** into **22** long reaction times were required and the high acidity led to the formation of side products. Nevertheless, the target compound **22** was isolated in a moderate yield of 59%.

**Scheme 10:**
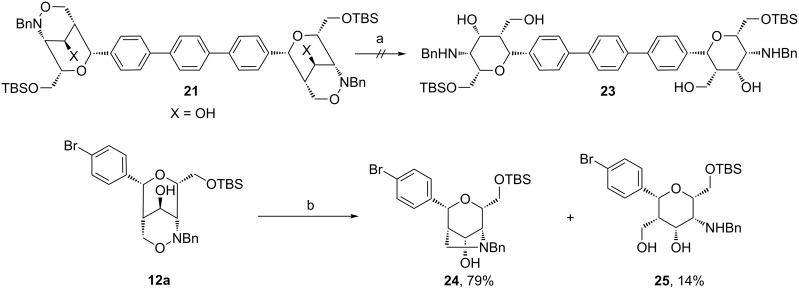
Attempted reductive cleavage of the N–O bond of compound **21** by samarium diiodide and reaction of **12a**. Conditions: a) SmI_2_ (0.09 M in THF), MeOH, rt, 60 min; b) SmI_2_ (0.1 M in THF), THF, rt, 45 min.

As a milder alternative, samarium diiodide was examined for the N–O bond cleavage [40–45]. With this selective reagent a TBS protected aminopyran dimer **23** was expected that should be well soluble in organic solvents and therefore more suitable for subsequent transformations (Scheme 10). Disappointingly, a samarium diiodide solution converted compound **21** in a complex reaction mixture. For a better understanding of this unexpected result the reductive cleavage was examined with the simpler bicyclic 1,2-oxazine derivative **12a**. Here the unexpected bicyclic compound **24** was isolated as major product in 79% yield together with the desired aminopyran derivative **25** in 14% yield. It was observed by ^1^H NMR spectroscopy that aminopyran **25** is not stable and slowly cyclizes to **24**; after two days in CDCl_3_ solution approximately 20% of aminopyran **25** were converted into **24**. This result indicates that the N–O bond cleavage of **12a** preceded as expected, but that the produced amino group of the pyran ring seems to be in close proximity to the C-5 hydroxy group and leads to a nucleophilic substitution under formation of the pyrrolidine moiety. This process is possibly promoted by samarium(III) which can act as Lewis acid and by the steric demanding TBS group which decreases the distance between the two functional groups. This unexpected side reaction leading to **24** indicates that in the above mentioned unclean reaction of samarium diiodide with dimer **21** similar complications may lead to the observed mixture.

**Scheme 11:**
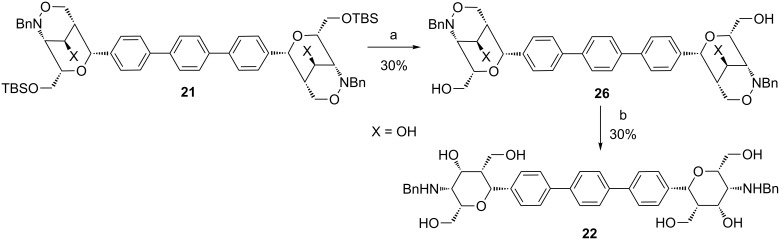
Deprotection of compound **21** and samarium diiodide-mediated reaction of **26**. Conditions: a) TBAF, THF, rt, 2 d; b) SmI_2_ (0.09 M in THF), MeOH, rt, 60 min.

To overcome these difficulties dimer **21** was deprotected with tetra-*n*-butylammonium fluoride giving the poorly soluble polyhydroxylated compound **26** (Scheme 11). Due to the amphiphilic character this compound was only soluble in pyridine that makes purification and subsequent reactions fairly difficult. The conversion of the deprotection step was high but the yield after purification was only 30%. The reduction with samarium diiodide was then performed in a methanol/tetrahydrofuran mixture in which compound **26** was scarcely soluble. Gratifyingly, after one hour reaction time and purification by column chromatography aminopyran **22** was isolated in 30% yield. Interestingly, no cyclization product similar to **24** was detected in this case.

**Scheme 12:**
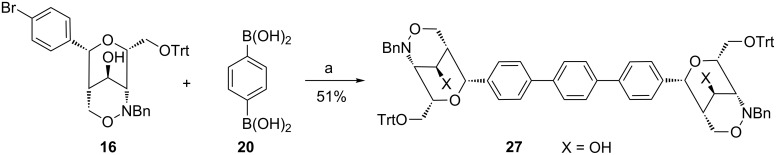
Suzuki cross-coupling of compound **16**. Conditions: Pd(PPh_3_)_2_Cl_2_, 2 M Na_2_CO_3_, DMF, 80 °C, 3 d.

We also investigated the Suzuki cross-coupling of bicyclic compound **16** with 1,4-phenylenediboronic acid (**20**) (Scheme 12). Although the trityl group is quite far away from the reacting bromo substituent it led to longer reaction times and slightly lower yields, but the expected product **27** was obtained in 51% yield as a well soluble compound.

**Scheme 13:**
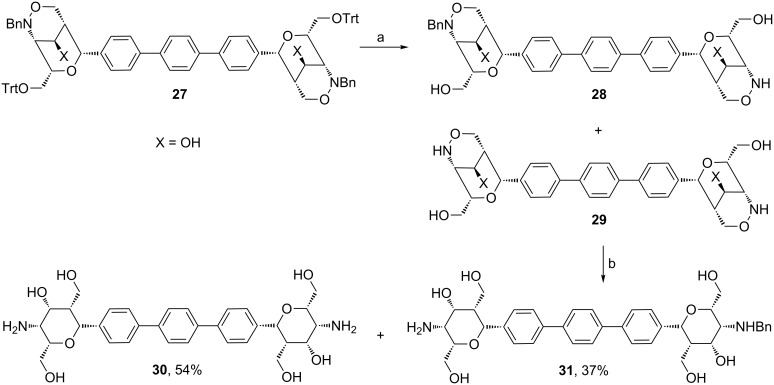
Hydrogenolysis of compound **27** and samarium diiodide-mediated reaction leading to compounds **30** and **31**. Conditions: a) H_2_, Pd/C, TFA, HFIP, iPrOH, 8 h, rt; b) SmI_2_ (0.1 M in THF), MeOH, 30 min, rt. HFIP = hexafluoro-2-propanol.

The trityl protective groups enabled the deprotection of dimer **27** in one step. The *O*-trityl and *N*-benzyl groups were removed by hydrogenolysis under acidic conditions to obtain a mixture of compounds **28** and **29** (Scheme 13). As solvent a 3:2 mixture of isopropanol/hexafluoro-2-propanol was used in order to combine high polarity with product solubility and to avoid side product formation [26]. The ratio of mono-benzylated **28** and fully deprotected *p*-terphenyl derivative **29** was 59:41 as confirmed by ^1^H NMR spectroscopy of the crude product. The reaction mixture was directly filtered through a pad of Celite^®^ and the solvents were removed in vacuo to provide a crude product that was used for the subsequent samarium diiodide-mediated reduction without any purification. This reaction proceeded smoothly and furnished the very polar compounds **30** and **31**. Removal of the formed samarium salts by size exclusion chromatography provided the two divalent carbohydrate mimetics in very good overall yield. The considerably better yield in this samarium diiodide-mediated reaction is probably due to the good solubility of compounds **28** and **29** in methanol/THF.

In summary, by optimizing the protective group strategy and the reductive cleavage methods we were able to prepare the desired rigid *p*-terphenyl-linked carbohydrate mimetic **30** in twelve steps starting from D-isoascorbic acid, but in only six steps with respect to crucial intermediate **4**. The overall yields of 6% or 13% are quite respectable. The distance between the two terminal amino groups is in the range of 2.0 nm (according to optimized molecular geometry obtained by MM2 calculations performed by ChemBio3D Ultra 11.0 from ChemBioOffice 2008).

## Conclusion

We successfully established methods for the efficient preparation of phenyl-substituted aminopyrans and rigid divalent *p*-terphenyl-linked *C*-aryl glycoside using Suzuki cross-couplings as key method. Starting from the D-isoascorbic acid-derived diol **8**, which was converted into the corresponding *p*-bromophenyl-substituted (*Z*)-nitrone, a stereoselective [3 + 3]-cyclization with lithiated TMSE-allene provided the required 1,2-oxazine **4** in six steps with an overall yield of 46%. Alternatively, this 1,2-oxazine could be obtained under the formation of a 4-bromophenyl-1’,3’-dioxolane moiety from diol **10** (actually derived from D-mannitol) which is a promising route to differently substituted 1,2-oxazines, introducing the dioxolane substituent at a late stage. The Lewis acid-induced rearrangement of the 1,2-oxazine **4** afforded the bicyclic ketone **11** which can be regarded as an internally protected amino sugar. After subsequent reduction of the carbonyl group the resulting bicyclic compound **12** was used as substrate for Suzuki cross-couplings to form a biphenyl-substituted aminopyran or rigid *C*-aryl dimers in good yields. These *p*-terphenyl-linked aminopyran derivatives **22**, **30** and **31** have a dimension of approximately 2.0 nm.

*p*-Bromophenyl-substituted intermediates such as **15** or **16** are also useful precursors for the synthesis of other *C*-aryl glycosides with potential biological activity. By simple hydrogenolysis of the bicyclic compounds, three new aminopyrans could be synthesized. Different methods for N–O bond cleavage like palladium-catalyzed hydrogenolysis, zinc/acid or samarium diiodide were tested. For each of the substrates the suitable method has to be found. By the N–O bond cleavage of compound **12a** with samarium diiodide an unexpected bicyclic pyrrolidine derivative **24** was isolated. The prepared unnatural *C*-aryl glycosides could find applications in medicinal chemistry, e.g., as selectin inhibitors. After O-sulfation the biological activity of these divalent compounds will be studied together with that of related carbohydrate mimetics.

## Experimental

For general methods: See Supporting Information File 1

## Supporting Information

File 1 Experimental procedures.

File 2 Characterization data ^1^H NMR and ^13^C NMR spectra of synthesized compounds.
