# Supplementary material for: Synthesis of rigid p-terphenyl-linked carbohydrate mimetics
Source: Beilstein J Org Chem. 2014 Jul 30;10:1749–58. doi: 10.3762/bjoc.10.182 (PMC4142845; doi:10.3762/bjoc.10.182)
Supplement: File 1 — Experimental procedures. [file Beilstein_J_Org_Chem-10-1749-s001.pdf]

# Supporting Information File 1

# for

# Synthesis of rigid *p*-terphenyl-linked carbohydrate mimetics

Maja Kandziora and Hans-Ulrich Reissig*

Address: Freie Universität Berlin, Institut für Chemie und Biochemie, Takustraße 3, D-14195 Berlin, Germany

Email: Hans-Ulrich Reissig - hans.reissig@chemie.fu-berlin.de

* Corresponding author

## Experimental procedures

**Table of contents:**

- General information s2

- Additional experimental procedures and analytical data s4

- References s32

**General information**

Reactions were generally performed under inert atmosphere (argon) in flame-dried flasks. Solvents and reagents were added by syringe. Solvents were dried using standard procedures and were purified with a MB SPS-800-dry solvent system. Triethylamine was distilled from $CaH_2$ and stored over KOH under argon atmosphere. Commercial available reagents were used as received without further purification unless otherwise stated. Products were purified by flash chromatography on silica gel (230–400 mesh, Merck or Fluka) or by size exclusion chromatography (Sephadex™ LH-20, GE Healthcare). Unless otherwise stated, yields refer to analytical pure samples. Hydrogenolyses were performed with hydrogen from Air Liquide (Alphagaz 2). TLC-analyses were performed on silica gel coated aluminium plates purchased from Merck. Products were detected by UV-activity and by using staining reagents (Cer/molybdenum reagent, $KMnO_4$ and ninhydrine). NMR spectra were recorded on BRUKER (AV 500, AV 700) and JEOL (ECP 500) instruments. Chemical shifts (δ) are listed in parts per million (ppm) and are reported relative to solvent residual signals: $CDCl_3$ ($^1$H: δ = 7.26 ppm, $^{13}$C: δ = 77.16 ppm), $CD_3OD$ ($^1$H: δ = 3.31 ppm, $^{13}$C: δ = 49.00 ppm) or pyridine-$d^5$ ($^1$H: δ = 8.74 ppm, $^{13}$C: δ = 150.35 ppm). Integrals are in accordance with assignments; coupling constants (*J*) are given in Hz. All $^{13}$C NMR spectra are proton–decoupled. Multiplicity is indicated as follows: s (singlet), $s_{br}$ (broad singlet), d (doublet), t (triplet), q (quartet), dd (doublet of doublet), dt (doublet of triplet), td (triplet of doublet), m (multiplet), $m_c$ (centered multiplet). For detailed peak assignments 2D spectra were measured (COSY and HMQC). The given ratios of diastereomers were calculated by comparison of the 2’-H peaks. IR spectra were measured with a Jasco spectrometer (FT/IR-4100 with DLATGS Detector). HRMS analyses were performed with Agilent 6210

(ESI–TOF, 10 µL/min, 1.0 bar, 4 kV) and Varian/Agilent Ionspec QFT-7 (ESI–FTICR, 4 µL/min, 1.0 bar, 4 kV) instruments. Elemental analyses were carried out with instruments from PerkinElmer (CHN-Analyzer 2400) and from Elementar (Vario, Vario EL, Vario EL III). Melting points were measured with a Reichert apparatus (Thermovar) and are uncorrected.

**Additional experimental procedures and analytical data**

The following compounds were prepared analogously to literature procedures: ester **1** [1], TMSE-allene **5** [2], *N*-benzylhydroxylamine [3], samarium(II) iodide [4] and 1,2-oxazine **10** [5].

**(*S*)-1-[(*R*)-2-(4´-Bromophenyl)-1´,3´-dioxolan-4-yl]ethane-1,2-diol (8)**

Under an argon atmosphere, lithium aluminum hydride (3.21 g, 84.6 mmol) was suspended in dry THF (525 mL) at 0 °C. Ester **7** (21.6 g, 65.1 mmol), dissolved in dry THF (220 mL), was dropwise added to the lithium aluminum hydride suspension. After 1 h stirring at rt, the solution was cooled to 0 °C and slowly quenched with water (30 mL). Then, 20% aq. NaOH solution (22 mL) and water (37 mL) were added and the suspension was stirred for further 4 h at rt. The suspension was filtered through a pad of Celite®. The filtrate was extracted with diethyl ether (3 x 500 mL) and the combined organic layers were dried with $Na_2SO_4$, filtered and the solvent was removed in vacuo. The crude product was purified by column chromatography (silica gel, hexanes/EtOAc 1:3) to yield **8** (18.8 g, quant.) as a colorless solid.

The obtained two diastereomers (d.r. 52:48) were not separated.

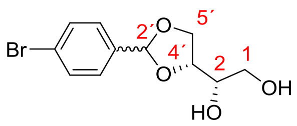


mp 81-83 °C; $[\alpha]_D^{22}$ -0.1 (*c* 1.00, $CH_3OH$); signals with * refer to the major diastereomer: $^1H$ NMR (500 MHz, $CDCl_3$): δ 2.61 ($s_{br}$, 2 H, OH, OH*), 3.00, 3.06 (2 $s_{br}$, 2 H, OH, OH*), 3.62-3.67 (m, 2 H, 1-H, 1-H*), 3.74-3.86 (m, 4 H, 1-H, 1-H*, 2-H, 2-H*), 4.00 (dd, *J* = 6.6,

8.3 Hz, 1 H, 4´-H*), 4.07-4.18 (m, 4 H, 5´-H, 5´-H*), 4.20 (dd, *J* = 6.4, 8.4 Hz, 1 H, 4´-H), 5.72 (s, 1 H, 2´-H), 5.88 (s, 1 H, 2´-H*), 7.30-7.34 (m, 4 H, Ar, Ar*), 7.49-7.52 (m, 4 H, Ar, Ar*) ppm; $^{13}$C NMR (125 MHz, $CDCl_3$): δ 63.5, 63.6 (2 t, C-1, C-1*), 67.4, 67.5 (2 d, C-4´, C-4`*), 72.0, 72.2 (2 d, C-2, C-2*), 76.1, 76.7 (2 t, C-5´, C-5´*), 103.1, 103.3 (2 d, C-2´, C-2´*), 123.4, 123.6 (2 s, Ar, Ar*), 128.0, 128.2, 131.5, 131.6 (4 d, Ar, Ar*), 135.9, 136.8 (2 s, Ar, Ar*) ppm; IR (ATR) $\tilde{\nu}$: 3410-3035 (O-H), 3090-3030 (=C-H), 2955-2870 (C-H), 1580 (Ar), 1250 (C-O) $cm^{-1}$; ESI-TOF (*m*/*z*): $[M + Na]^+$ calcd. for $C_{11}H_{13}BrO_4Na$, 310.9898; found, 310.9895; $[2M + Na]^+$ calcd. for $C_{22}H_{26}Br_2O_8Na$, 600.9881; found, 600.9872; anal. calcd for $C_{11}H_{13}BrO_4$ (289.1): C, 45.70; H, 4.53; found: C, 45.53; H, 4.50.

**(*Z*)-*N*-{[(2*S*,4*S*)-2-(4-Bromophenyl)-1,3-dioxolan-4-yl]methylene}-1-phenylmethanamine oxide (6a) and (*Z*)-*N*-{[(2*R*,4*S*)-2-(4-bromophenyl)-1,3-dioxolan-4-yl]methylene}-1-phenylmethanamine oxide (6b)**

Compound **8** (17.3 g, 59.9 mmol) was dissolved in a mixture of acetonitrile and water (185 mL, 125 mL) and sodium periodate (23.1 g, 108 mmol) was added at 0 °C in small portions. The suspension was stirred for 1 h at rt and the insoluble salts were filtered off. The filtrate was extracted with dichloromethane (3 x 300 mL) and the combined organic layers were dried with $Na_2SO_4$, filtered and the solvent was removed in vacuo. The crude product was dissolved in dichloromethane (180 mL) and *N*-benzylhydroxylamine (9.30 g, 75.5 mmol) and magnesium sulfate (10.8 g) were added. The suspension was stirred over night at rt, filtered and the solvent was removed in vacuo. The crude product was purified by column chromatography (silica gel, hexanes/EtOAc 1:1) to yield **6**

(17.3 g, 80%, d.r. 52:48) as a colorless solid. For analytical characterization small samples of pure diastereomers were obtained by a second column chromatography.

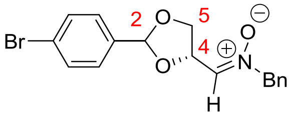


**Diastereomer 6a:**

melting range 105-109 °C; $[\alpha]_D^{22}$ -1.4 (*c* 1.20, $CHCl_3$); $^1H$ NMR (500 MHz, $CDCl_3$): δ 3.87 (dd, *J* = 6.8, 8.5 Hz, 1 H, 5-H), 4.55 (dd, *J* = 7.3, 8.5 Hz, 1 H, 5-H), 4.89 (s, 2 H, $NCH_2$), 5.21-5.27 (m, 1 H, 4-H), 5.84 (s, 1 H, 2-H), 6.93 (d, *J* = 4.5 Hz, 1 H, N=CH), 7.29-7.32 (m, 2 H, Ar), 7.36-7.41 (m, 5 H, Ph), 7.49-7.51 (m, 2 H, Ar) ppm; $^{13}C$ NMR (125 MHz, $CDCl_3$): δ 68.8 (t, $NCH_2$), 69.0 (t, C-5), 72.3 (d, C-4), 103.3 (d, C-2), 123.5 (s, Ar), 128.0, 129.1, 129.3, 129.5, 131.5 ( 5 d, Ph, Ar), 131.8, 136.0 (2 s, Ar, Ph), 138.0 (d, N=CH) ppm; IR (ATR) $\tilde{\nu}$: 3080-2830 (=C-H, C-H), 1600 (C=C, C=N), 1210 (C-O) $cm^{-1}$; ESI-TOF (*m*/*z*): $[M + Na]^+$ calcd for $C_{17}H_{16}BrNO_3Na$, 384.0211; found, 384.0191; $[2M + Na]^+$ calcd for $C_{34}H_{32}Br_2N_2O_6Na$, 747.0504; found, 747.0484; anal. calcd for $C_{17}H_{16}BrNO_3$ (362.2): C, 56.37; H, 4.45; N, 3.87; found: C, 56.38; H, 4.68; N, 4.07.

**Diastereomer 6b:**

melting range 109-114 °C; $[\alpha]_D^{22}$ +7.6 (*c* 1.05, $CHCl_3$); $^1H$ NMR (500 MHz, $CDCl_3$): δ 4.08-4.12 (m, 1 H, 5-H), 4.35 (dd, *J* = 7.9, 8.4 Hz, 1 H, 5-H), 4.85 (s, 2 H, $NCH_2$), 5.23 (td, *J* = 4.7, 7.9 Hz, 1 H, 4-H), 5.75 (s, 1 H, 2-H), 6.84 (d, *J* = 4.7 Hz, 1 H, N=CH), 7.28-7.30 (m, 2 H, Ar), 7.34-7.40 (m, 5 H, Ph), 7.47-7.49 (m, 2 H, Ar) ppm; $^{13}C$ NMR (125 MHz, $CDCl_3$): δ 68.9 (t, $NCH_2$), 69.3 (t, C-5), 72.5 (d, C-4), 103.5 (d, C-2), 123.5 (s, Ar), 128.0, 129.0, 129.2, 129.3, 131.5 (5 d, Ar, Ph), 131.9, 135.6 (2 s, Ar, Ph),

138.2 (d, N=CH) ppm; IR (ATR): $\tilde{\nu}$ 3070-2865 (=C-H, C-H), 1590 (C=C, C=N), 1155 (C-O) $cm^{-1}$; ESI-TOF (*m*/*z*): $[M + Na]^+$ calcd for $C_{17}H_{16}BrNO_3Na$, 384.0211; found, 384.0220; $[2M + Na]^+$ calcd for $C_{34}H_{32}Br_2N_2O_6Na$, 747.0504; found, 747.0510; anal. calcd for $C_{17}H_{16}BrNO_3$ (362.2): C, 56.37; H, 4.45; N, 3.87; found: C, 56.51; H, 4.29; N, 3.90.

**(3*S*)-2-Benzyl-3-[(2*S*,4*S*)-2-(4-bromophenyl)-1,3-dioxolan-4-yl]-4-[2-(trimethylsilyl)-ethoxy]-3,6-dihydro-2*H*-1,2-oxazine (4a) and (3*S*)-2-benzyl-3-[(2*R*,4*S*)-2-(4-bromo-phenyl)-1,3-dioxolan-4-yl]-4-[2-(trimethylsilyl)ethoxy]-3,6-dihydro-2*H*-1,2-oxazine (4b)**

**Procedure 1:**

Under an argon atmosphere, allene **5** (86 mg, 0.55 mmol) was dissolved in THF (2 mL) and cooled to -40 °C. Then *n*-BuLi (0.22 mL, 2.5 M in THF, 0.55 mmol) was added dropwise, the solution was stirred for 10 min at -40 °C and then cooled to -78 °C. Nitrone **6** (100 mg, 0.276 mmol) was dissolved in THF (1 mL) and added dropwise to the solution of deprotonated allene. The mixture was stirred for 1.5 h at -78 °C and then quenched with water (5 mL) at -78 °C. The solution was allowed to warm up to rt and the aqueous layer was extracted with diethyl ether (3 x 50 mL). The combined organic layers were dried with $Na_2SO_4$, filtered and the solvent was removed in vacuo. The crude product was purified by column chromatography (silica gel, hexanes/EtOAc 20:1) to yield the two diastereomers **4a** (50 mg, 35%) and **4b** (46 mg, 32%), both as colorless solids. The yields of reactions in large scale are 43-56%.

**Procedure 2:**

4-Bromobenzaldehyde dimethyl acetal (1.90 mL, 11.4 mmol) and cerium ammonium nitrate (5 mg, 0.010 mmol) were dissolved in dichloromethane (1 mL) and stirred for 15 min at rt. 1,2-Oxazine **10** (400 mg, 1.14 mmol) was added and the mixture was stirring for 3 d at rt. Sat. aq. $NaHCO_3$ solution (50 mL) was added, the layers were separated and the water layer was extracted with dichloromethane (3 x 100 mL). The combined organic layers were dried with $Na_2SO_4$, filtered and the solvent was removed in vacuo. The crude product was purified by column chromatography (silica gel, hexanes/EtOAc 20:1) to yield **4a** (22 mg, 4%) and **4b** (238 mg, 40%), both as colorless solids.

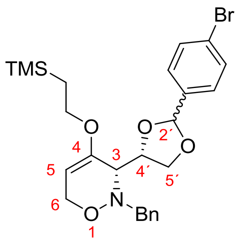


**Diastereomer 4a:**

mp 73-75 °C; $[\alpha]_D^{22}$ +41.0 (*c* 1.00, $CHCl_3$); $^1H$ NMR (500 MHz, $CDCl_3$): δ 0.06 (s, 9 H, $SiMe_3$), 0.98-1.10 (m, 2 H, $Me_3SiCH_2$), 3.32 (d, *J* = 6.5 Hz, 1 H, 3-H), 3.73-3.86 (m, 2 H, $Me_3SiCH_2CH_2$), 4.08-4.20 (m, 5 H, $NCH_2$, 5´-H, 6-H), 4.41-4.45 (m, 1 H, 5´-H), 4.67-4.71 (m, 1 H, 4´-H), 4.76-4.77 (m, 1 H, 5-H), 5.85 (s, 1 H, 2´-H), 7.25-7.27 (m, 1 H, Ph), 7.30-7.33 (m, 2 H, Ph), 7.36-7.37 (m, 2 H, Ar), 7.40-7.42 (m, 2 H, Ph), 7.50-7.51 (m, 2 H, Ar) ppm; $^{13}C$ NMR (125 MHz, $CDCl_3$): δ -1.4 (q, $SiMe_3$), 17.4 (t, $Me_3SiCH_2$), 58.3 (t, $NCH_2$), 63.0 (d, C-3), 63.0 (t, $Me_3SiCH_2CH_2$), 64.5 (t, C-6), 68.0 (t, C-5´), 75.3 (d, C-4´), 93.2 (d, C-5), 102.8 (d, C-2´), 123.0 (s, Ar), 127.1, 128.2, 128.2, 128.7, 131.4 (5 d, Ph, Ar), 137.6, 137.7 (2 s, Ar, Ph), 149.8 (s, C-4) ppm; IR (ATR): $\tilde{\nu}$ 3085 (=C-H), 2950-2830 (C-

H), 1675 (C=C), 1250 (C-O) $cm^{-1}$; ESI-TOF (*m/z*): $[M + Na]^+$ calcd for $C_{25}H_{32}BrNO_4SiNa$, 542.1158; found, 542.1217; $[2M + Na]^+$ calcd for $C_{50}H_{64}Br_2N_2O_8Si_2Na$, 1059.2469; found, 1059.2520; anal. calcd for $C_{25}H_{32}BrNO_4Si$ (518.5): C, 57.91; H, 6.22; N, 2.70; found: C, 57.75; H, 6.15; N, 2.72.

**Diastereomer 4b:**

melting range 84-89 °C; $[\alpha]_D^{22}$ +3.2 (*c* 1.25, $CHCl_3$); $^1H$ NMR (500 MHz, $CDCl_3$): δ 0.03 (s, 9 H, $SiMe_3$), 0.92-1.05 (m, 2 H, $Me_3Si\mathit{CH_2}$), 3.30-3.33 (m, 1 H, 3-H), 3.70-3.75 (m, 1 H, $Me_3SiCH_2\mathit{CH_2}$), 3.77-3.83 (m, 1 H, $Me_3SiCH_2\mathit{CH_2}$), 3.96 (dd, *J* = 6.6, 8.0 Hz, 1 H, 5´-H), 4.07 (t, *J* = 8.0 Hz, 1 H, 5´-H), 4.16-4.21 (m, 3 H, $NCH_2$, 6-H), 4.43-4.47 (m, 1 H, 6-H), 4.68 ($m_c$, 1 H, 4´-H), 4.74-4.76 (m, 1 H, 5-H), 5.82 (s, 1 H, 2´-H), 7.25-7.31 (m, 5 H, Ar, Ph), 7.39-7.40 (m, 2 H, Ar, Ph), 7.44-7.46 (m, 2 H, Ar) ppm; $^{13}C$ NMR (125 MHz, $CDCl_3$): δ -1.5 (q, $SiMe_3$), 17.4 (t, $Me_3Si\mathit{CH_2}$), 58.0 (t, $NCH_2$), 62.9 (d, C-3), 64.4 (t, C-6), 67.1 (t, $Me_3SiCH_2\mathit{CH_2}$), 72.0 (t, C-5´), 76.5 (d, C-4´), 93.1 (d, C-5), 102.9 (d, C-2´), 123.0 (s, Ar), 127.1, 128.2, 128.3, 128.7, 131.3 (5 d, Ar, Ph), 137.4, 137.5 (2 s, Ar, Ph), 149.5 (s, C-4) ppm; IR (ATR): $\tilde{\nu}$ 3065 (=C-H), 2950 (C-H), 1670 (C=C), 1250 (C-O) $cm^{-1}$; ESI-TOF (*m/z*): $[M + Na]^+$ calcd for $C_{25}H_{32}BrNO_4SiNa$, 542.1158; found, 542.1172; $[2M + Na]^+$ calcd for $C_{50}H_{64}Br_2N_2O_8Si_2Na$, 1059.2469; found, 1059.2446; anal. calcd for $C_{25}H_{32}BrNO_4Si$ (518.5): C, 57.91; H, 6.22; N, 2.70; found: C, 57.96; H, 6.32; N, 2.67.

**(1*S*,5*R*,6*R*,8*S*)-2-Benzyl-6-(4-bromophenyl)-8-[(*tert*-butyldimethylsiloxy)methyl]-3,7-dioxa-2-azabicyclo[3.3.1]nonan-9-one (11)**

1,2-Oxazine **4** (5.05 g, 9.74 mmol) was dissolved in acetonitrile (70 mL) and cooled to -30 °C. Tin(IV) chloride (7.61 g, 3.43 mL, 29.2 mmol) was added and the solution was stirred for 18 h and allowed to warm to rt. The mixture was quenched with water (60 mL) and extracted with dichloromethane (3 x 150 mL). The combined organic layers were dried with $Na_2SO_4$, filtered and the solvent was removed in vacuo. The crude product was dissolved in THF (80 mL), imidazole (1.33 g, 19.5 mmol) and *tert*-butyldimethylsilyl chloride (2.20 g, 14.6 mmol) were added and the mixture was stirred for 4 h at rt. The salts were filtered off and the solvent was removed in vacuo. The crude product was extracted with diethyl ether (3 x 150 mL). The combined organic layers were dried with $Na_2SO_4$, filtered, the solvent was removed in vacuo and the crude product was purified by column chromatography (silica gel, hexanes/EtOAc 30:1) to yield **11** (4.22 g, 82%) as a colorless oil.

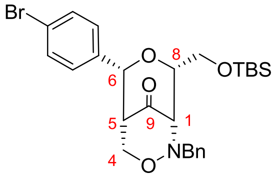


$[\alpha]_D^{22}$ +120.2 (*c* 1.28, $CHCl_3$); $^1$H NMR (500 MHz, $CDCl_3$): δ 0.02, 0.04 (2 s, 3 H each, SiMe), 0.85 (s, 9 H, Si*t*-Bu), 2.71-2.75 (m, 1 H, 5-H), 3.54-3.56 (m, 1 H, 1-H), 3.88 (ddd, *J* = 1.5, 5.1, 7.9 Hz, 1 H, 8-H), 3.94 (dd, *J* = 5.1, 9.5 Hz, 1 H, 8-$CH_2$), 4.01 (d, *J* = 13.8 Hz, 1 H, $NCH_2$), 4.09-4.19 (m, 3 H, 8-$CH_2$, $NCH_2$, 4-H), 4.25 (dd, *J* = 5.6, 12.0 Hz, 1 H, 4-H), 4.89 (s, 1 H, 6-H), 7.25-7.36 (m, 7 H, Ar, Ph), 7.48-7.50 (m, 2 H, Ar) ppm; $^{13}$C NMR (125 MHz, $CDCl_3$): δ -5.32, -5.26 (2 q, SiMe), 18.3, 25.9 (q, s, Si*t*-Bu), 55.3 (d,

C-5), 60.5 (t, $NCH_2$), 61.4 (t, 8-$CH_2$), 67.2 (t, C-4), 70.8 (d, C-1), 81.2 (d, C-6), 81.6 (d, C-8), 122.0 (s, Ar), 127.6, 127.7, 128.6, 128.9, 131.8 (5 d, Ar, Ph), 136.3, 137.2 (2 s, Ar, Ph), 207.9 (s, C-9) ppm; IR (ATR): $\tilde{\nu}$ 3065 (=C-H), 2950-2855 (C-H), 1730 (C=O) $cm^{-1}$; ESI-TOF (*m*/*z*): $[M + H]^+$ calcd for $C_{26}H_{35}BrNO_4Si$, 534.1498; found, 534.1540; $[M + Na]^+$ calcd for $C_{26}H_{34}BrNO_4SiNa$, 556.1318; found, 556.1356; anal. calcd for $C_{26}H_{34}BrNO_4Si$ (532.5): C, 58.64; H, 6.44; N, 2.63; found: C, 57.97; H, 6.51; N, 2.50.

**(1*R*,5*S*,6*R*,8*S*,9*R*)-2-Benzyl-6-(4-bromophenyl)-8-[(*tert*-butyldimethylsiloxy)methyl]-3,7-dioxa-2-azabicyclo[3.3.1]nonan-9-ol (12a) and (1*R*,5*S*,6*R*,8*S*,9*S*)-2-benzyl-6-(4-bromophenyl)-8-[(*tert*-butyldimethylsiloxy)methyl]-3,7-dioxa-2-azabicyclo[3.3.1]nonan-9-ol (12b)**

**Procedure 1:**

At 0 °C compound **11** (500 mg, 0.939 mmol) was dissolved in ethanol (14 mL), sodium borohydride (71 mg, 1.88 mmol) was added and the mixture stirred for 3 h at -40 °C. The solvent was then removed in vacuo and water (50 mL) and dichloromethane (80 mL) were added and the crude product was extracted with dichloromethane (4 x 80 mL). The combined organic layers were dried with $Na_2SO_4$, filtered and the solvent was removed in vacuo. The crude product was purified by column chromatography (silica gel, hexanes/EtOAc 5:1) to yield **12a** (315 mg, 63%) and **12b** (46 mg, 9%) as colorless solids.

**Procedure 2:**

Compound **11** (680 mg, 1.28 mmol) was dissolved in THF (20 mL), at -10 °C L-selectride (1.92 mL, 1 M in THF, 1.92 mmol) was added dropwise and the solution was stirred for 1 h at -10 °C. The mixture was quenched with sat. aq. $NH_4Cl$ solution (30 mL) and the aqueous layer was extracted with diethyl ether (4 x 80 mL). The combined organic layers were dried with $Na_2SO_4$, filtered and the solvents removed in vacuo. The crude product was purified by column chromatography (silica gel, hexanes/EtOAc 5:1) to yield **12a** (501 mg, 73%) as a colorless solid.

**Diastereomer 12a:**

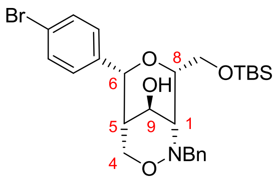


mp 140-143 °C; $[\alpha]_D^{22}$ +25.2 (*c* 1.04, $CHCl_3$); $^1$H NMR (500 MHz, $CDCl_3$): δ 0.11, 0.12 (2 s, 3 H each, SiMe), 0.93 (s, 9 H, Si*t*-Bu), 2.05 ($s_{br}$, 1 H, 5-H), 3.30 ($s_{br}$, 1 H, 1-H), 3.68 (dd, *J* = 1.3, 12.2 Hz, 1 H, 4-H), 3.76 (d, *J* = 10.7 Hz, 1 H, OH), 3.85 (dd, *J* = 6.9, 8.9 Hz, 1 H, 8-H), 3.98-4.07 (m, 4 H, 4-H, 9-H, 8-$CH_2$), 4.13 (AB system, $J_{AB}$ = 15.1 Hz, 1 H, $NCH_2$), 4.35 (AB system, $J_{AB}$ = 15.1 Hz, 1 H, $NCH_2$), 4.74 (s, 1 H, 6-H), 7.24-7.29 (m, 3 H, Ph), 7.32-7.36 (m, 4 H, Ar, Ph), 7.45-7.47 (m, 2 H, Ar) ppm; $^{13}$C NMR (125 MHz, $CDCl_3$): δ -5.4, -5.3 (2 q, SiMe), 18.1, 25.8 (q, s, Si*t*-Bu), 40.8 (d, C-5), 60.2 (d, C-1), 61.6 (t, $NCH_2$), 62.4 (t, 8-$CH_2$), 64.8 (t, C-4), 70.3 (d, C-8), 78.9 (d, C-6), 79.7 (d, C-9), 121.0 (s, Ar), 127.1, 127.5, 128.0, 128.3, 131.3 (5 d, Ar, Ph), 138.3 139.0 (2 s, Ar, Ph) ppm; IR (ATR): $\tilde{\nu}$ 3555-3135 (O-H), 3095-3030 (=C-H), 2960-2855 (C-H), 1250 (C-O) $cm^{-1}$; ESI-TOF (*m*/*z*): $[M + H]^+$ calcd for $C_{26}H_{37}BrNO_4Si$, 534.1675; found, 534.1680;

[M + Na]$^+$ calcd for $C_{26}H_{36}BrNO_4SiNa$, 558.1474; found, 558.1481; anal. calcd for $C_{26}H_{36}BrNO_4Si$ (534.6): C, 58.42; H, 6.79; N, 2.62; found: C, 58.31; H, 5.98; N, 2.98.

**Diastereomer 12b:**

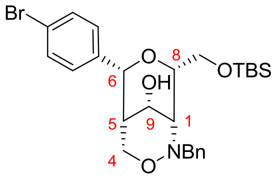


Melting range 161-165 °C; $[\alpha]_D^{22}$ +61.7 (*c* 1.37, $CHCl_3$); $^1H$ NMR (500 MHz, $CDCl_3$): δ 0.05, 0.07 (2 s, 3 H each, SiMe), 0.87 (s, 9 H, Si*t*-Bu), 1.85 ($s_{br}$, 1 H, 5-H), 2.98 ($s_{br}$, 1H, 1-H), 3.67 (d, *J* = 12.2 Hz, 1 H, 4-H), 3.90-4.01 (m, 4 H, 4-H, 8-$CH_2$, OH), 4.09 (t, *J* = 8.9 Hz, 1H, 8-H), 4.16 (AB system, $J_{AB}$ = 14.3 Hz, 1 H, $NCH_2$), 4.29 (AB system, $J_{AB}$ = 14.3 Hz, 1 H, $NCH_2$), 4.68 (t, *J* = 3.7 Hz, 1 H, 9-H), 5.27 (s, 1 H, 6-H), 7.23-7.36 (m, 7 H, Ar, Ph), 7.42-7.49 (m, 2 H, Ar) ppm; $^{13}C$ NMR (125 MHz, $CDCl_3$): δ -5.3, -5.2 (2 q, SiMe), 18.3, 25.9 (q, s, Si*t*-Bu), 41.5 (d, C-5), 57.6 (d, C-1), 58.2 (t, $NCH_2$), 62.4 (d, C-8), 62.8 (t, 8-$CH_2$), 64.1 (d, C-9), 64.3 (t, C-4), 72.7 (d, C-6), 79.7 (d, C-9), 120.8 (s, Ar), 127.2, 127.9, 128.3, 128.6, 131.2 (5 d, Ar, Ph), 131.3, 131.6 (2 s, Ar, Ph) ppm; IR (ATR): $\tilde{\nu}$ 3515-3340 (O-H), 3090-3030 (=C-H), 2950-2855 (C-H), 1250 (C-O) $cm^{-1}$; ESI-TOF (*m*/*z*): [M + H]$^+$ calcd for $C_{26}H_{37}BrNO_4Si$, 534.1675; found, 534.1678; [M + Na]$^+$ calcd for $C_{26}H_{36}BrNO_4SiNa$, 558.1474; found, 558.1478; anal. calcd for $C_{26}H_{36}BrNO_4Si$ (534.6): C, 58.42; H, 6.79; N, 2.62; found: C, 59.51; H, 6.69; N, 2.44.

**(1*S*,5*R*,6*R*,8*S*,9*R*)-2-Benzyl-6-(4-bromophenyl)-9-(*tert*-butyldimethylsiloxy)-8-[(*tert*-butyldimethylsiloxy)methyl]-3,7-dioxa-2-azabicyclo[3.3.1]nonane (13)**

Compound **12a** (277 mg, 0.518 mmol) was dissolved in dichloromethane (1.5 mL), 2,6-lutidine (0.11 mL, 0.932 mmol) was added and the mixture was cooled to 0 °C. *tert*-butyldimethylsilyl triflate (0.12 mL, 0.673 mmol) was added dropwise and the solution was stirred for 2 h at 0 °C. The mixture was quenched with sat. aq. $NH_4Cl$ solution (20 mL) and the aqueous layers were extracted with dichloromethane (3 x 30 mL). The combined organic layers were dried with $Na_2SO_4$, filtered and the solvent was removed in vacuo. The crude product was purified by column chromatography (silica gel, hexanes/EtOAc 30:1) to yield **13** (336 mg, quant.) as a colorless oil.

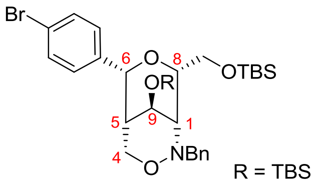


$[\alpha]_D^{22}$ +71.5 (*c* 1.12, $CHCl_3$); $^1$H NMR (500 MHz, $CDCl_3$): δ 0.06, 0.08, 0.17, 0.19 (4 s, 3 H each, SiMe), 0.88, 1.00 (2 s, 9 H each, Si*t*-Bu),1.65 ($m_c$, 1 H, 5 H), 3.00 ($s_{br}$, 1 H, 1-H), 3.22 (d, *J* = 11.8 Hz, 1 H, 4-H), 3.85 (ddd, *J* = 1.8, 5.1, 7.2 Hz, 1 H, 8-H), 3.92 (dd, *J* = 5.1, 9.6 Hz, 1 H, 8-$CH_2$), 4.10 ($m_c$, 1 H, 9-H), 4.11 (d, *J* = 14.7 Hz, 1 H, $NCH_2$), 4.17 (dd, *J* = 7.2, 9.6 Hz, 1 H, 8-$CH_2$), 4.47 (dt, *J* = 1.7, 11.8 Hz, 1 H, 4-H), 4.86 (d, *J* = 14.7 Hz, 1 H, $NCH_2$), 4.87 ($s_{br}$, 1 H, 6-H), 7.21-7.24 (m, 1 H, Ph), 7.29-7.32 (m, 4 H, Ph), 7.38-7.39 (m, 2 H, Ar), 7.43-7.45 (m, 2 H, Ar) ppm; $^{13}$C NMR ($CDCl_3$, 125 MHz): δ -5.13, -5.06, -4.7, -4.5 (4 q, SiMe), 18.2, 18.4 (2 s, Si*t*-Bu), 25.9, 26.1 (2 q, Si*t*-Bu), 42.4 (d, C-5), 56.0 (t, C-4), 58.7 (t, $NCH_2$), 58.8 (d, C-1), 63.3 (t, 8-$CH_2$), 70.4 (d, C-9), 79.8 (d, C-6), 80.2 (d, C-8), 121.3 (s, Ar), 127.0, 128.3, 128.3, 128.6, 131.4 (5 d, Ph, Ar), 139.4, 139.6 (2 s,

Ph, Ar) ppm; IR (ATR): $\tilde{\nu}$ 3090-3025 (=C-H), 2955-2855 (C-H), 1250 (C-O) $cm^{-1}$; ESI-TOF (*m*/*z*): $[M + H]^+$ calcd for $C_{32}H_{51}BrNO_4Si_2$, 648.2535; found, 648.2574; $[M + Na]^+$ calcd for $C_{32}H_{50}BrNO_4Si_2Na$, 670.2354, found, 670.2395; anal. calcd for $C_{32}H_{50}BrNO_4Si_2$ (648.8): C, 59.24; H, 7.77; N, 2.16; found: C, 59.48; H, 7.78; N, 2.16.

**(1*R*,5*S*,6*R*,8*S*,9*R*)-2-Benzyl-6-(4-bromophenyl)-8-(hydroxymethyl)-3,7-dioxa-2-azabicyclo[3.3.1]nonan-9-ol (15a) and (1*R*,5*S*,6*R*,8*S*,9*S*)-2-benzyl-6-(4-bromo-phenyl)-8-(hydroxymethyl)-3,7-dioxa-2-azabicyclo[3.3.1]nonan-9-ol (15b)**

1,2-Oxazine **4** (2.00 g, 3.86 mmol) was dissolved in acetonitrile (20 mL) and cooled to −30 °C. Tin(IV) chloride (3.02 g, 1.36 mL, 11.6 mmol) was added and the solution was stirred for 18 h and allowed to warm up to rt. The mixture was quenched with water (40 mL) and the aqueous layer extracted with dichloromethane (3 x 80 mL). The combined organic layers were dried with $Na_2SO_4$, filtered and the solvent was removed in vacuo. The crude product was dissolved in THF (30 mL) and cooled to -15 °C. L-selectride (4.63 mL, 1 M in THF, 4.63 mmol) was added dropwise and the solution was stirred for 1 h at -15 °C. The mixture was quenched with sat. aq. $NH_4Cl$ solution (50 mL) and the aqueous layer extracted with diethyl ether (5 x 80 mL). The combined organic layers were dried with $Na_2SO_4$, filtered and the solvent was removed in vacuo. The crude product was purified by column chromatography (silica gel, hexanes/EtOAc 1:1 → 1:2) to yield **15a** (254 mg, 16%) and **15b** (685 mg, 42%) as colorless solids.

**Diastereomer 15a:**

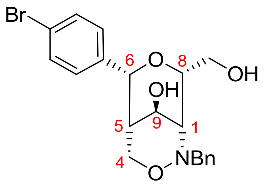

mp 58-60 °C; $[\alpha]_D^{22}$ +88.9 (*c* 1.00, $CHCl_3$); $^1$H NMR (500 MHz, $CDCl_3$): δ 2.02 ($m_c$, 1 H, 5-H), 2.60 ($s_{br}$, 1 H, OH), 3.08 ($m_c$, 1 H, 1-H), 3.60 ($s_{br}$, 1 H, OH), 3.66 (dd, *J* = 2.3, 12.3 Hz, 1 H, 4-H), 3.81-3.85 (m, 2 H, 8-H, 8-$CH_2$), 4.03-4.13 (m, 3 H, 4-H, 8-$CH_2$, 9-H), 4.09 (AB system, $J_{AB}$= 14.0 Hz, 1 H, $NCH_2$), 4.29 (AB system, $J_{AB}$= 14.0 Hz, 1 H, $NCH_2$), 4.75 (s, 1 H, 6-H), 7.24-7.29 (m, 3 H, Ph), 7.33-7.34 (m, 4 H, Ar, Ph), 7.45-7.48 (m, 2 H, Ar) ppm; $^{13}$C NMR (125 MHz, $CDCl_3$): δ 40.8 (d, C-5), 61.3 (d, C-1), 61.6 (t, $NCH_2$), 64.0 (t, 8-$CH_2$), 64.2 (t, C-4), 70.3 (d, C-9), 79.2 (d, C-6), 79.9 (d, C-8), 121.4 (s, Ar), 127.7, 128.6, 128.7, 131.6 (4 d, Ar, Ph), 137.3, 138.9 (2 s, Ar, Ph) ppm; IR (ATR): $\tilde{\nu}$ 3530-3210 (O-H), 2920-2850 (C-H), 1070 (C-O) $cm^{-1}$; ESI-TOF (*m/z*): $[M + H]^+$ calcd for $C_{20}H_{23}BrNO_4$, 420.0810; found, 420.0807; $[M + Na]^+$ calcd for $C_{20}H_{22}BrNO_4Na$, 442.0630; found, 442.0630; anal. calcd for $C_{20}H_{22}BrNO_4$ (420.3): C, 57.15; H, 5.28; N, 3.33; found: C, 57.30; H, 5.42; N, 3.48.

**Diastereomer 15b:**

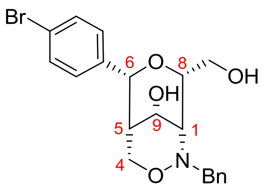


mp 197-198 °C; $[\alpha]_D^{22}$ +125.4 (*c* 0.95, $CHCl_3$/MeOH, 9:1); $^1$H NMR ($CDCl_3$/$CD_3OD$, 6:1, 700 MHz): δ 1.81 ($m_c$, 1 H, 5-H), 2.80 ($s_{br}$, 1 H, 1-H), 3.61 (dd, *J* = 1.4, 12.3 Hz, 1 H, 4-H), 3.79 (dd, *J* = 4.2, 11.6 Hz, 1 H, 8-$CH_2$), 3.98 (dd, *J* = 5.7, 11.6 Hz, 1 H, 8-$CH_2$), 4.02 (ddd, *J* = 1.4, 2.6, 12.3 Hz, 1 H, 4-H), 4.08 (AB system, *J* = 13.5 Hz, 1 H, $NCH_2$), 4.26 (AB system, *J* = 13.5 Hz, 1 H, $NCH_2$), 4.26 ($m_c$, 1 H, 8-H), 4.85 (t, *J* = 3.8 Hz, 1 H, 9-H), 5.25 (s, 1 H, 6-H), 7.21-7.73 (m, 1 H, Ph), 7.26-7.30 (m, 6 H, Ar, Ph), 7.40-7.41 (m, 2 H, Ar) ppm; $^{13}$C NMR ($CDCl_3$/$CD_3OD$, 6:1, 175 MHz): δ 41.2 (d, C-5), 57.2 (t, $NCH_2$), 58.7

(d, C-1), 61.5 (d, C-9), 63.7 (t, C-4), 64.5 (t, 8-$CH_2$), 72.8 (d, C-8), 73.0 (d, C-6), 120.9 (s, Ar), 127.8, 127.9, 128.5, 128.6, 131.1 (5 d, Ar, Ph), 137.0, 140.0 (2 s, Ar, Ph) ppm; IR (ATR): $\tilde{\nu}$ 3385 (O-H), 3085-3025 (=CH), 2920-2870 (CH), 1490 (CH) $cm^{-1}$; ESI-TOF (*m*/*z*): $[M + H]^+$ calcd for $C_{20}H_{23}BrNO_4$, 420.0810; found, 420.0824; $[M + Na]^+$ calcd for $C_{20}H_{22}BrNO_4Na$, 442.0630; found, 442.0652; anal. calcd for $C_{20}H_{22}BrNO_4$ (420.3): C, 57.15; H, 5.28; N, 3.33; found: C, 57.52; H, 5.32; N, 3.37.

**(1*R*,5*S*,6*R*,8*S*,9*R*)-2-Benzyl-6-(4-bromophenyl)-8-(trityloxymethyl)-3,7-dioxa-2-azabicyclo[3.3.1]nonan-9-ol (16)**

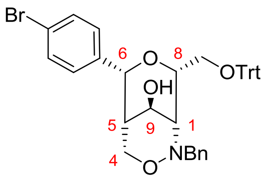


Compound **15a** (360 mg, 0.857 mmol) was dissolved in pyridine (4 mL). Trityl chloride (287 mg, 1.03 mmol) and DMAP (42 mg, 0.343 mmol) were added and the mixture was stirred for 3 d at 60 °C. The mixture was quenched with brine (10 mL) and the aqueous layer was extracted with ethyl acetate (3 x 30 mL). The combined organic layers were dried with $Na_2SO_4$, filtered and the solvents removed in vacuo. The crude product was purified by column chromatography (silica gel, hexanes/EtOAc 4:1) to yield **16** (470 mg, 83%) as a colorless solid.

mp 94-96 °C; $[\alpha]_D^{22}$ +17.8 (*c* 1.10, $CHCl_3$); $^1H$ NMR (500 MHz, $CDCl_3$): δ 2.01 ($s_{br}$, 1 H, 5-H), 3.30 (s, 1 H, 1-H), 3.34 (dd, *J* = 7.8, 8.8 Hz, 1 H, 8-$CH_2$), 3.60 (d, *J* = 12.2 Hz, 1 H,

4-H), 3.83 (dd, $J$ = 6.3, 8.8 Hz, 1 H, 8-$CH_2$), 3.84 (AB system, $J$ = 14.9 Hz, 1 H, $NCH_2$), 3.91 (AB system, $J$ = 14.9 Hz, 1 H, $NCH_2$), 3.96 (dd, $J$ = 4.7, 12.2 Hz, 1 H, 4-H), 4.12 ($m_c$, 2 H, 9-H, 8-H), 4.80 (s, 1 H, 6-H), 7.05-7.07 (m, 2 H, Ph, Ar), 7.22-7.28 (m, 14 H, Ph, Ar), 7.45-7.51 (m, 8 H, Ph, Ar) ppm; $^{13}$C NMR (125 MHz, $CDCl_3$): δ 41.1 (d, C-5), 60.7 (d, C-1), 61.2 (t, $NCH_2$), 64.1 (t, 8-$CH_2$), 64.9 (t, C-4), 70.2 (d, C-9), 78.7 (d, C-8), 79.2 (d, C-6), 87.0 (s, $CPh_3$), 121.2 (s, Ar), 127.2, 127.3, 127.7, 128.0, 128.1, 128.2, 128.3, 128.7, 131.4 (9 d, Ar, Ph), 137.9, 139.2, 143.8 (3 s, Ph, Ar) ppm; IR (ATR): $\tilde{\nu}$ 3555-3300 (O-H), 3025 (=C-H), 2940-2855 (C-H), 1450 ($CH_2$) $cm^{-1}$; ESI-TOF (*m*/*z*): $[M + H]^+$ calcd for $C_{39}H_{37}BrNO_4$, 662.1906; found, 662.1904; $[M + Na]^+$ calcd for $C_{39}H_{36}BrNO_4Na$, 684.1725; found, 684.1721; anal. calcd for $C_{39}H_{36}BrNO_4$ (662.6): C, 70.69; H, 5.48; N, 2.11; found: C, 71.21; H, 6.59; N, 2.13.

**[(2*S*,3*R*,4*R*,5*S*,6*R*)-3-Amino-2,5-di(hydroxymethyl)-6-phenyltetrahydro-2*H*-pyran-4-ol (17a)**

A suspension of Pd/C (10% Pd, 50 mg) and iPrOH (3 mL) was saturated with hydrogen for 15 min. Compound **15a** (50 mg, 0.119 mmol) and $NEt_3$ (12 mg, 0.119 mmol) were dissolved in EtOAc (1 mL) and added to this suspension. The mixture was stirred for 18 h under hydrogen pressure (balloon), filtered through a pad of Celite® and the solvent removed in vacuo. The crude product was purified by column chromatography (silica gel, $CH_2Cl_2$/MeOH 15:1) to yield **17a** (25 mg, 83%) as a colorless solid.

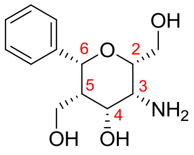

mp 163 °C; $[\alpha]_D^{22}$ +71.5 (*c* 0.60, $CH_3OH$); $^1$H NMR (500 MHz, $CD_3OD$): δ 2.21 ($s_{br}$, 1 H, 5-H), 3.19 (dd, *J* = 4.2, 11.5 Hz, 1 H, 5-$CH_2$), 3.24 (d, *J* = 1.6 Hz, 1 H, 3-H), 3.59-3.64 (m, 2 H, 5-$CH_2$, 2-H), 3.71 (dd, *J* = 5.5, 11.3 Hz, 1 H, 2-$CH_2$), 3.83 (dd, *J* = 6.6, 11.3 Hz, 1 H, 2-$CH_2$), 4.27 (dd, *J* = 4.4, 5.5 Hz, 1 H, 4-H), 4.68 (d, *J* = 2.6 Hz, 1 H, 6-H), 7.21 (C part of AA´BB´C system, $J_{CB}$ = 7.5 Hz, 1 H, Ph), 7.30 (B part of AA´BB´C system, $J_{ABC}$ = 7.5 Hz, 2 H, Ph), 7.44 (A part of AA´BB´C system, $J_{AB}$ = 7.5 Hz, 2 H, Ph) ppm; $^{13}$C NMR ($CD_3OD$, 175 MHz): δ 47.3 (d, C-5), 50.8 (d, C-3), 56.2 (t, 5-$CH_2$), 63.1 (t, 2-$CH_2$), 73.1 (d, C-4), 80.4 (d, C-2), 82.0 (d, C-6), 127.1, 127.9, 128.9, 141.6 (3 d, s, Ph) ppm; IR (ATR): $\tilde{\nu}$ 3600-3300 (O-H, N-H), 3070-3025 (=C-H), 2930-2855 (C-H) $cm^{-1}$; ESI-TOF (*m*/*z*): $[M + H]^+$ calcd for $C_{13}H_{20}NO_4$, 254.1392; found, 254.1408; $[M + Na]^+$ calcd for $C_{13}H_{19}NO_4Na$, 276.1212; found, 276.1223; anal. calcd for $C_{13}H_{19}NO_4$ (253.3): C, 61.64; H, 7.56; N, 5.53; C, 61.60; H, 7.65; N, 5.66.

**(2*S*,3*R*,4*S*,5*S*,6*R*)-3-Amino-2,5-di(hydroxymethyl)-6-phenyltetrahydro-2*H*-pyran-4-ol (17b)**

A suspension of Pd/C (10% Pd, 69 mg) and iPrOH (3 mL) was saturated with hydrogen for 15 min. Compound **15b** (69 mg, 0.164 mmol) and $NEt_3$ (17 mg, 0.164 mmol) were dissolved in EtOAc (1 mL) and added to this suspension. The mixture was stirred for 18 h under hydrogen pressure (balloon), then filtrated through a pad of Celite® and the solvent was removed in vacuo. The crude product was purified by column chromatography (silica gel, $CH_2Cl_2$/MeOH 15:1) to yield **17b** (32 mg, 77%) as a colorless solid.

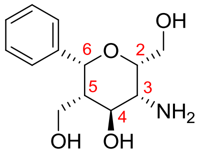


mp 185-188 °C, $[\alpha]_D^{22}$ +46.1 (*c* 1.09, $CH_3OH$); $^1$H NMR (500 MHz, $CD_3OD$): δ 2.06 (dd, *J* = 4.2, 7.5 Hz, 1 H, 5-H), 3.39 (dd, *J* = 4.2, 11.1 Hz, 1 H, 5-$CH_2$), 3.44 (d, *J* = 4.5 Hz, 1 H, 3-H), 3.45 (dd, *J* = 2.9, 11.1 Hz, 1 H, 5-$CH_2$), 3.93 ($d_{br}$, *J* = 4.7 Hz, 2 H, 2-$CH_2$), 4.17 (td, *J* = 1.8, 4.7 Hz, 1 H, 2-H), 4.32 ($s_{br}$, 1 H, 4-H), 5.17 (d, *J* = 4.2 Hz, 1 H, 6-H), 7.26 (C part of AA´BB´C system, $J_{CB}$ = 7.3 Hz, 1 H, Ph), 7.35 (A part of AA´BB´C system, $J_{AB}$ = 7.3 Hz, 2 H, Ph), 7.43 (B part of AA´BB´C system $J_{BAC}$ = 7.3 Hz, 2 H, Ph) ppm; $^{13}$C NMR (125 MHz, $CD_3OD$): δ 47.1 (d, C-5), 51.7 (d, C-3), 60.3 (t, 5-$CH_2$), 63.6 (t, 2-$CH_2$), 70.8 (d, C-4), 74.1 (d, C-2), 79.2 (d, C-6), 126.8, 128.1, 129.1, 141.0 (3 d, s, Ph) ppm; IR (ATR): $\tilde{\nu}$ 3435, 3245 (O-H, N-H), 3020 (=C-H), 2930-2830 (C-H) $cm^{-1}$; ESI-TOF (*m*/*z*): $[M + H]^+$ calcd for $C_{13}H_{20}NO_4$, 254.1392; found, 254.1394; $[M + Na]^+$ calcd for $C_{13}H_{19}NO_4Na$, 276.1212; found, 276.1206.

**(1*R*,5*S*,6*R*,8*S*,9*R*)-2-Benzyl-6-(biphenyl-4-yl)-8-(hydroxymethyl)-3,7-dioxa-2-azabicyclo[3.3.1]nonan-9-ol (18)**

Compound **15a** (290 mg, 0.690 mmol), $Pd(PPh_3)_4$ (40 mg, 34.5 µmol) and phenylboronic acid (93 mg, 0.759 mmol) were filled in a sealed tube and flushed with argon. THF (3 mL) and 2 M $Na_2CO_3$ solution (0.69 mL) were added and the mixture was stirred for 48 h at 70 °C. The solution was cooled to rt, water (20 mL) added and the aqueous layer extracted with ethyl acetate (3 x 80 mL). The combined organic layers were dried with $Na_2SO_4$, filtered and the solvents removed in vacuo. The crude product was purified by

column chromatography (silica gel, hexanes/EtOAc 1:1) to yield **18** (234 mg, 81%) as a colorless solid.

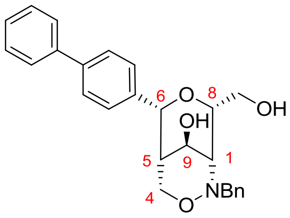


mp 173-175 °C; $[\alpha]_D^{22}$ +91.9 (*c* 1.1, $CH_3OH$); $^1$H NMR (500 MHz, $CDCl_3$): δ 2.12 (s, 1 H, 5-H), 2.28 ($s_{br}$, 1 H, OH), 3.11 (s, 1 H, 1-H), 3.71 (d, *J* = 10.0 Hz, 1 H, OH), 3.81 (dd, *J* = 1.7, 12.1 Hz, 1 H, 4-H), 3.86-3.89 (m, 2 H, 8-H, 8-$CH_2$), 4.08-4.11 (m, 1 H, 9-H), 4.12-4.13 (m, 1 H, 4-H), 4.14 (d, *J* = 14.1 Hz, 1 H, $NCH_2$), 4.17 (dd, *J* = 8.0, 12.4 Hz, 1 H, 8-$CH_2$), 4.33 (d, *J* = 14.1 Hz, 1 H, $NCH_2$), 4.85 (s, 1 H, 6-H), 7.28-7.31 (m, 1 H, Ph), 7.34-7.39 (m, 5 H, Ph, Ar), 7.43-7.48 (m, 4 H, Ph, Ar), 7.58-7.60 (m, 4 H, Ar) ppm; $^{13}$C NMR (125 MHz, $CDCl_3$): δ 40.9 (d, C-5), 61.4 (d, C-1), 61.5 (t, $NCH_2$), 64.0 (t, 8-$CH_2$), 64.3 (t, C-4), 70.4 (d, C-9), 79.6 (d, C-6), 79.8 (d, C-8), 126.4, 127.1, 127.2, 127.4, 127.6, 128.6, 128.7, 128.9 (8 d, Ar, Ph), 137.4, 138.9, 140.4, 140.8 (4 s, Ar, Ph) ppm; IR (ATR): $\tilde{\nu}$ 3610-3180 (O-H), 3090-3010 (=C-H), 2950-2850 (C-H), 1240 (C-O) $cm^{-1}$; ESI-TOF (*m/z*): [M + H] calcd for $C_{26}H_{28}NO_4$, 418.2013; found, 418.2015; [M + Na] calcd for $C_{26}H_{27}NO_4Na$, 440.1838; found, 440.1831; anal. calcd for $C_{26}H_{27}NO_4$ (417.5): C, 74.80; H, 6.52; N, 3.35; found: C, 74.80; H, 6.67; N, 3.73.

**(2*S*,3*R*,4*R*,5*S*,6*R*)-3-Amino-6-(biphenyl-4-yl)-2,5-di(hydroxymethyl)tetrahydro-2*H*-pyran-4-ol (19)**

A suspension of Pd/C (10% Pd, 55 mg) and iPrOH (3 mL) was saturated with hydrogen for 15 min. The bicyclic compound **18** (55 mg, 0.132 mmol) was dissolved in THF

(1 mL), and added to the suspension. The mixture was stirred for 24 h under hydrogen pressure (balloon). The mixture was then filtrated through a pad of Celite® and the solvent was removed in vacuo. The crude material was purified by column chromatography (silica gel, $CH_2Cl_2$/MeOH 7:1) to yield **19** (26 mg, 60%) as a colourless solid.

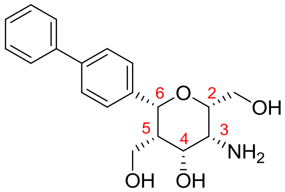


Decomposition >230 °C; $[\alpha]_D^{22}$ +44.4 (*c* 1.00, $CH_3OH/CHCl_3$, 7:3); $^1H$ NMR (700 MHz, $CD_3OD/CDCl_3$, 2:1): δ 2.40 (dt, *J* = 3.0, 6.7 Hz, 1 H, 5-H), 3.40 (dd, *J* = 3.0, 11.1 Hz, 1 H, 5-$CH_2$), 3.73 (dd, *J* = 1.7, 4.5 Hz, 1 H, 3-H), 3.88-3.90 (m, 2 H, 2-H, 5-$CH_2$), 4.07 ($m_c$, 2 H, 2-$CH_2$), 4.64 (dd, *J* = 4.5, 6.7 Hz, 1 H, 4-H), 4.98 (d, *J* = 3.6 Hz, 1 H, 6-H), 7.43-7.45 (m, 1 H, Ph), 7.53-7.55 (m, 2 H, Ph), 7.63-7.64 (m, 2 H, Ar), 7.70-7.72 (m, 4 H, Ph, Ar) ppm; $^{13}C$ NMR (175 MHz, $CD_3OD/CDCl_3$, 2:1): δ 45.5 (d, C-5), 51.6 (d, C-3), 55.0 (t, 5-$CH_2$), 63.1 (t, 2-$CH_2$), 69.0 (d, C-4), 76.8 (d, C-2), 81.7 (d, C-6), 127.0, 127.4, 127.6, 128.0, 129.5 (5 d, Ph), 139.0, 141.1, 141.6 (3 s, Ph, Ar) ppm; IR (ATR): $\tilde{\nu}$ 3480-3240 (O-H, N-H), 3045-3030 (=C-H), 2950-2850 (C-H) $cm^{-1}$; ESI-TOF (*m*/*z*): $[M + H]^+$ calcd for $C_{19}H_{24}NO_4$, 330.1705; found, 330.1713; $[M + Na]^+$ calcd for $C_{19}H_{23}NO_4Na$, 352.1525; found, 352.1518.

***p*-Terphenyl derivative 21**

Compound **12a** (400 mg, 0.748 mmol), $Pd(PPh_3)_4$ (86 mg, 74.8 µmol) and benzene-1,4-diboronic acid (59 mg, 0.36 mmol) were filled in a sealed tube and flushed with argon.

THF (2 mL), DMF (8 mL) and 2 M aq. $Na_2CO_3$ solution (1.5 mL) were added and the mixture was stirred for 48 h at 70 °C. The solution was cooled to rt, brine (20 mL) was added and the aqueous layer extracted with ethyl acetate (3 x 80 mL). The combined organic layers were dried with $Na_2SO_4$, filtered and the solvents removed in vacuo. The crude product was purified by column chromatography (silica gel, hexanes/EtOAc 3:2) to yield **21** (296 mg, 84%) as a yellow solid.

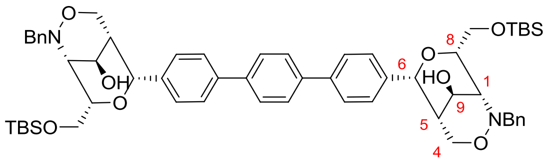


mp 163-165 °C; $[\alpha]_D^{22}$ + 55.6 (*c* 1.10, $CHCl_3$); $^1$H NMR (500 MHz, $CDCl_3$): δ 0.12, 0.13 (2 s, 6 H each, SiMe), 0.94 (s, 18 H, Si*t*-Bu), 2.15 ($s_{br}$, 2 H, 5-H), 3.33 (s, 2 H, 1-H), 3.80 (d, *J* = 10.9 Hz, 2 H, OH), 3.84 (dd, *J* = 1.9, 12.0 Hz, 2 H, 4-H), 3.88 (ddd, *J* = 1.0, 5.8, 9.0 Hz, 2 H, 8-H), 4.01-4.12 (m, 8 H, 8-$CH_2$, 9-H, 4-H), 4.15, 4.39 (AB system, $J_{AB}$ = 15.1 Hz, 4 H, $NCH_2$), 4.84 (s, 2 H, 6-H), 7.28-7.29 (m, 2 H, Ar), 7.33-7.38 (m, 8 H, Ph), 7.46 (AB part of AA´BB´ system, $J_{AB}$ = 8.3 Hz, 4 H, Ar), 7.61 (A´B´ part of AA´BB´ system $J_{A'B'}$= 8.3 Hz, 4 H, Ar), 7.65 (s, 4 H, Ar) ppm; $^{13}$C NMR (125 MHz, $CDCl_3$): δ -5.2, -5.1 (2 q, SiMe), 18.3, 26.0 (s, q, Si*t*-Bu), 41.2 (d, C-5), 60.6 (d, C-1), 61.9 (t, $NCH_2$), 62.6 (t, 8-$CH_2$), 65.4 (t, C-4), 70.7 (d, C-9), 79.6 (d, C-6), 79.9 (d, C-8), 126.4, 127.0, 127.3, 127.5, 128.2, 128.5 (6 d, Ar, Ph), 138.6, 139.3, 139.8, 139.8 (4 s, Ar, Ph) ppm; IR (ATR): $\tilde{\nu}$ 3615-3155 (O-H), 3085-3030 (=C-H), 2955-2855 (C-H), 1250 (C-O) $cm^{-1}$; ESI-TOF (*m*/*z*): $[M + H]^+$ calcd for $C_{58}H_{77}N_2O_8Si_2$, 985.5219; found, 985.5216; $[M + Na]^+$ calcd for $C_{58}H_{76}N_2O_8Si_2Na$, 1007.5038; found, 1007.5032; anal. calcd for $C_{58}H_{76}N_2O_8Si_2$ (985.4): C, 70.69; H, 7.77; N 2.84; found, C, 70.71; H, 7.49; N, 3.25.

**$p$-Terphenyl derivative 22**

Compound **21** (50 mg, 0.051 mmol) was dissolved in THF (1 mL), AcOH (1 mL) and $H_2O$ (0.1 mL). Zinc (67 mg, 1.02 mmol) was added and the mixture was heated to 60 °C for 18 h. The salts were filtered off and the solvent was removed in vacuo. The crude product was purified by column chromatography [silica gel, $CH_2Cl_2$/MeOH (7 N $NH_3$) 10:1] to yield **22** (23 mg, 59%) as a colorless solid.

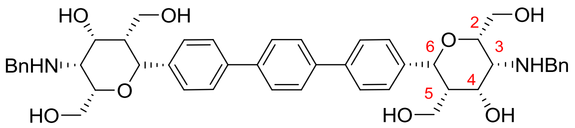


mp 135-137 °C; $[\alpha]_D^{22}$ +57.1 ($c$ 1.05, $CH_3OH/C_5H_5N$, 9:1); $^1H$ NMR (700 MHz, $CD_3OD/CDCl_3$ 5:1): δ 2.27 ($m_c$, 2 H, 5-H), 3.16 (dd, $J$ = 1.8, 3.9 Hz, 2 H, 3-H), 3.37 (dd, $J$ = 4.7, 11.6 Hz, 2 H, 5-$CH_2$), 3.65 (td, $J$ = 1.8, 5.2 Hz, 2 H, 2-H), 3.74 (dd, $J$ = 2.8, 11.6 Hz, 2 H, 5-$CH_2$), 3.84 (dd, $J$ = 4.8, 11.7 Hz, 2 H, 2-$CH_2$), 3.90 (dd, $J$ = 5.2, 11.7 Hz, 2 H, 2-$CH_2$), 3.95 (d, $J$ = 12.6 Hz, 2 H, $NCH_2$), 4.12 (d, $J$ = 12.6 Hz, 2 H, $NCH_2$), 4.39 (dd, $J$ = 3.9, 5.9 Hz, 2 H, 4-H), 4.78 ($m_c$, 2 H, 6-H), 7.25-7.27 (m, 2 H, Ar), 7.33-7.35 (m, 4 H, Ph), 7.39-7.40 (m, 4 H, Ph), 7.54 (A part of AA´BB´ system; $J$ = 8.3 Hz, 4 H, Ar), 7.64 (B part of AA´BB´, $J$ = 8.3 Hz, 4 H, Ar), 7.69 (s, 4 H, Ar) ppm; $^{13}C$ NMR (175 MHz, $CD_3OD/CDCl_3$ 5:1): δ 45.2 (d, C-5), 53.7 (t, $NCH_2$), 55.3 (t, 5-$CH_2$), 57.1 (d, C-3), 62.3 (t, 2-$CH_2$), 73.2 (d, C-4), 78.9 (d, C-2), 80.3 (d, C-6), 125.6, 125.6, 126.4, 127.1, 127.7 (5 d, Ph, Ar), 138.6, 138.6, 139.0, 139.1 (4 s, Ar, Ph) ppm; IR (ATR): $\tilde{\nu}$ 3560-3080 (O-H), 3060-3025 (=C-H), 2950-2800 (C-H), 1235 (C-O) $cm^{-1}$; ESI-TOF ($m/z$): $[M + H]^+$ calcd for

$C_{46}H_{53}N_2O_8$, 761.3796; found, 761.3796; $[M + 2H]^{2+}$ calcd for $C_{46}H_{54}N_2O_8$, 381.1934; found, 381.1945.

**(1*S*,2*R*,4*S*,5*R*,8*S*)-6-Benzyl-2-(4-bromophenyl)-4-[(*tert*-butyldimethylsiloxy)methyl]-3-oxa-6-azabicyclo[3.2.1]octan-8-ol (24) and (2*S*,3*R*,4*R*,5*S*,6*R*)-3-(benzylamino)-6-(4-bromophenyl)-2-[(*tert*-butyldimethylsiloxy)methyl]-5-(hydroxymethyl)tetra-hydro-2*H*-pyran-4-ol (25)**

Under an argon atmosphere compound **12a** (150 mg, 0.281 mmol) was dissolved in degassed THF (2 mL), a samarium(II) iodide solution (8.12 mL, 0.1 M in THF, 0.812 mmol) was added dropwise and the solution stirred for 30 min at rt. After completion of the reaction (control by TLC), the mixture was stirred under air for 10 min, sat. aq. potassium sodium tartrate solution (20 mL) was added and the aqueous layer was extracted with ethyl acetate (5 x 80 mL). The combined organic layers were dried with $Na_2SO_4$, filtered and the solvents removed in vacuo. The crude product was purified by column chromatography (silica gel, hexanes/EtOAc 2:1 → 1:1) to yield **24** (115 mg, 79%) and **25** (21 mg, 14%) as colorless solids.

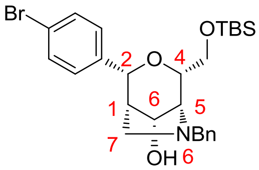


mp 39-41 °C; $[\alpha]_D^{22}$ +22.3 (*c* 1.01, $CHCl_3$); $^1$H NMR (500 MHz, $CDCl_3$): δ 0.06 (s, 6 H, $SiMe_2$), 0.09 (s, 9 H, Si*t*-Bu), 2.20 ($s_{br}$, 1 H, OH), 2.42 (A part of ABX system, $J_{AB}$ = 5.6 Hz, 1 H, 7-H), 2.49 (B part of ABX system, $J_{AB}$ = 5.6 Hz, $J_{BX}$ = 10.0 Hz, 1 H, 1-H), 2.73 (X part of ABX system, $J_{BX}$ = 10.0 Hz, 1 H, 7-H), 3.32 (s, 1 H, 5-H), 3.55 (X part of ABX system, $J_{AX}$ = 5.3 Hz, $J_{BX}$ = 7.7 Hz, 1 H, 4-H), 3.77 (A part of ABX system $J_{AX}$ = 5.3

Hz, $J_{AB}$ = 9.6 Hz, 1 H, 4-$CH_2$), 3.86 (B part of AB system, $J_{BX}$ = 7.7 Hz, $J_{AB}$ = 9.6 Hz, 1 H, 4-$CH_2$), 4.01 (AB system, $J_{AB}$ = 13.4 Hz, 1 H, $NCH_2$), 4.09 (AB system, $J_{AB}$ = 13.4 Hz, 1 H, $NCH_2$), 4.20 (s, 1 H, 8-H), 4.62 (s, 1 H, 2-H), 7.20 (A part of AA´BB´ system, *J* = 8.2 Hz, 2 H, Ar), 7.23-7.24 (m, 1 H, Ph), 7.29-7.32 (m, 2 H, Ph), 7.37-7.38 (m, 2 H, Ph), 7.43 (B part of AA´BB´ system, *J* = 8.5 Hz, 2 H, Ar) ppm; $^{13}$C NMR (125 MHz, $CDCl_3$): δ -5.21, -5.18 (q, SiMe), 18.4, 26.1 (s, q, Si*t*-Bu), 50.0 (t, C-7), 50.2 (d, C-1), 62.0 (t, $NCH_2$), 63.0 (t, 4-$CH_2$), 65.3 (d, C-5), 78.4 (d, C-2), 78.7 (d, C-8), 80.1 (d, C-4), 121.0 (s, Ar), 126.9, 127.7, 128.4, 128.5, 131.3 (5 d, Ph, Ar), 139.9, 140.7 (2 s, Ph, Ar) ppm; IR (ATR): $\tilde{\nu}$ 3570-3150 (O-H), 3090-3030 (=C-H), 2950-2855 (C-H) $cm^{-1}$; ESI-TOF (*m*/*z*): $[M + H]^+$ calcd for $C_{26}H_{37}BrNO_3Si$, 518.1726; found, 518.1738; anal. calcd for $C_{26}H_{36}BrNO_3Si$ (518.6): C, 60.22; H, 7.00; N, 2.70; found: C, 60.42; H, 7.15; N, 2.75.

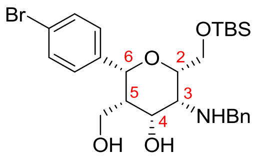


mp 46-48 °C; $[\alpha]_D^{22}$ +56.1 (*c* 1.00, $CH_3OH$); $^{1}$H NMR (700 MHz, $CD_3OD$): δ 0.07 (s, 6 H, $SiMe_2$), 0.88 (s, 9 H, Si*t*-Bu), 2.16 (ddd, *J* = 2.0, 3.6, 9.8 Hz, 1 H, 5-H), 3.14 (dd, *J* = 4.0, 11.5 Hz, 1 H, 5-$CH_2$), 3.23 ($m_c$, 1 H, 3-H), 3.61 (td, *J* = 1.8, 4.7 Hz, 1 H, 2-H), 3.79 (dd, *J* = 2.0, 11.5 Hz, 1 H, 5-$CH_2$), 3.93 (d, *J* = 12.7 Hz, 1 H, $NCH_2$), 3.97 ($m_c$, 2 H, 2-$CH_2$), 4.25 (d, *J* = 12.7 Hz, 1 H, $NCH_2$), 4.41 (dd, *J* = 3.6, 6.1 Hz, 1 H, 4-H), 4.75 (d, *J* = 3.6 Hz, 1 H, 6-H), 7.28-7.31 (m, 1 H, Ph), 7.34-7.36 (m, 2 H, Ph), 7.39-7.40 (m, 4 H, Ph, Ar), 7.48 (d, *J* = 8.5 Hz, 2 H, Ar) ppm; $^{13}$C NMR (175 MHz, $CD_3OD$): δ -5.34, -5.28 (q, SiMe), 19.2, 26.3 (s, q, Si*t*-Bu), 47.4 (d, C-5), 55.6 (t, $NCH_2$), 56.1 (t, 5-$CH_2$), 59.3 (d, C-3), 65.5 (t, 2-$CH_2$), 75.3 (d, C-4), 79.9 (d, C-2), 82.1 (d, C-6), 121.5 (s, Ar), 128.4, 129.3, 129.6, 129.7, 131.9 (5 d, Ph, Ar), 132.1, 140.9 (2 s, Ar, Ph) ppm; IR (ATR): $\tilde{\nu}$ 3555-3080 (O-H,

N-H), 3060-3030 (=C-H), 2930-2855 (C-H) $cm^{-1}$; ESI-TOF (*m/z*): $[M + H]^+$ calcd for $C_{26}H_{39}BrNO_4Si$, 536.1826; found, 536.1847.

***p*-Terphenyl derivative 26**

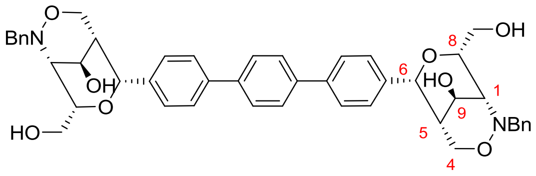


Compound **21** (209 mg, 0.212 mmol) was dissolved in THF (3 mL) and stirred with TBAF (0.86 mL, 1 M in THF, 0.858 mmol) for 2 d at rt. The resulting brownish precipitate was filtered off and washed with dichloromethane (100 mL) and methanol (100 mL) until the solid was colorless. The product was dried in vacuo to yield **23** (48 mg, 30%) as a colorless solid.

mp 247-249 °C; $[\alpha]_D^{22}$ +150.3 (*c* 0.33, $C_5H_5N$); $^1H$ NMR (500 MHz, $C_5D_5N$): δ 2.25 (s, 2 H, 5-H), 3.60 (s, 2 H, 1-H), 3.71 (d, *J* = 11.6 Hz, 2 H, 4-H), 4.32 (td, *J* = 1.0, 5.8 Hz, 2 H, 8-H), 4.53 ($s_{br}$, 2 H, 9-H), 4.54 (dd, *J* = 5.8, 10.9 Hz, 2 H, 8-$CH_2$), 4.63 (dd, *J* = 6.1, 10.9 Hz, 2 H, 8-$CH_2$), 4.67 (d, *J* = 14.6 Hz, 2 H, $NCH_2$), 4.80 (dd, *J* = 2.2, 11.6 Hz, 2 H, 4-H), 5.13 (d, *J* = 14.6 Hz, 2 H, $NCH_2$), 5.17 (s, 2 H, 6-H), 7.27 (C part of AA´´BB´´C system, $J_{CB}$ = 7.4 Hz, 2 H, Ph), 7.36 (A part of AA´´BB´´C system, $J_{AB}$ = 7.4 Hz, 4 H, Ph), 7.67 (B part of AA´´BB´´C system, $J_{ABC}$ = 7.4 Hz, 4 H, Ph), 7.79 (s, 8 H, Ar), 7.81 (s, 4 H, Ar) ppm; $^{13}C$ NMR (125 MHz, $C_5D_5N$): δ 42.8 (d, C-5), 59.5 (t, C-4), 60.4 (t, $NCH_2$), 61.2 (d, C-1), 64.4 (t, 8-$CH_2$), 70.1 (d, C-9), 80.8 (d, C-6), 81.7 (d, C-8), 127.4, 127.6, 128.0, 128.3, 129.0, 129.7 (6 d, Ar, Ph) 140.1, 140.5, 141.1, 141.6 (4 s, Ph, Ar) ppm; IR (ATR): $\tilde{\nu}$ 3500-3275 (O-H), 3030 (=C-H), 2920-2855 (C-H), 1255 (C-O) $cm^{-1}$; ESI-TOF (*m/z*): $[M + H]^+$ calcd for $C_{46}H_{49}N_2O_8$, 757.3489; found, 757.3488;

[M + Na]$^+$ calcd for $C_{46}H_{48}N_2O_8Na$, 779.3308; found, 779.3318; anal. calcd for $C_{46}H_{48}N_2O_8$ (756.9): C, 73.00; H, 6.39; N, 3.70; found: C, 69.73; H, 7.46; N, 3.41.

***p*-Terphenyl derivative 27**

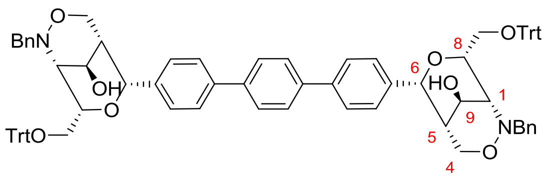


Compound **16** (300 mg, 0.453 mmol), $Pd(PPh_3)_2Cl_2$ (32 mg, 45.3 µmol) and benzene-1,4-diboronic acid (37 mg, 0.222 mmol) were filled in a sealed tube and flushed with argon. DMF (4 mL) and 2 M $Na_2CO_3$ solution (0.9 mL) were added and the mixture was stirred for 3 d at 80 °C. The solution was cooled to rt, brine (20 mL) was added and the aqueous layer extracted with ethyl acetate (80 mL). The combined organic layers were dried with $Na_2SO_4$, filtered and the solvents removed in vacuo. The crude product was purified by column chromatography (silica gel, hexanes/EtOAc 2:1) to yield **27** (140 mg, 51%) as a yellow solid.

mp 144-146 °C; $[\alpha]_D^{22}$ -18.5 (*c* 1.00, $CHCl_3$); $^1$H NMR (700 MHz, $CDCl_3$): δ 2.11 ($s_{br}$, 2 H, 5-H), 3.33 ($s_{br}$, 2 H, 1-H), 3.37 (dd, *J* = 7.8, 8.8 Hz, 2 H, 8-$CH_2$), 3.74 (d, *J* = 12.0 Hz, 2 H, 4-H), 3.81 (d, *J* = 10.5 Hz, 2 H, OH), 3.85-3.87 (m, 2 H, 8-$CH_2$), 3.86, 3.93 (AB system, $J_{AB}$ = 14.9 Hz, 4 H, $NCH_2$), 4.00 (dd, *J* = 5.0, 12.0 Hz, 2 H, 4-H), 4.12-4.16 (m, 4 H, 9-H, 8-H), 4.90 (s, 2 H, 6-H), 7.07-7.08 (m, 4 H, Ph), 7.21-7.28 (m, 8 H, Ph), 7.31-7.33 (m, 10 H, Ph), 7.45 (A part of AA´BB´ system, $J_{AB}$ = 8.3 Hz, 4 H, Ar), 7.50-7.51 (m, 10 H, Ph), 7.60 (B part of AA´BB´ system, $J_{AB}$ = 8.3 Hz, 4 H, Ar), 7.64 (s, 4 H, Ar) ppm; $^{13}$C NMR (175 MHz, $CDCl_3$): δ 41.1 (d, C-5), 61.0 (d, C-1), 61.3 (t, $NCH_2$), 64.3 (t, 8-$CH_2$), 65.3 (t, C-4), 70.5 (d, C-8), 78.8 (d, C-9), 79.7 (d, C-6), 87.1 (s, $CPh_3$), 126.5,

127.0, 127.2, 127.3, 127.5, 128.1, 128.2, 128.4, 128.8 (9 d, Ar, Ph), 138.1, 139.3, 139.8, 139.9, 143.9 (5 s, Ar, Ph) ppm; IR (ATR): $\tilde{\nu}$ 3605-3140 (O-H), 3086-3030 (=C-H), 2955-2850 (C-H); ESI-TOF (*m*/*z*): $[M + H]^+$ calcd for $C_{84}H_{77}N_2O_8$, 1241.5680; found, 1241.5668; $[M + Na]^+$ calcd for $C_{84}H_{76}N_2O_8Na$, 1263.5533; found, 1263.5485; anal. calcd $C_{84}H_{76}N_2O_8$ (1241.5): C, 81.26; H, 6.17; N, 2.26; found: C, 81.17; H, 6.26; N, 2.28.

**_p_-Terphenyl derivatives 30 and 31:**

A suspension of Pd/C (10% Pd, 200 mg) and iPrOH (3 mL) was saturated with hydrogen for 15 min. Compound **27** (100 mg, 80.5 µmol) and TFA (23 mg, 0.02 mL, 0.201 mmol) were dissolved in hexafluoro-2-propanol (2 mL) and added to the suspension. The mixture was stirred for 8 h under hydrogen pressure (balloon), then filtered through Celite® and the solvents were removed in vacuo. Under an argon atmosphere the crude product was dissolved in degassed MeOH (1 mL) and a samarium(II) iodide solution (0.1 M in THF, 4.83 mL, 0.483 mmol) was added dropwise. The mixture was stirred for 30 min at rt and then for another 10 min in the presence of air. A size exclusion chromatography (Sephadex™ LH-20, $CH_3OH$) of the mixture and subsequent purification by thin-layer chromatography (silica gel, $CH_2Cl_2$:MeOH, 7N $NH_3$) afforded **30** (25 mg, 54%) and **31** (20 mg, 37%) as a yellow solid.

**_p_-Terphenyl derivatives 30**

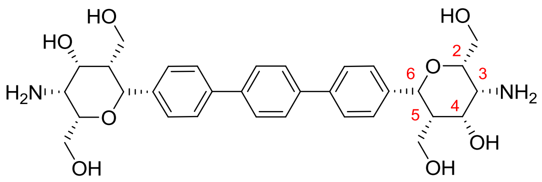

Decomposition >200 °C; $[\alpha]_D^{22}$ +13.5 (*c* 0.40, $CH_3OH$); $^1$H NMR (700 MHz, $CD_3OD$): δ 2.33 ($m_c$, 2 H, 5-H), 3.28 (dd, *J* = 2.8, 11.0 Hz, 2 H, 5-$CH_2$), 3.66 (dd, *J* = 1.6, 4.6 Hz, 2 H, 3-H), 3.80 (dd, *J* = 1.5, 11.0 Hz, 2 H, 5-$CH_2$), 3.85 (X part of ABX system with additional *J* = 1.6 Hz, $J_{AX}$ = $J_{BX}$ = 4.7 Hz, 2 H, 2-H), 3.95 (A part of ABX system, $J_{AX}$ = 4.7, $J_{AB}$ = 12.0 Hz, 2 H, 2-$CH_2$), 3.98 (B part ABX system, $J_{BX}$ = 4.7, $J_{AB}$ = 12.0 Hz, 2 H, 2-$CH_2$), 4.46 ($s_{br}$, 2 H, NH), 4.59 (dd, *J* = 4.6, 6.6 Hz, 2 H, 4-H), 4.94 (d, *J* = 3.6 Hz, 2 H, 6-H), 7.58 (A part of AA´BB´ system, $J_{AB}$ = 8.2 Hz, 4 H, Ar), 7.69 (B part of AA´BB´ system, $J_{AB}$ = 8.2 Hz, 4 H, Ar), 7.73 (s, 4 H, Ar) ppm; $^{13}$C NMR (175 MHz, $CD_3OD$): δ 46.0 (d, C-5), 52.0 (d, C-3), 55.2 (t, 5-$CH_2$), 63.4 (t, 2-$CH_2$), 69.1 (d, C-4), 77.1 (d, C-2), 82.0 (d, C-6), 127.5, 127.6, 128.3 (3 d, Ar), 139.9, 140.7, 141.0 (3 s, Ar) ppm; IR (ATR): $\tilde{\nu}$ 3570-3475 (O-H, N-H), 3095-3020 (=C-H), 2960-2930 (C-H), 1240 (C-O) $cm^{-1}$; ESI-TOF (*m*/*z*): $[M + H]^+$ calcd for $C_{32}H_{41}N_2O_8$, 581.2863; found, 581.2882; [M + 2H] calcd for $C_{32}H_{42}N_2O_8$, 291.1471; found 291.1475.

***p*-Terphenyl derivatives 31**

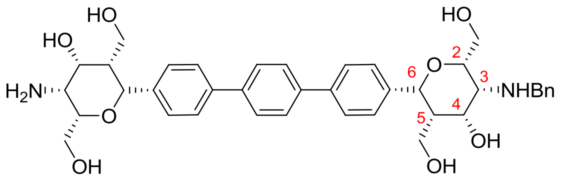


mp 154-156 °C; $[\alpha]_D^{22}$ +100.9 (*c* 1.00, $CH_3OH$); $^1$H NMR (700 MHz, $CD_3OD$): δ 2.32 ($s_{br}$, 1 H, 5-H), 2.37 ($s_{br}$, 1 H, 5´-H), 3.28-3.29 (m, 2 H, 5-$CH_2$, 5´-$CH_2$), 3.62 ($s_{br}$, 1 H, 3-H), 3.69-3.71 (m, 2 H, 2-H, 3´-H), 3.80 (d, *J* = 10.4 Hz, 1 H, 5-$CH_2$), 3.84 ($m_c$, 1 H, 2´-H), 3.88 (dd, *J* = 2.2, 10.8 Hz, 1 H, 5´-$CH_2$), 3.94 (dd, *J* = 4.8, 11.8 Hz, 1 H, 2´-$CH_2$), 3.97-4.00 (m, 2 H, 2-$CH_2$, 2´-$CH_2$), 4.07 (dd, *J* = 3.3, 12.2 Hz, 1 H, 2-$CH_2$), 4.33 (d, *J* = 12.7 Hz, 1 H, $NCH_2$), 4.57 (d, $m_c$, *J* = 12.7 Hz, 2 H, $NCH_2$, 4-H), 4.67 (dd, *J* = 4.1, 6.7 Hz, 1 H,

4´-H), 4.93 (d, *J* = 3.3 Hz, 1 H, 6-H), 4.96 (d, *J* = 3.9 Hz, 1 H, 6´-H), 7.41-7.46 (m, 3 H, Ph), 7.49-7.50 (m, 2 H, Ph), 7.56-7.59 (m, 4 H, Ar), 7.68-7.70 (m, 4 H, Ar), 7.74 (s, 4 H, Ar); $^{13}C$ NMR (175 MHz, $CD_3OD$): δ 46.1 (d, C-5), 46.5 (d, C-5´), 51.9 (d, C-3), 53.9 (t, $NCH_2$), 55.3 (t, 5-$CH_2$), 55.9 (t, 5´-$CH_2$), 59.8 (d, C-3´), 63.4 (t, 2´-$CH_2$), 65.1 (t, 2-$CH_2$), 69.5 (d, C-4), 72.3 (d, C-4´), 77.3 (d, C-2), 77.4 (d, C-2´), 81.9 (d, C-6), 82.4 (d, C-6´), 127.5, 127.6, 127.7, 128.3, 130.0,130.2, 130.4 (7 d, Ar, Ph), 139.9, 140.0, 140.7, 141.0 (4 s, Ph, Ar); IR (ATR): $\tilde{\nu}$ 3605-3430 (O-H), 3090-3030 (=C-H), 2950-2880 (C-H), 1255 (C-O) $cm^{-1}$; ESI-TOF (*m*/*z*): $[M + H]^+$ calcd for $C_{39}H_{47}N_2O_8$, 671.3327; found, 671.3357; $[M + 2H]^{2+}$ calcd for $C_{78}H_{93}N_4O_{16}$, 336.1706; found 336.1713.

## References


1. Bouché, L.; Kandziora, M.; Reissig, H.-U., *Beilstein J. Org. Chem.* **2014,** *10*, 213–223. doi: 10.3762/bjoc.10.17
2. Helms, M.; Schade, W.; Pulz, R.; Watanabe, T.; Al-Harrasi, A.; Fišera, L.; Hlobilová, I.; Zahn, G.; Reissig, H.-U. *Eur. J. Org. Chem.* **2005**, 1003–1019. doi: 10.1002/ejoc.200400627
3. Borch, R. F.; Bernstein, M. D.; Durst, H. D. *J. Am. Chem. Soc.* **1971**, *93*, 2897–2904. doi: 10.1021/ja00741a013
4. Imamoto, T.; Ono, M.; *Chem. Lett.* **1987**, *16*, 501–502. doi: 10.1246/cl.1987.501
5. Pfrengle, F.; Dekaris, V.; Schefzig, L.; Zimmer, R.; Reissig, H.-U., *Synlett* **2008**, 2965–2968. doi: 10.1002/chem.201001060
