# Supplementary material for: Synthesis of rigid p-terphenyl-linked carbohydrate mimetics
Source: Beilstein J Org Chem. 2014 Jul 30;10:1749–58. doi: 10.3762/bjoc.10.182 (PMC4142845; doi:10.3762/bjoc.10.182)
Supplement: File 2 — Characterization data 1H NMR and 13C NMR spectra of synthesized compounds. [file Beilstein_J_Org_Chem-10-1749-s002.pdf]

Supporting Information File 2

for

Synthesis of rigid *p*-terphenyl-linked carbohydrate mimetics

Maja Kandziora and Hans-Ulrich Reissig*

Address: Freie Universität Berlin, Institut für Chemie und Biochemie, Takustraße 3, D-14195 Berlin, Germany

Email: Hans-Ulrich Reissig - hans.reissig@chemie.fu-berlin.de

* Corresponding author

author

Characterization data ^{1}H NMR and ^{13}C NMR spectra of synthesized compounds

Table of contents:

- NMR spectra of the diol s2

- NMR spectra of (*Z*)-nitrones and 1,2 oxazines s3

- NMR spectra of bicyclic 1,2-oxazines and pyranes s7

- NMR spectra of dimers s18

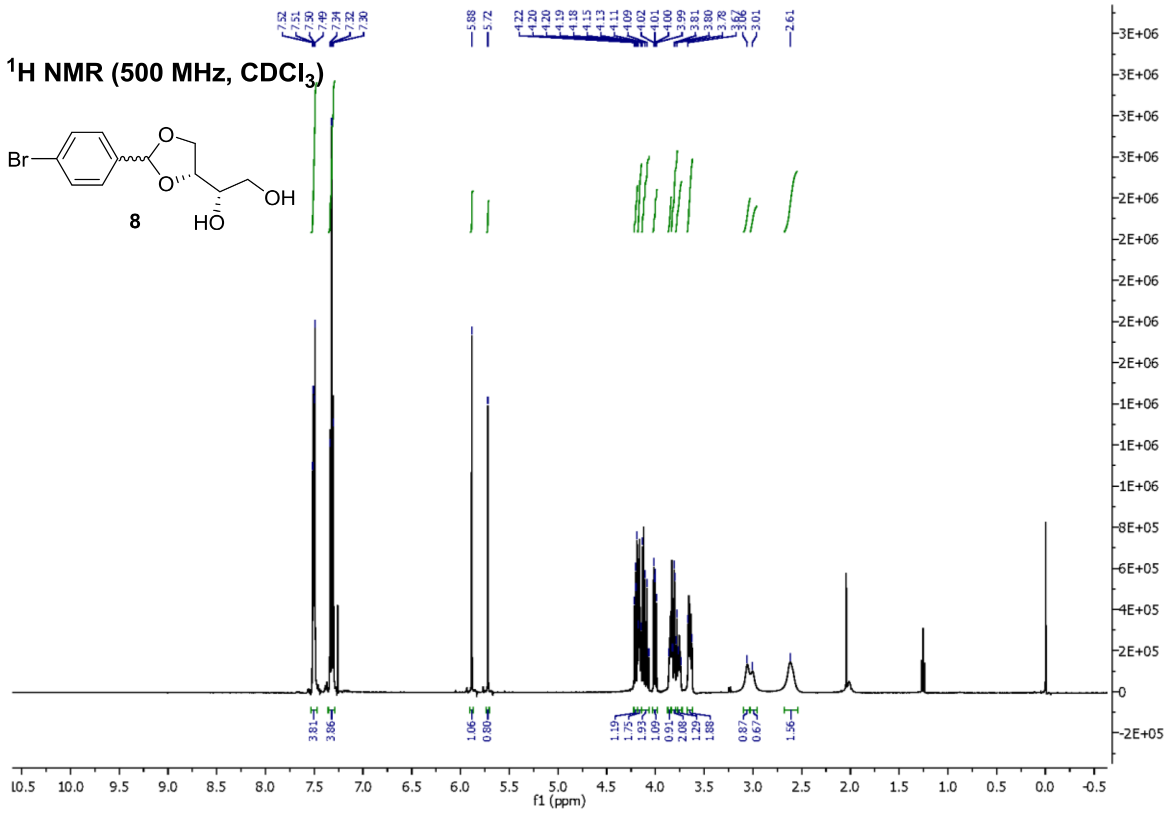
^{1}H NMR (500 MHz, $CDCl_3$)
Br
O
O
OH
HO
8
f1 (ppm)

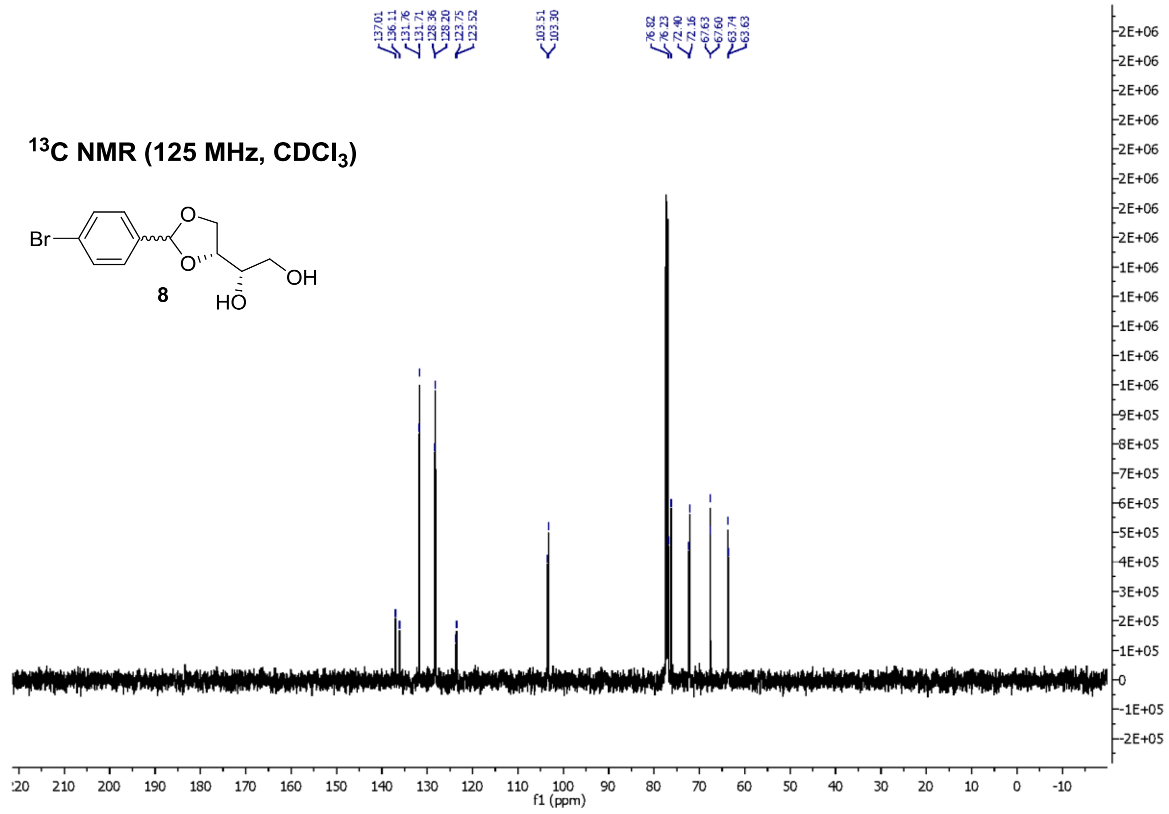
^{13}C NMR (125 MHz, $CDCl_3$)
Br
O
O
OH
HO
8
f1 (ppm)

^{1}H NMR (500 MHz, $CDCl_3$)

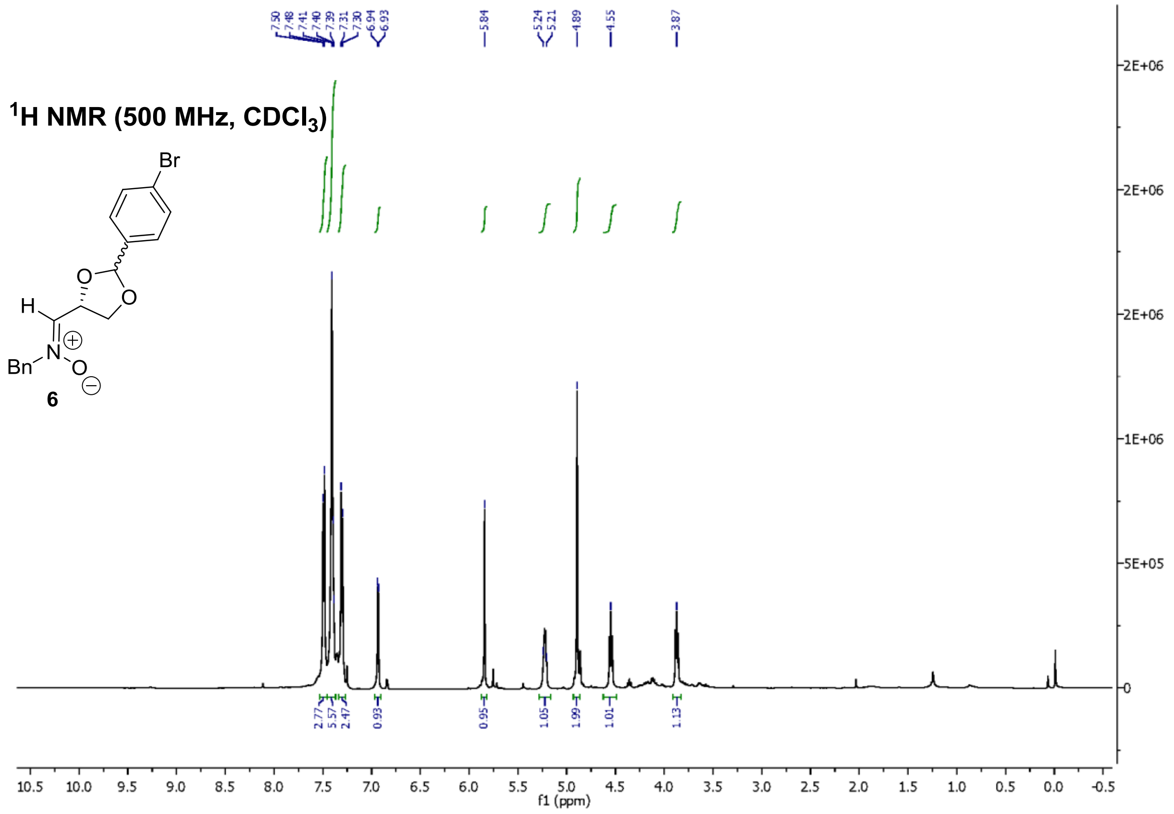

^{13}C NMR (125 MHz, $CDCl_3$)

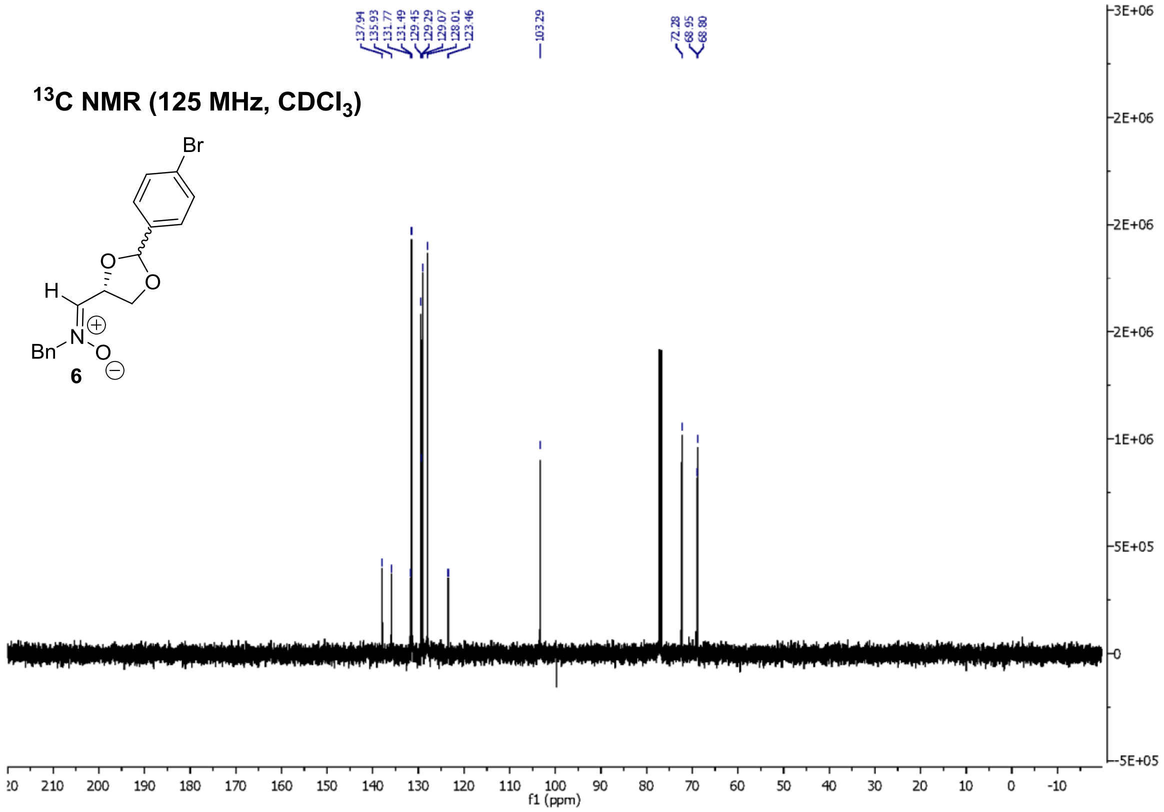

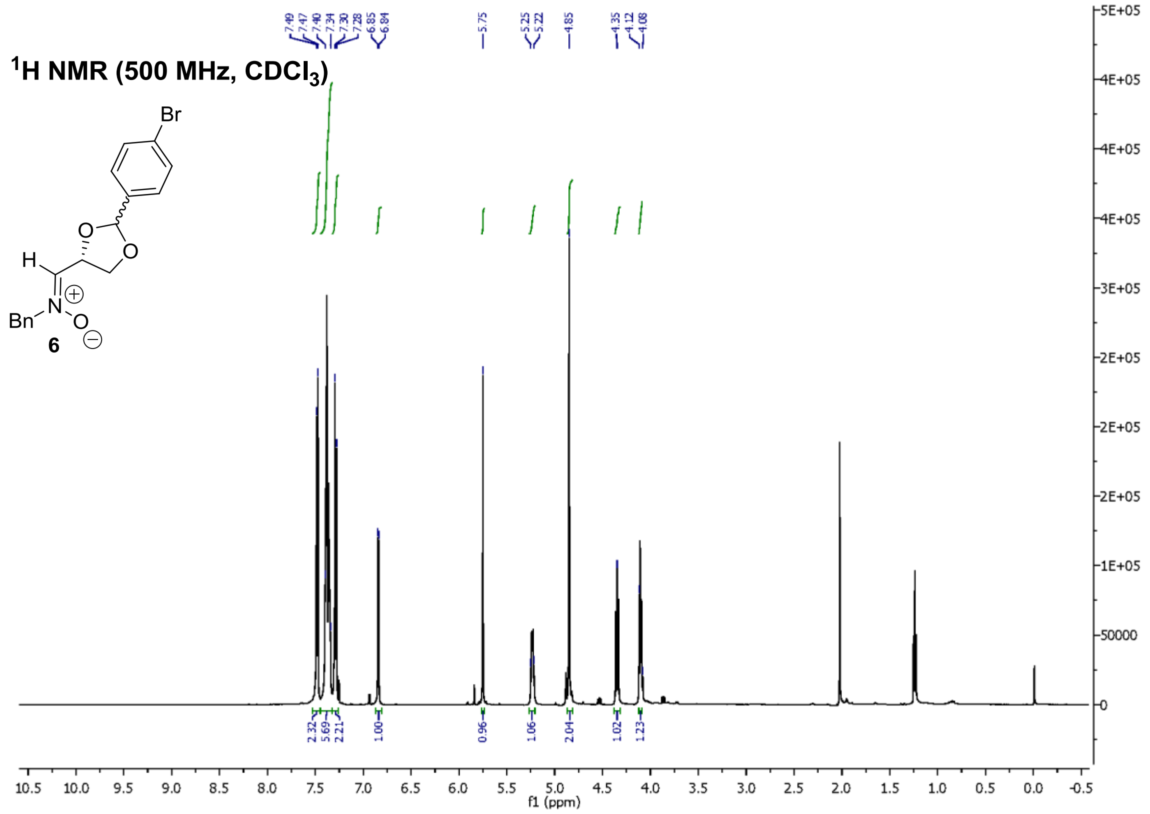

1H NMR (500 MHz, CDCl3)
Br
O
O
H
N
Bn
O
6
f1 (ppm)

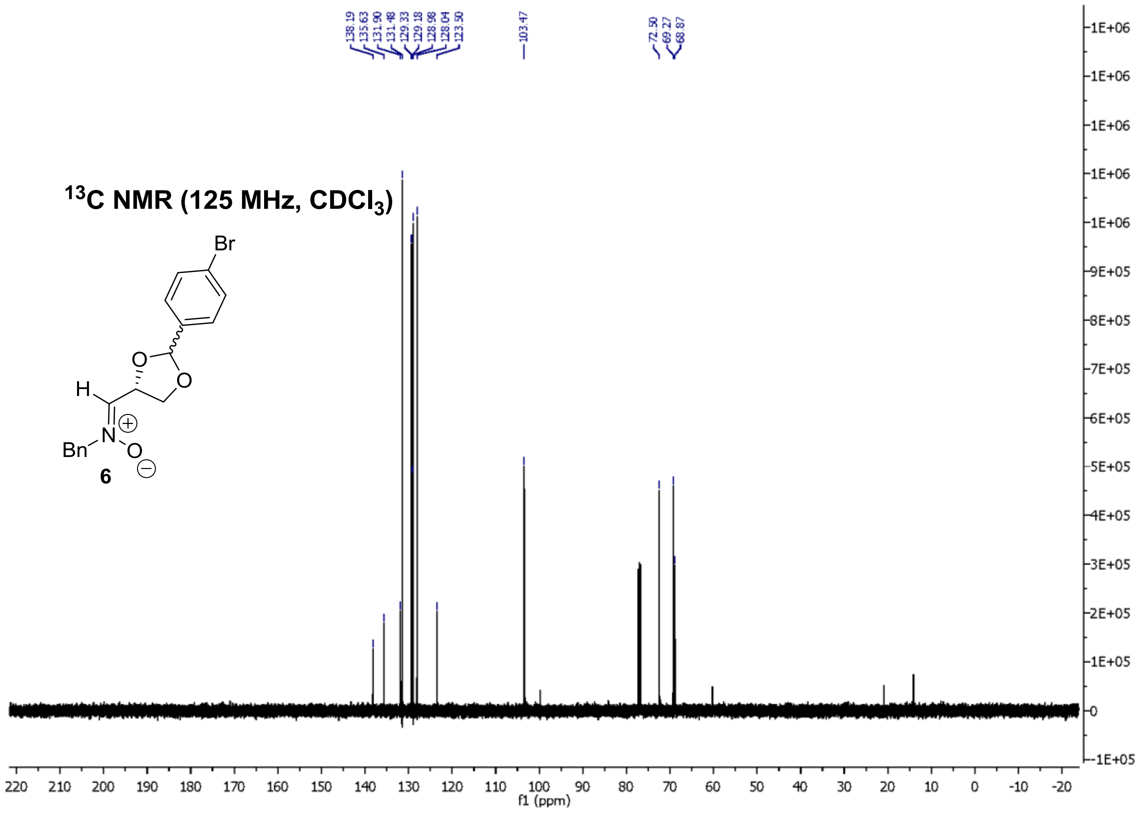

13C NMR (125 MHz, CDCl3)
Br
O
O
H
N
Bn
O
6
f1 (ppm)

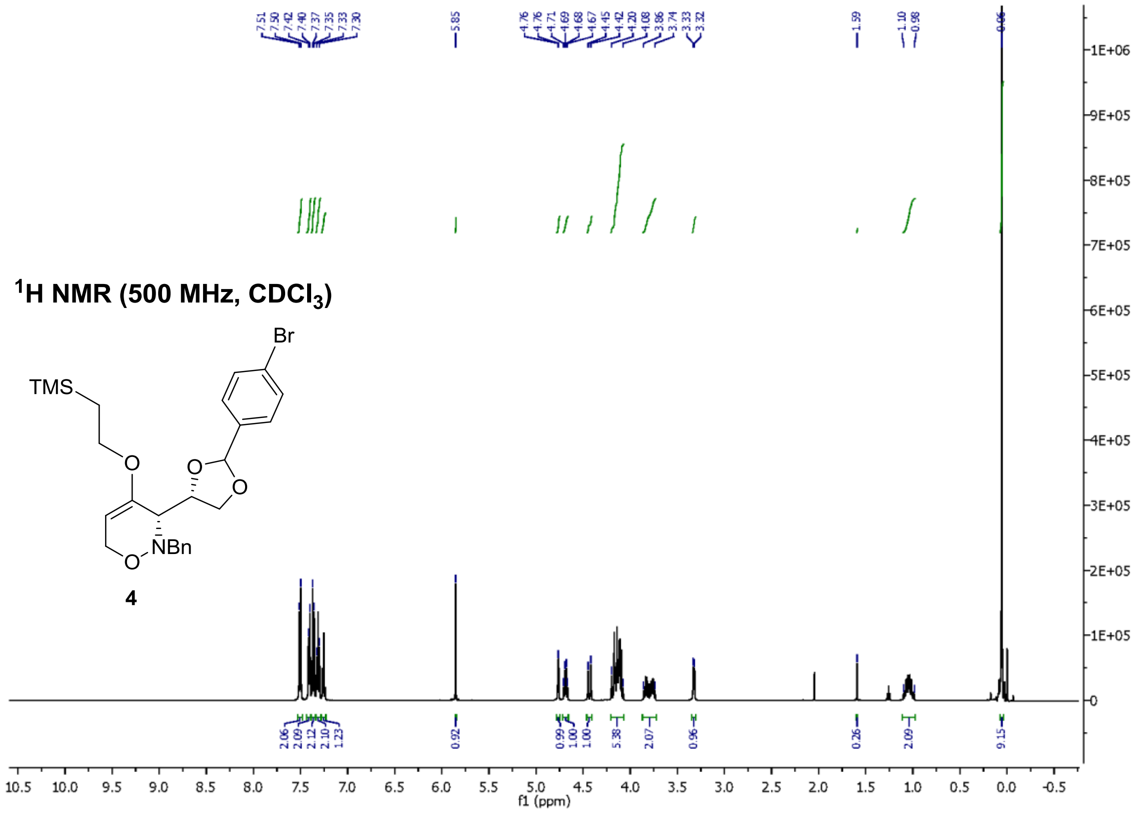

1H NMR (500 MHz, CDCl3)
Br
TMS
O
NBn
4
f1 (ppm)

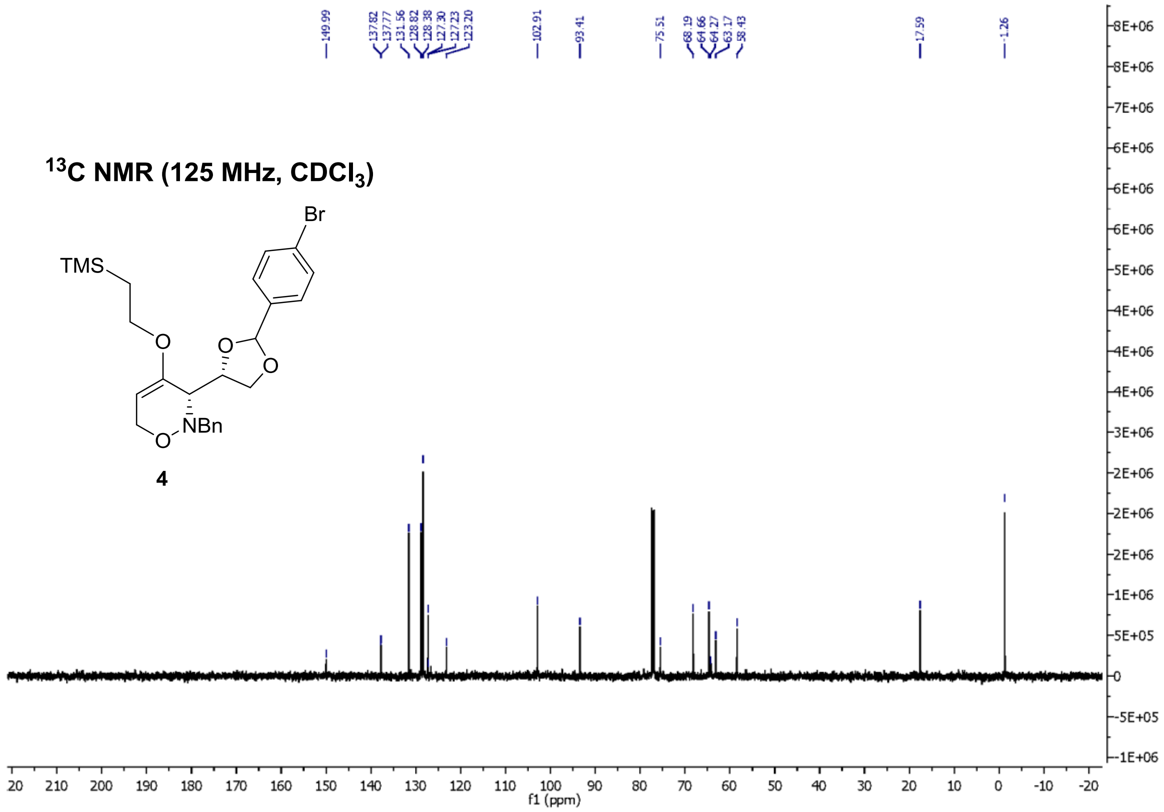

13C NMR (125 MHz, CDCl3)
Br
TMS
O
NBn
4
f1 (ppm)

^{1}H NMR (500 MHz, CDCl$_3$)

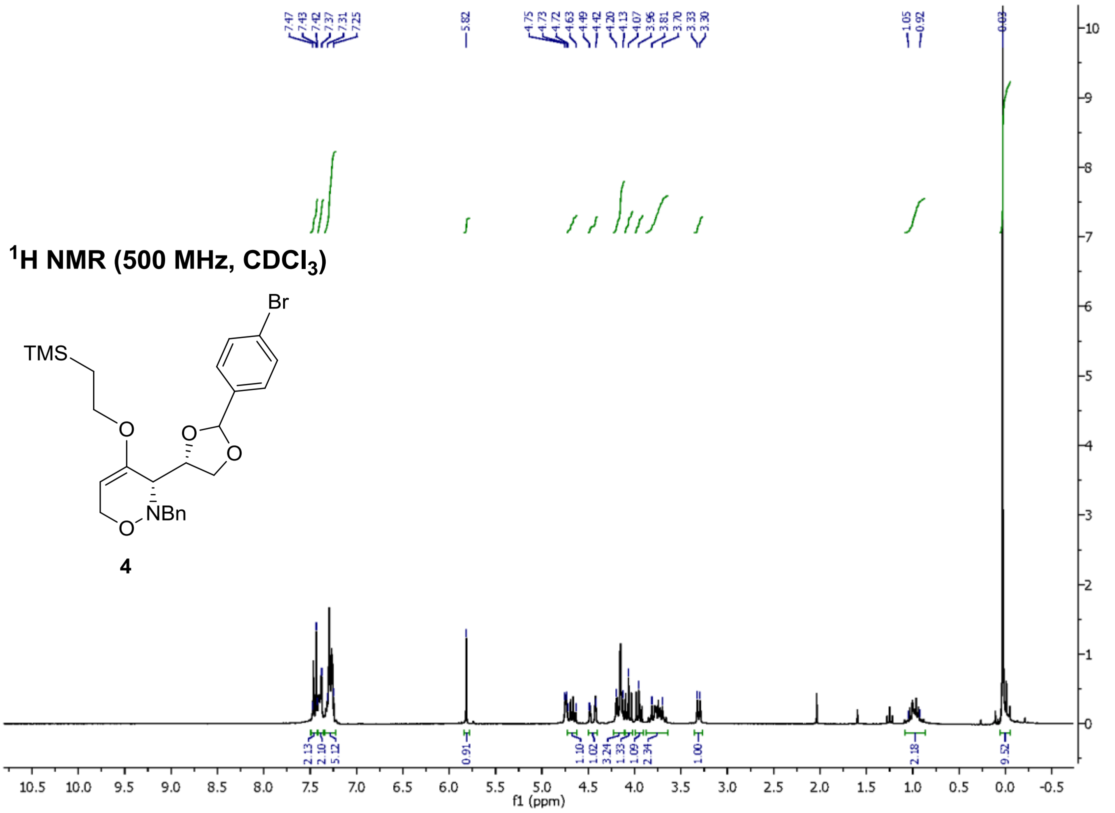

4

^{13}C NMR (125 MHz, CDCl$_3$)

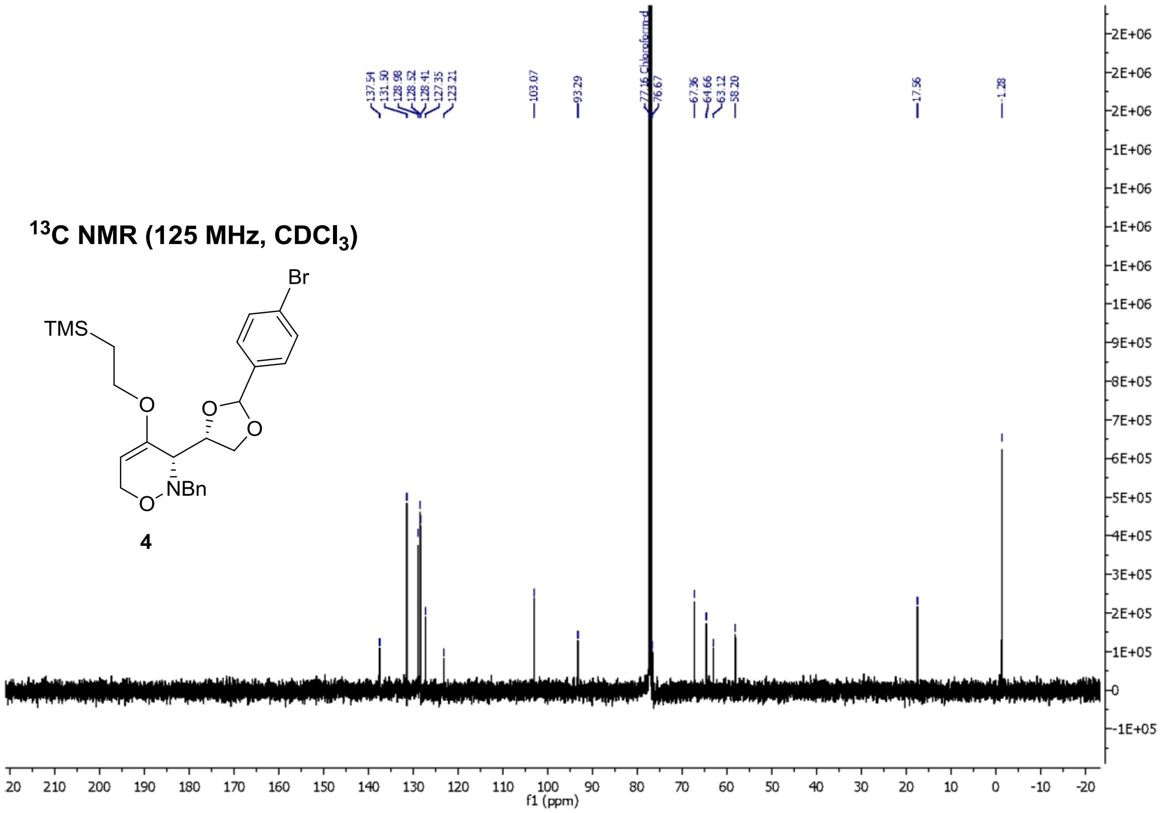

4

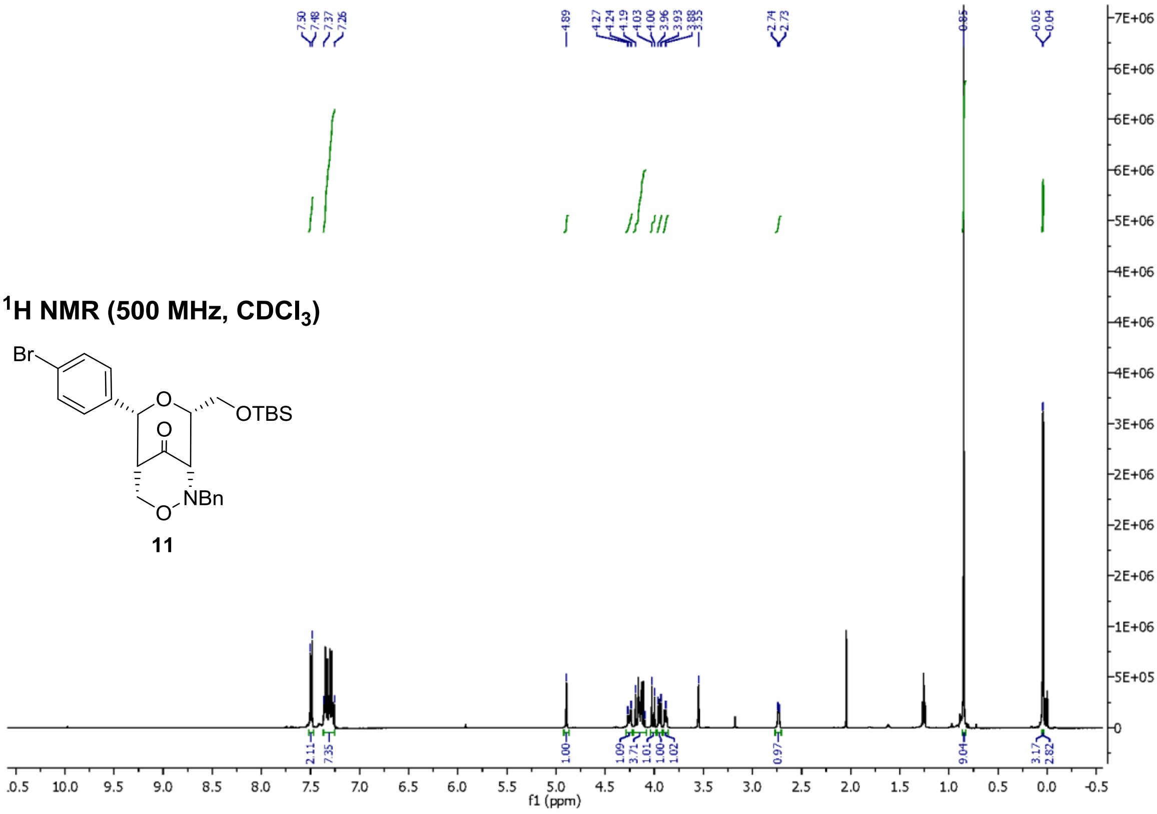

^{1}H NMR (500 MHz, CDCl$_3$)

11

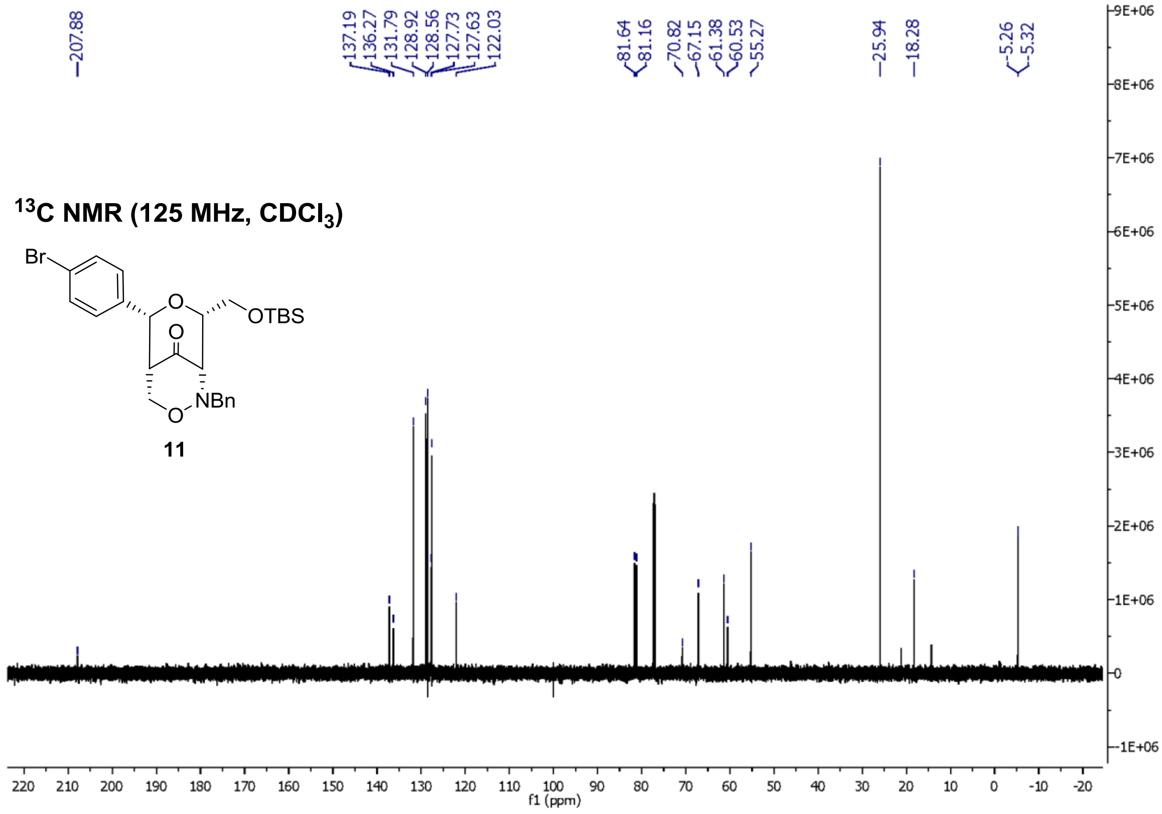

^{13}C NMR (125 MHz, CDCl$_3$)

11

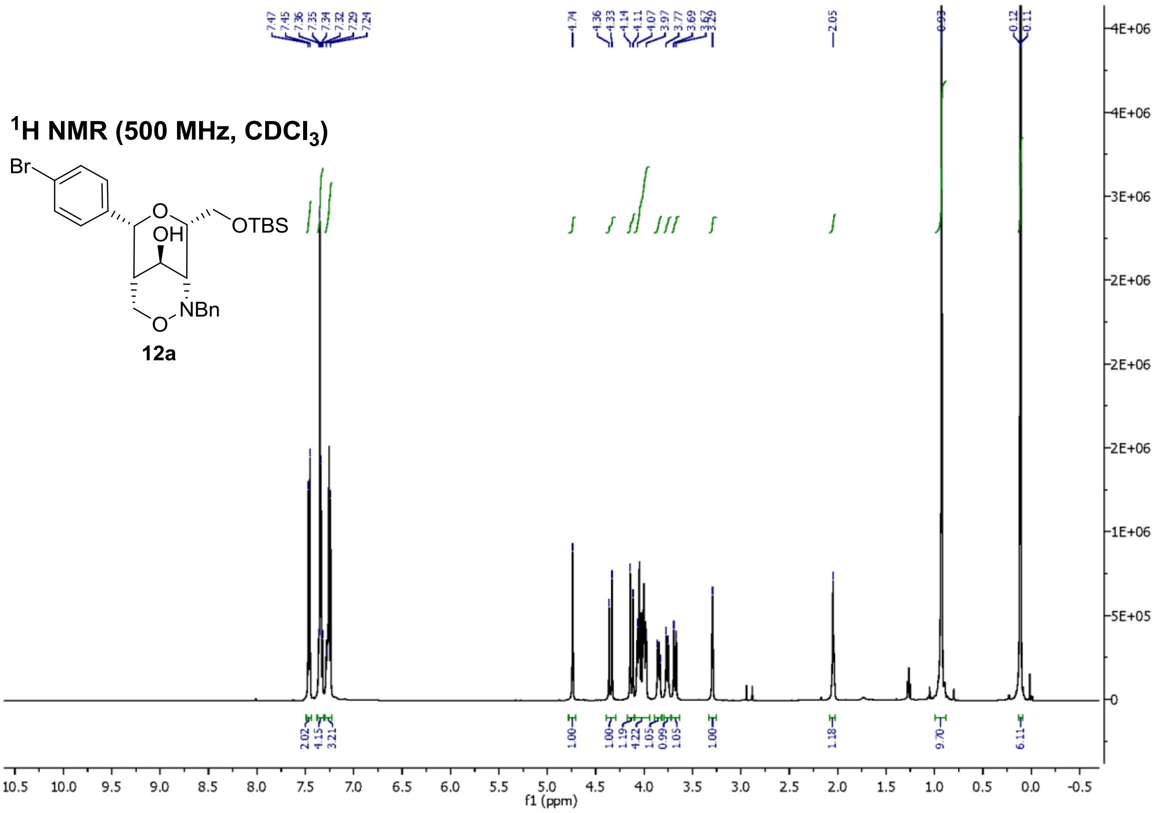

1H NMR (500 MHz, CDCl3)
Br
O
OH
OTBS
NBn
O
12a
7.47 7.45 7.36 7.35 7.34 7.32 7.29 7.24
4.74 4.36 4.33 4.14 4.11 4.07 3.97 3.77 3.69 3.67 3.29
2.05
0.93
0.12 0.11
2.02 4.15 3.21
1.00 1.00 1.19 4.22 1.05 0.99 1.05 1.00
1.18
9.70
6.11
f1 (ppm)

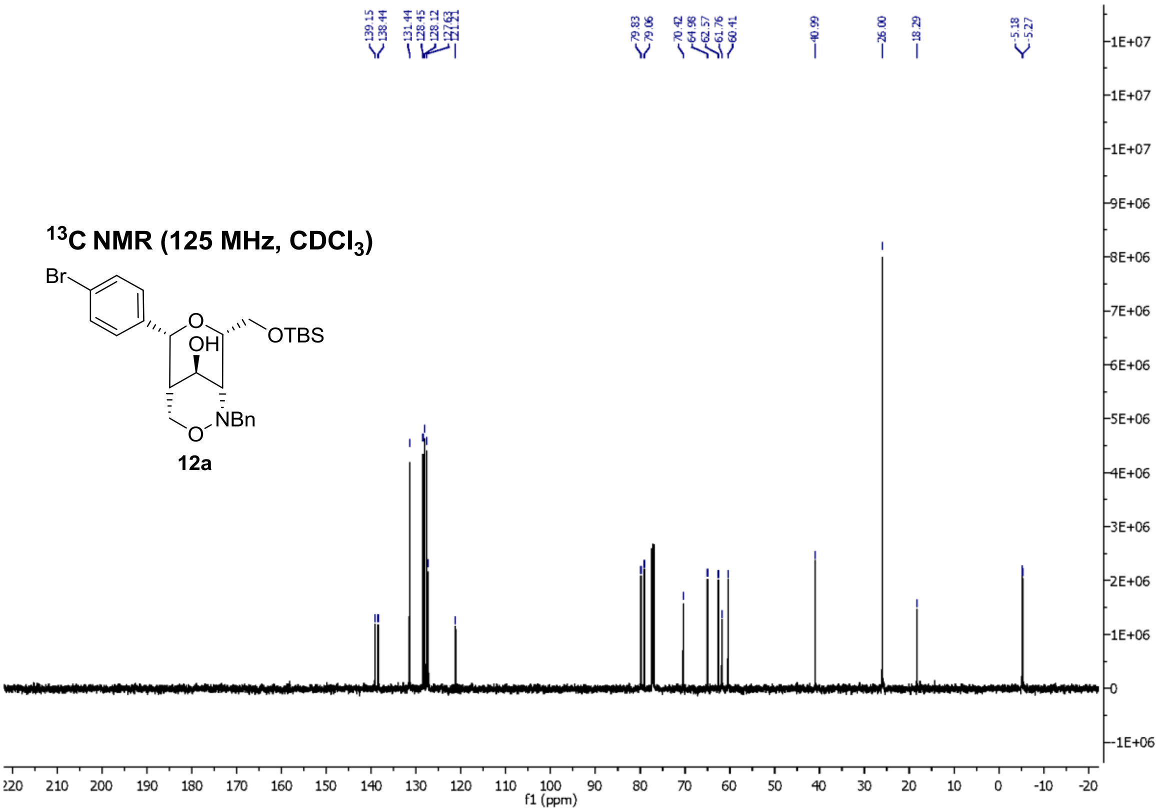

13C NMR (125 MHz, CDCl3)
Br
O
OH
OTBS
NBn
O
12a
139.15 138.44
131.44 128.45 128.12 127.63 121.21
79.83 79.06
70.42 64.98 62.57 61.76 60.41
40.99
26.00
18.29
-5.18 -5.27
f1 (ppm)

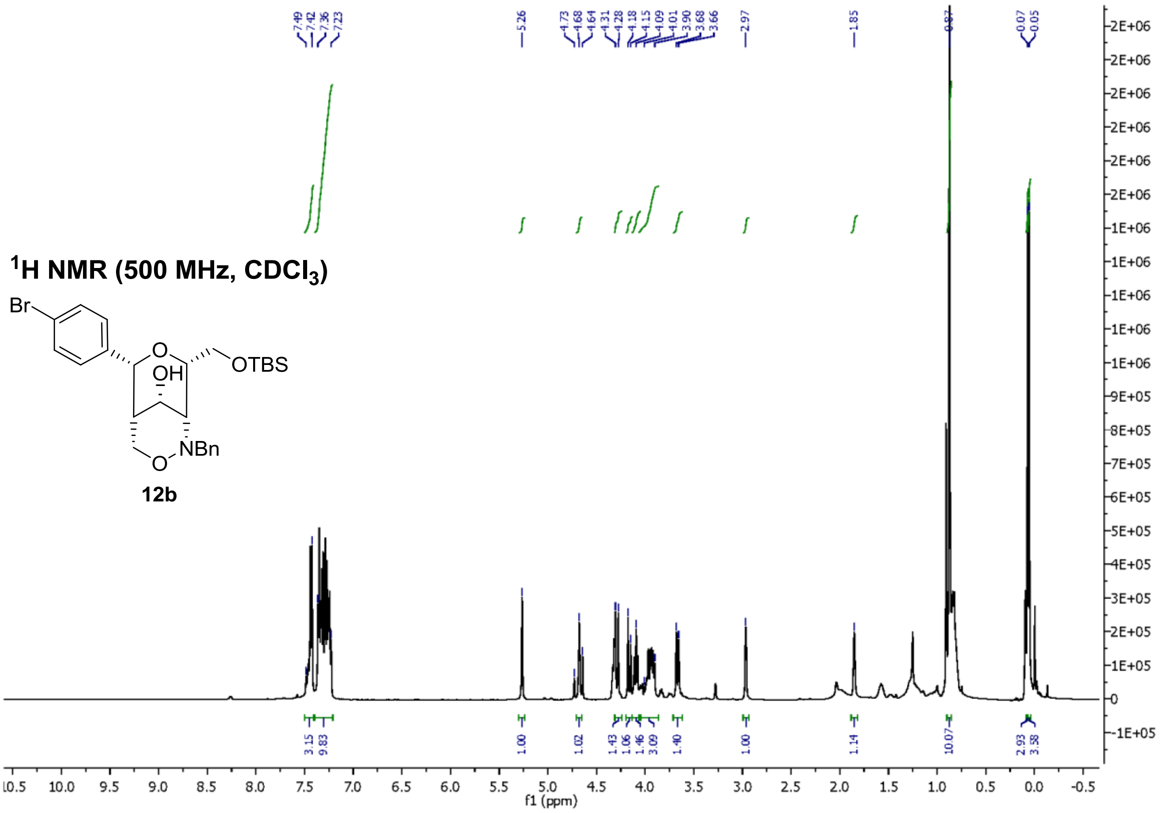

1H NMR (500 MHz, CDCl3)
Br
O
OH
OTBS
NBn
O
12b
f1 (ppm)

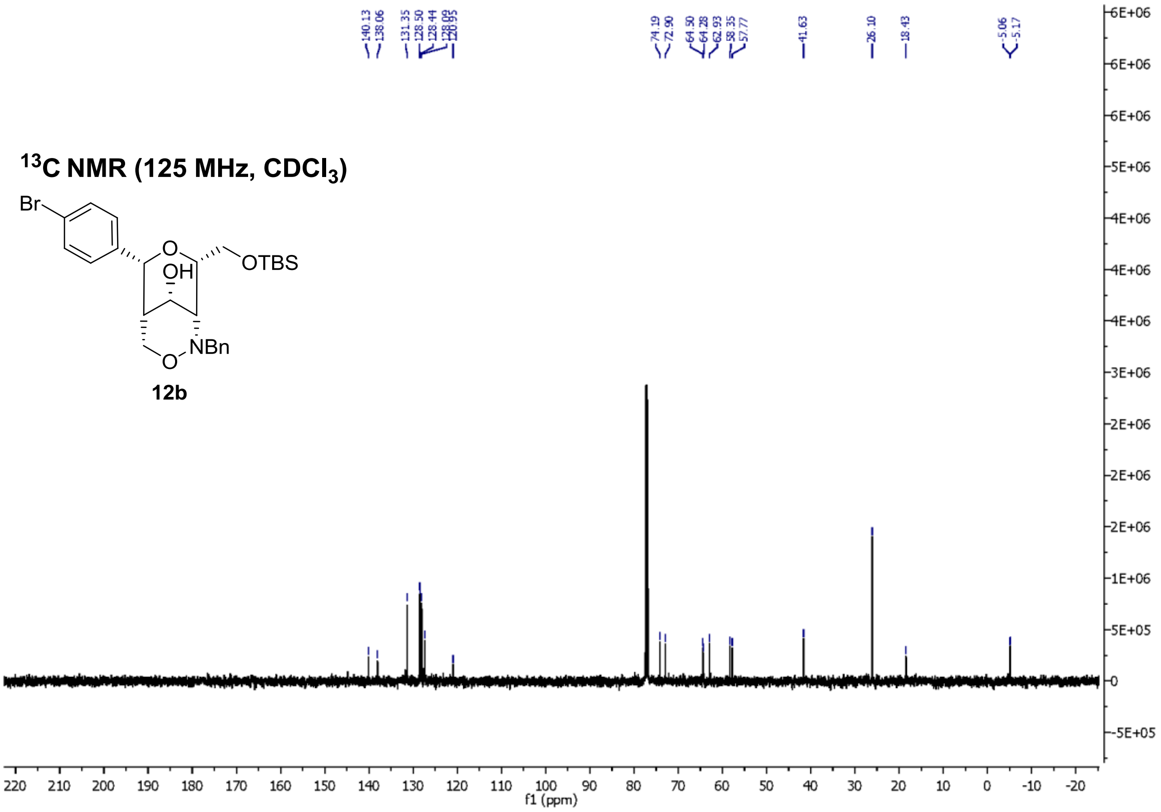

13C NMR (125 MHz, CDCl3)
Br
O
OH
OTBS
NBn
O
12b
f1 (ppm)

^{1}H NMR (500 MHz, $CDCl_3$)

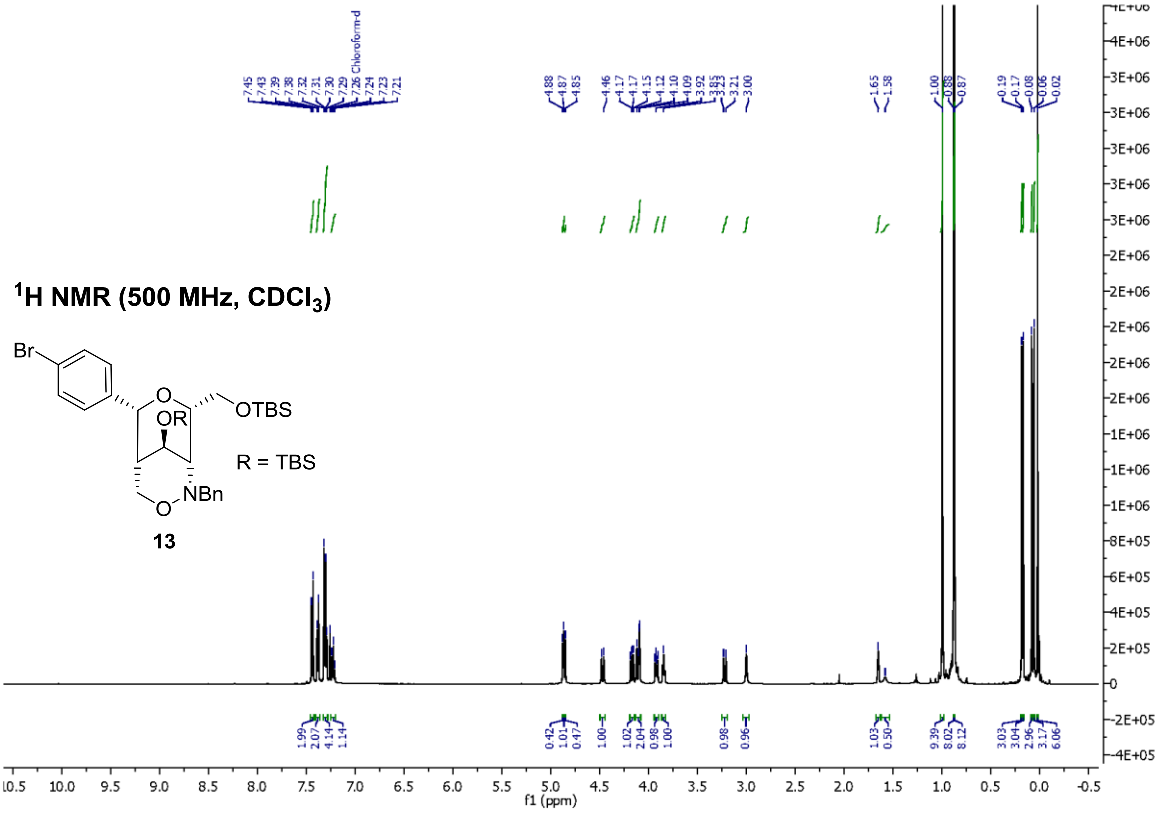

13

^{13}C NMR (125 MHz, $CDCl_3$)

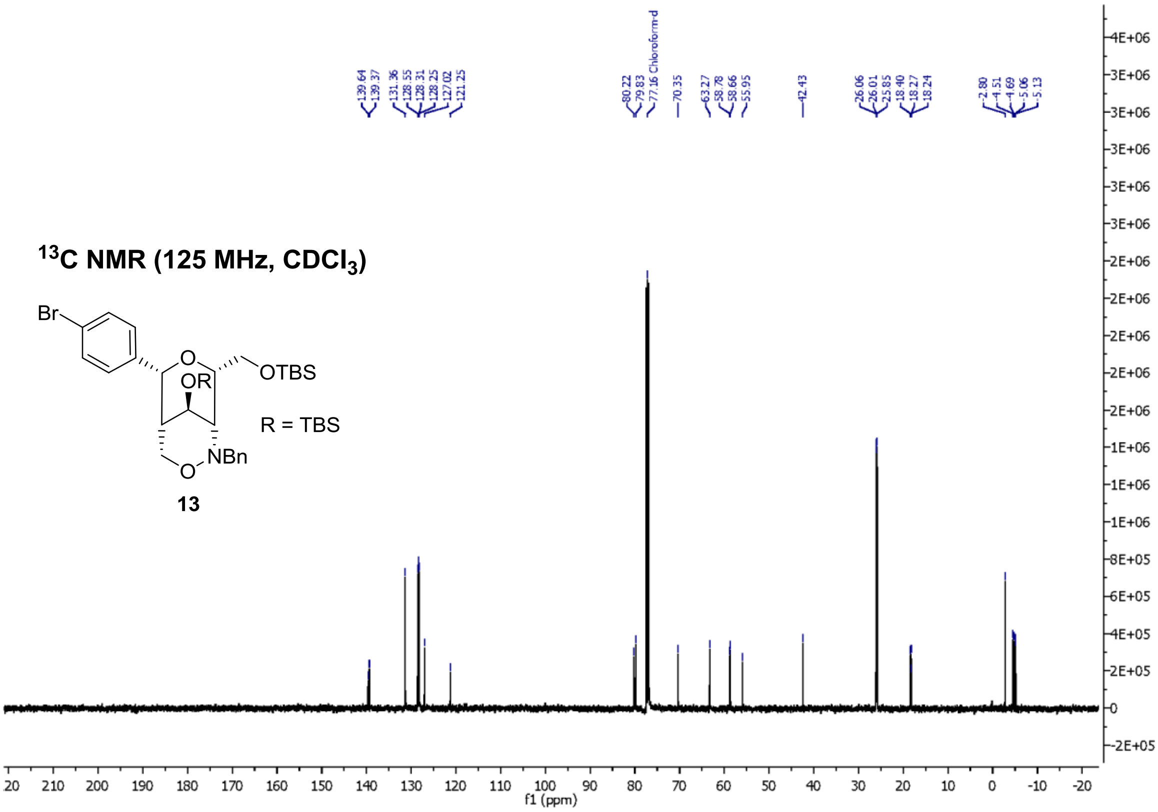

13

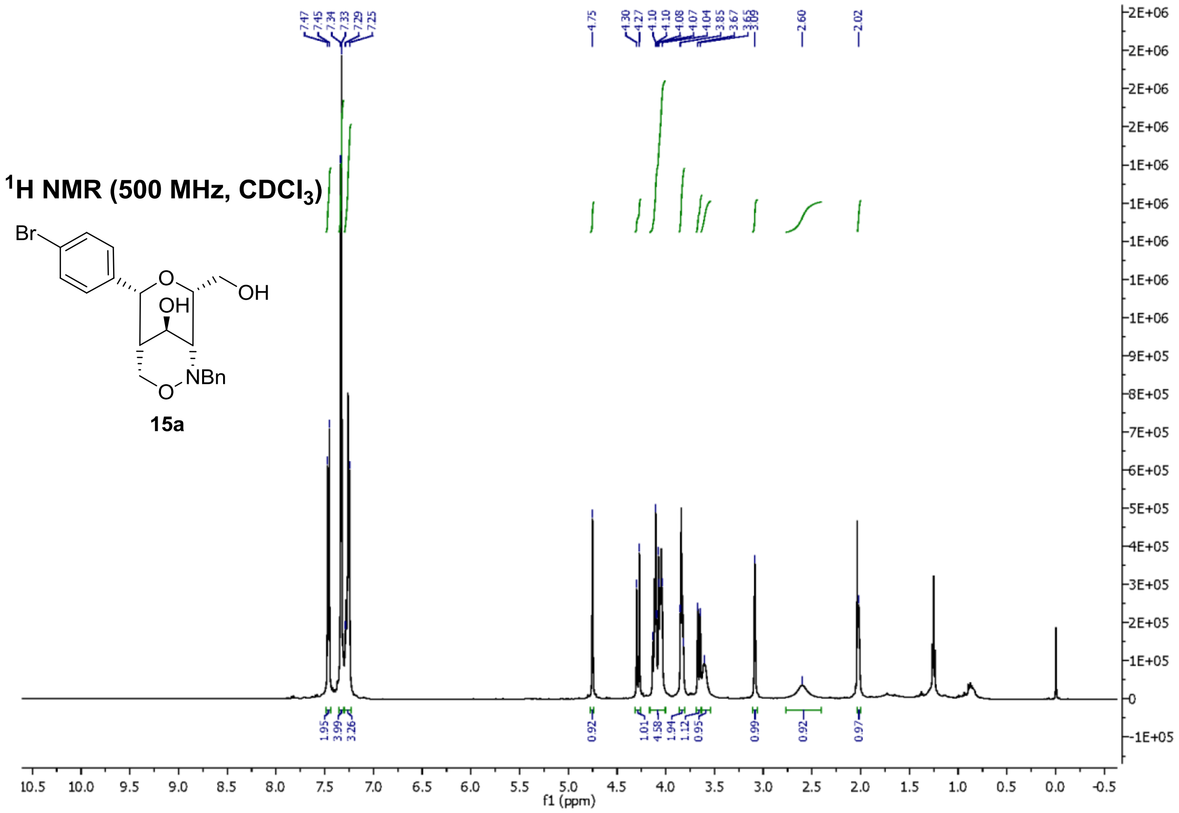
^{1}H NMR (500 MHz, CDCl$_3$)
Br
O
OH
OH
NBn
O
15a
7.47
7.45
7.34
7.33
7.29
7.25
4.75
4.30
4.27
4.10
4.10
4.08
4.07
4.04
3.85
3.67
3.65
3.09
2.60
2.02
1.95
3.99
3.26
0.92
1.01
4.58
1.94
1.12
0.95
0.99
0.92
0.97
f1 (ppm)

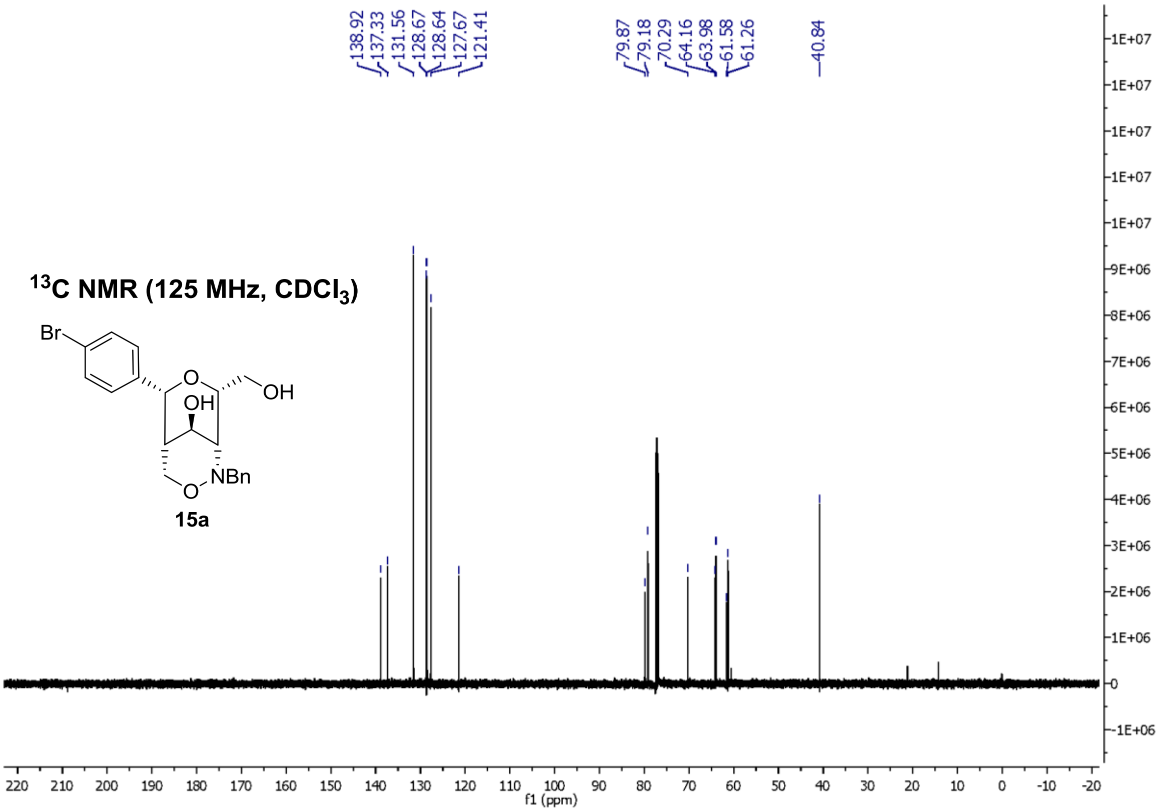
^{13}C NMR (125 MHz, CDCl$_3$)
Br
O
OH
OH
NBn
O
15a
138.92
137.33
131.56
128.67
128.64
127.67
121.41
79.87
79.18
70.29
64.16
63.98
61.58
61.26
40.84
f1 (ppm)

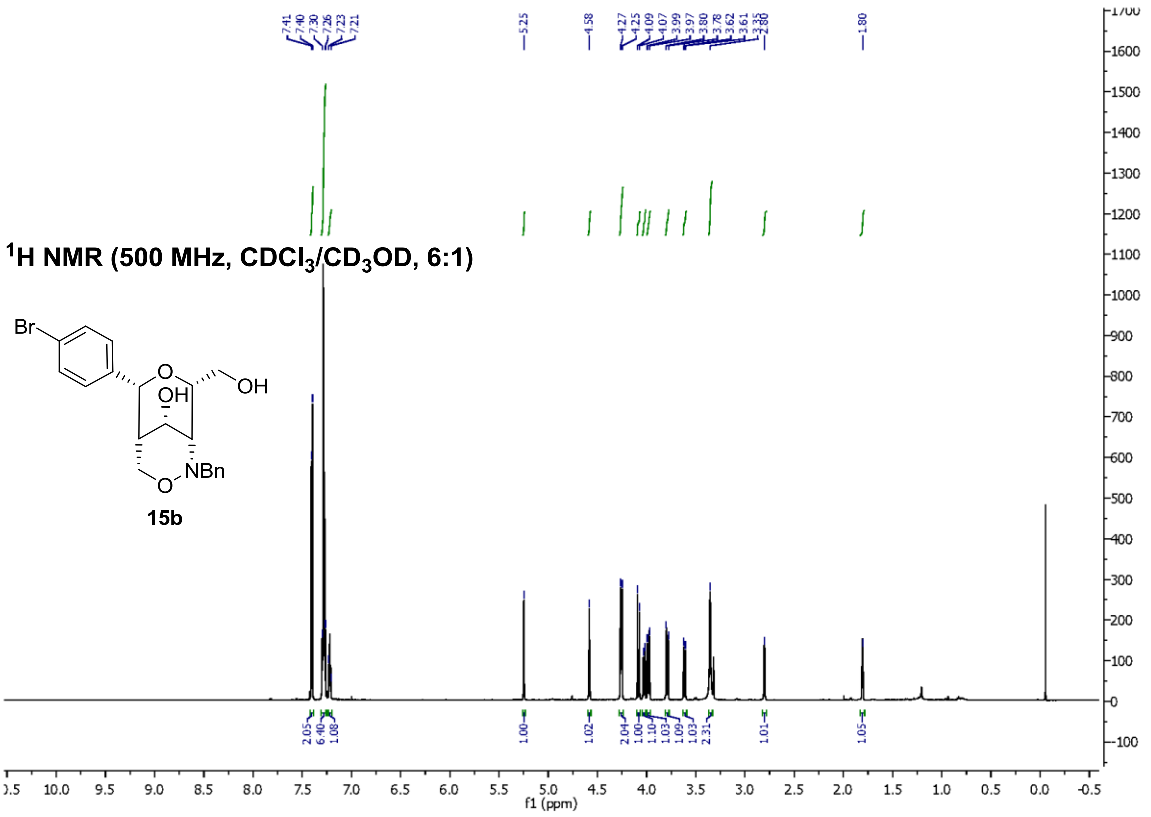
1H NMR (500 MHz, CDCl3/CD3OD, 6:1)
Br
O
OH
OH
NBn
O
15b
f1 (ppm)

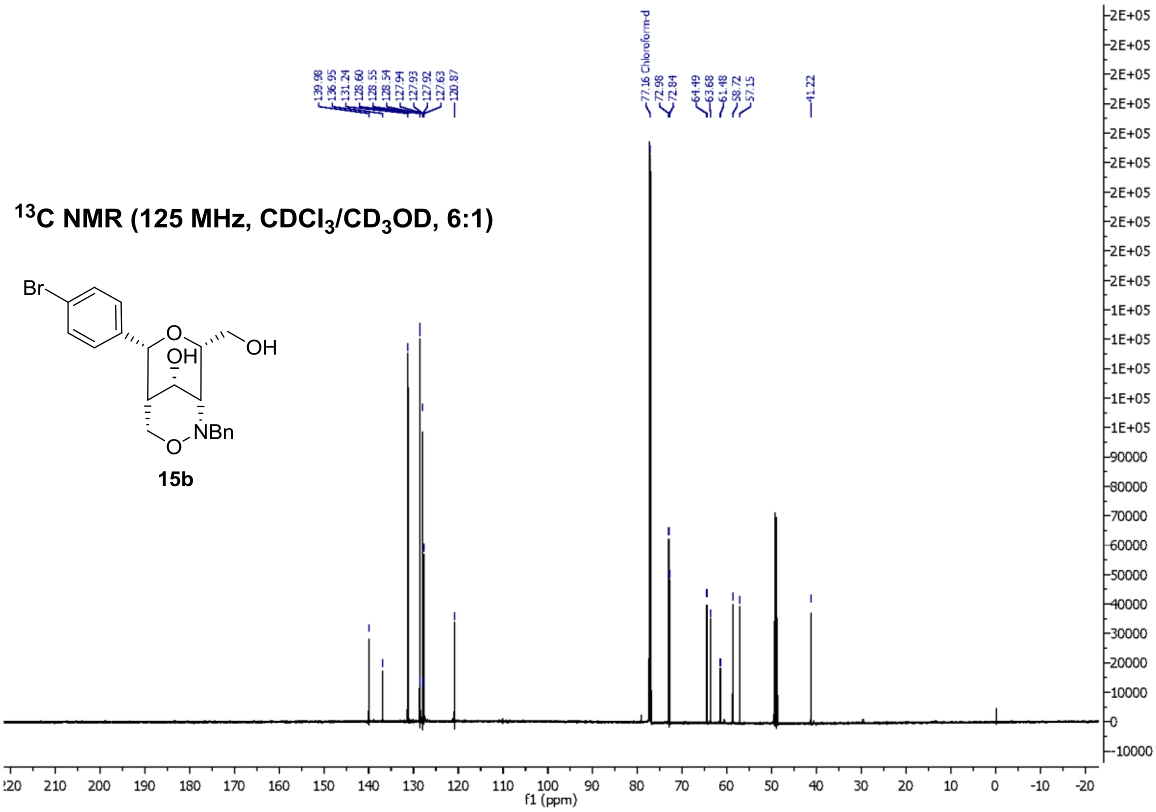
13C NMR (125 MHz, CDCl3/CD3OD, 6:1)
77.16 Chloroform-d
Br
O
OH
OH
NBn
O
15b
f1 (ppm)

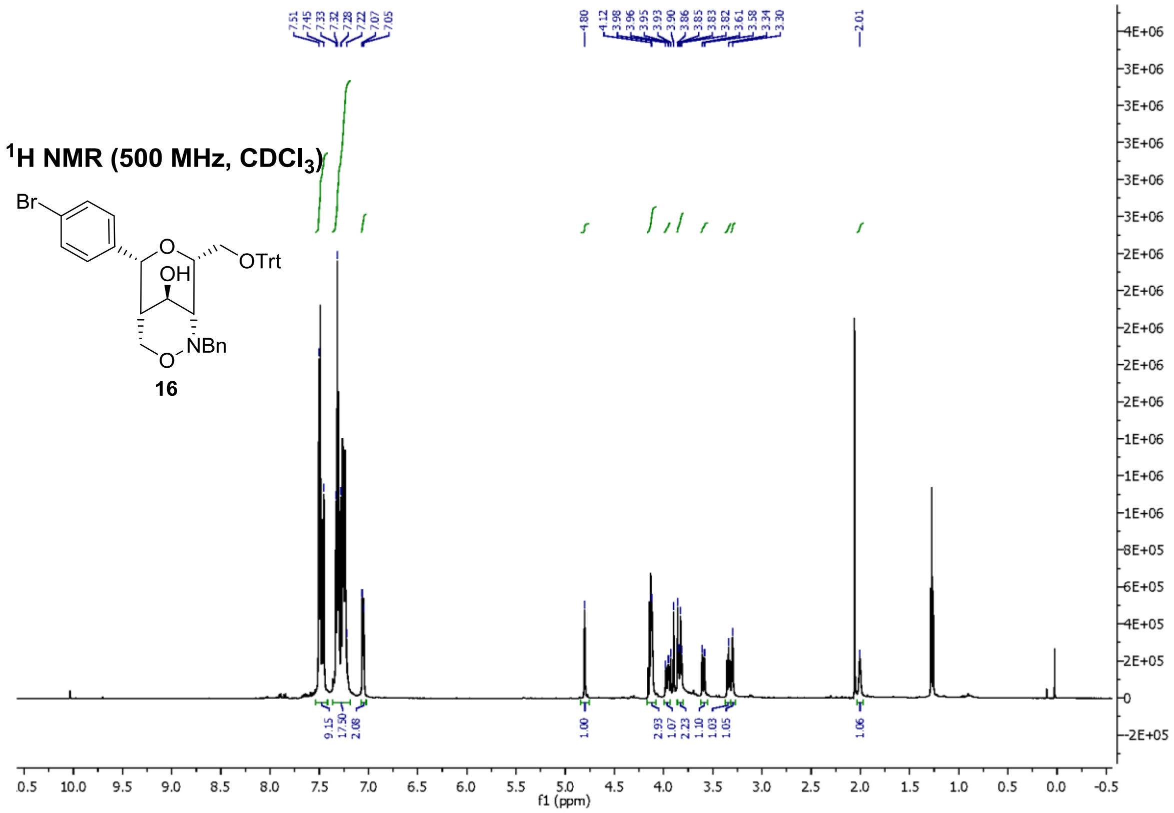

^{1}H NMR (500 MHz, $CDCl_3$)
Br
O
OH
OTrt
NBn
O
16
7.51 7.45 7.33 7.32 7.28 7.22 7.07 7.05
4.80 4.12 3.98 3.96 3.95 3.93 3.90 3.86 3.85 3.83 3.82 3.61 3.58 3.34 3.30
2.01
9.15 17.50 2.08 1.00 2.93 1.07 2.23 1.10 1.03 1.05 1.06
f1 (ppm)

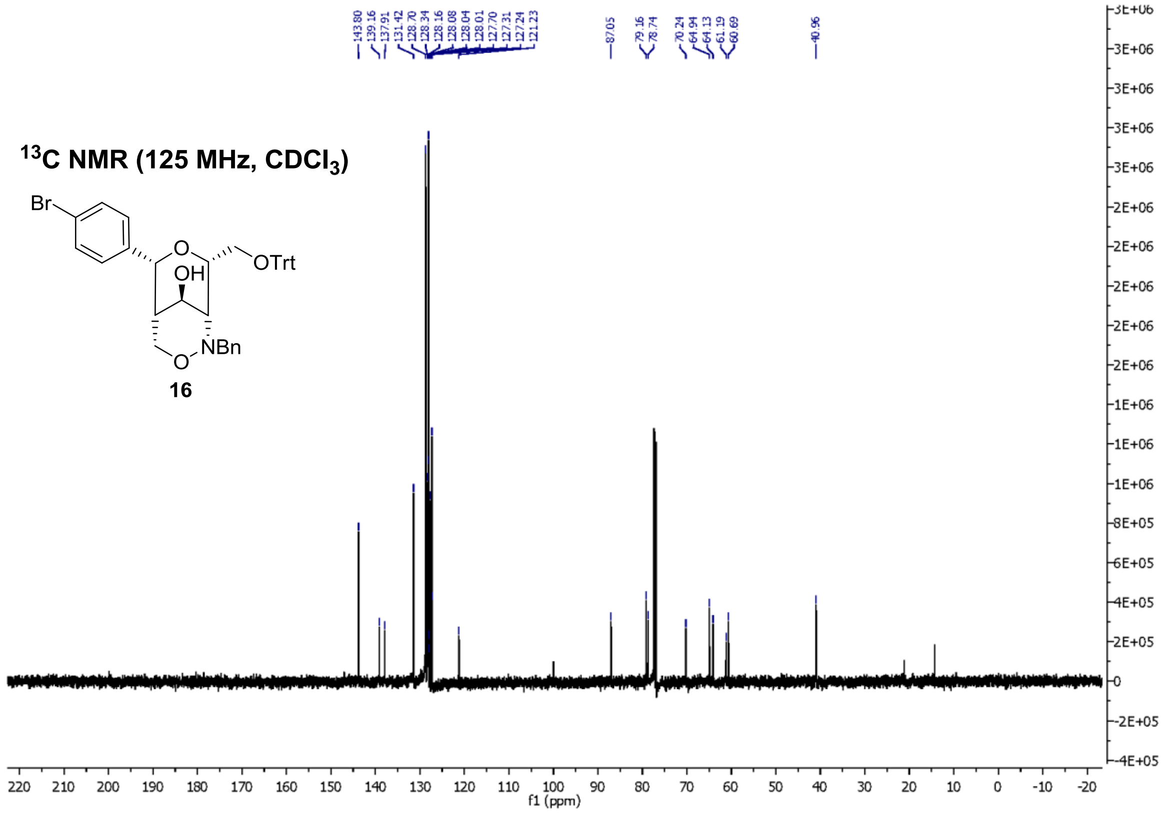

^{13}C NMR (125 MHz, $CDCl_3$)
Br
O
OH
OTrt
NBn
O
16
143.80 139.16 137.91 131.42 128.70 128.34 128.16 128.08 128.04 128.01 127.70 127.31 127.24 121.23
87.05 79.16 78.74 70.24 64.94 64.13 61.19 60.69
40.96
f1 (ppm)

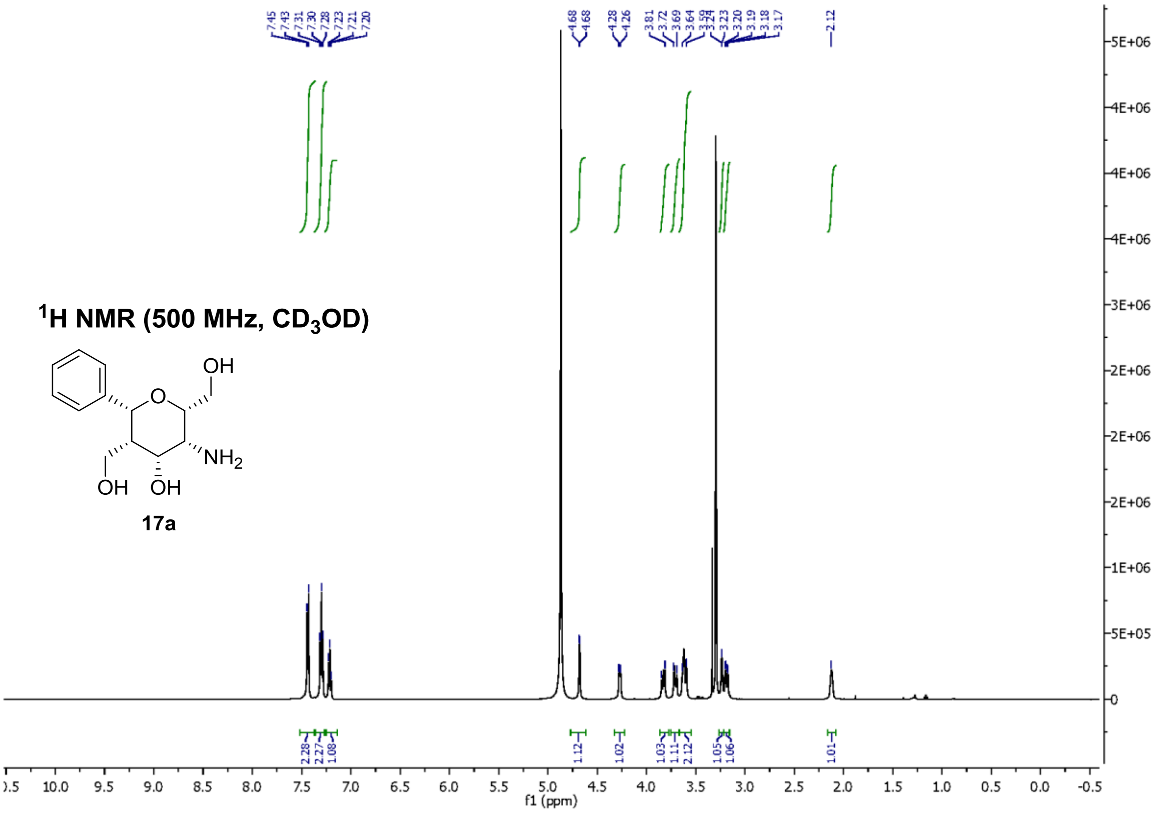
^{1}H NMR (500 MHz, CD_3OD)
17a
f1 (ppm)

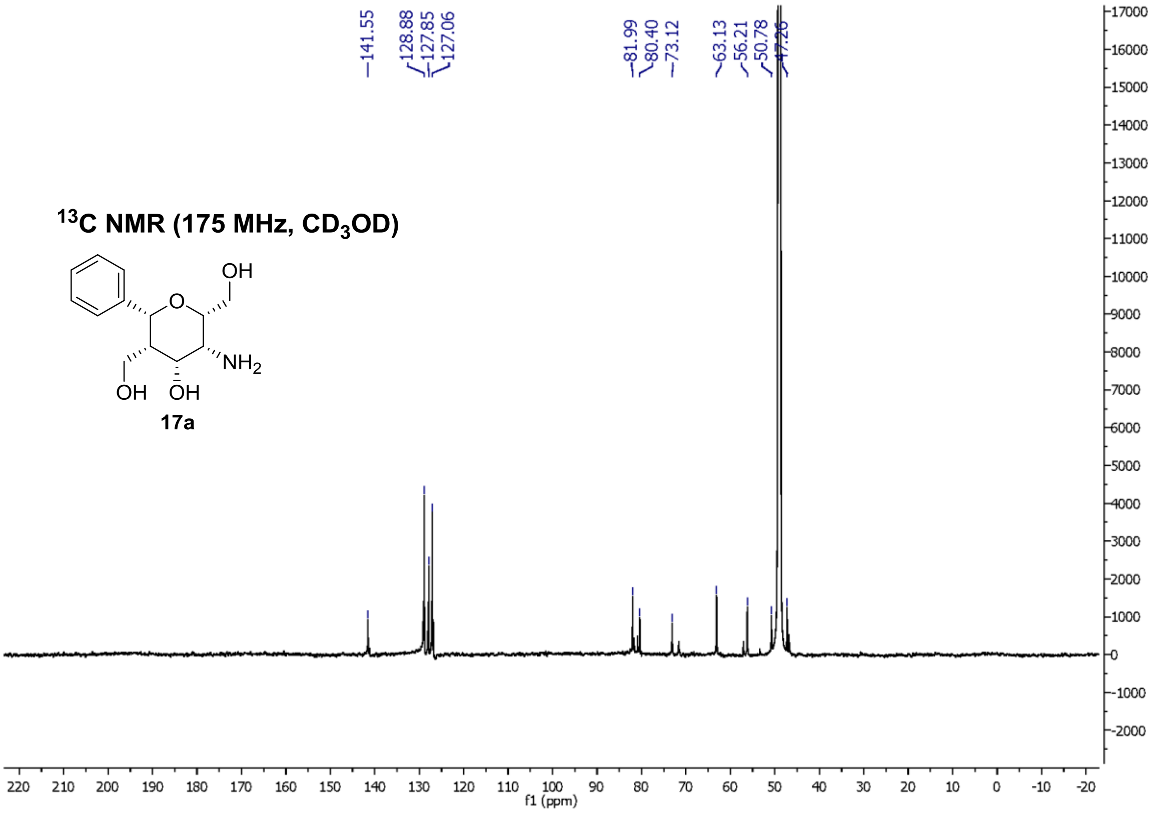
^{13}C NMR (175 MHz, CD_3OD)
17a
141.55
128.88
127.85
127.06
81.99
80.40
73.12
63.13
56.21
50.78
f1 (ppm)

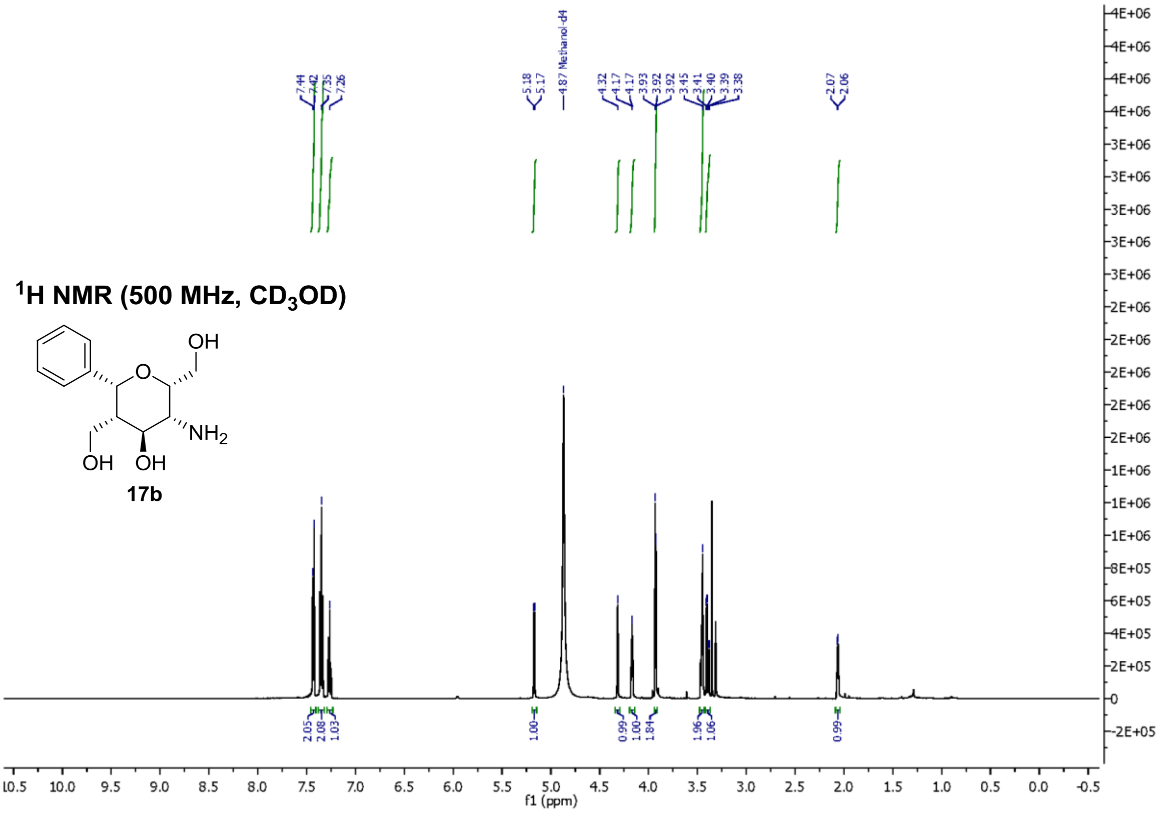

^{1}H NMR (500 MHz, CD_3OD)

17b

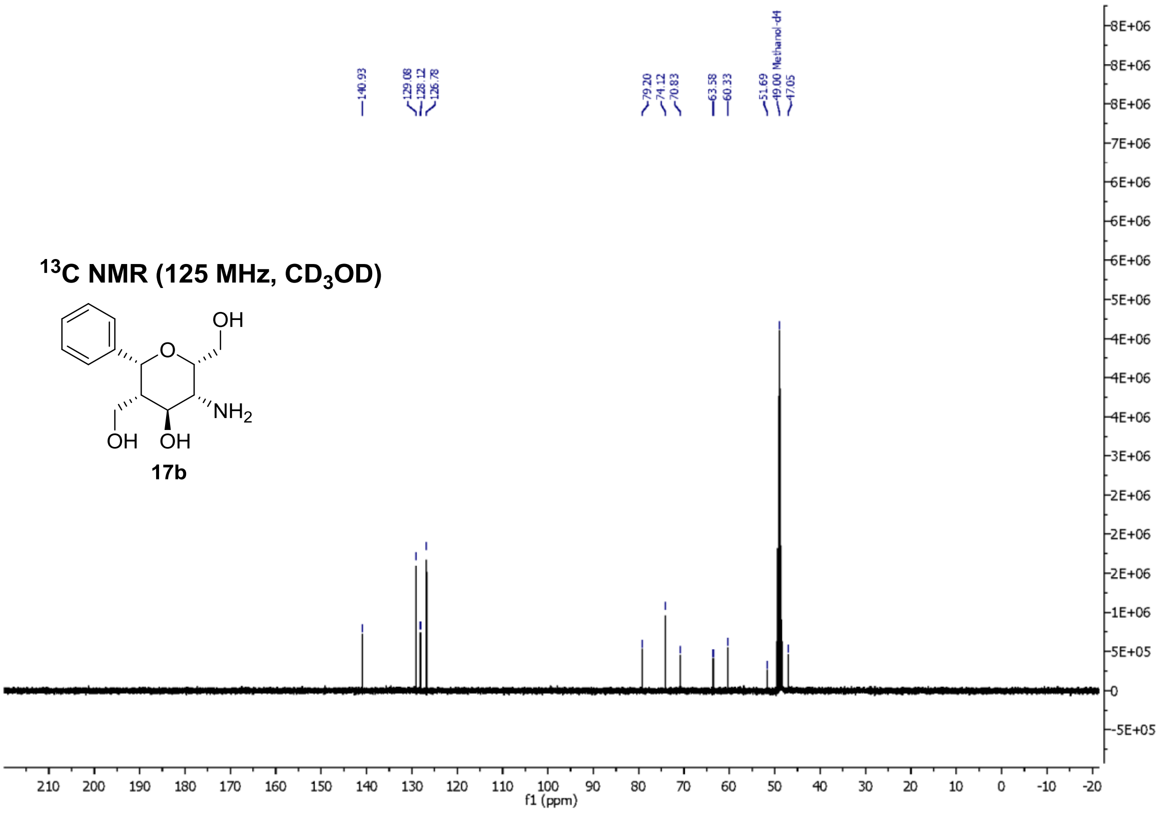

^{13}C NMR (125 MHz, CD_3OD)

17b

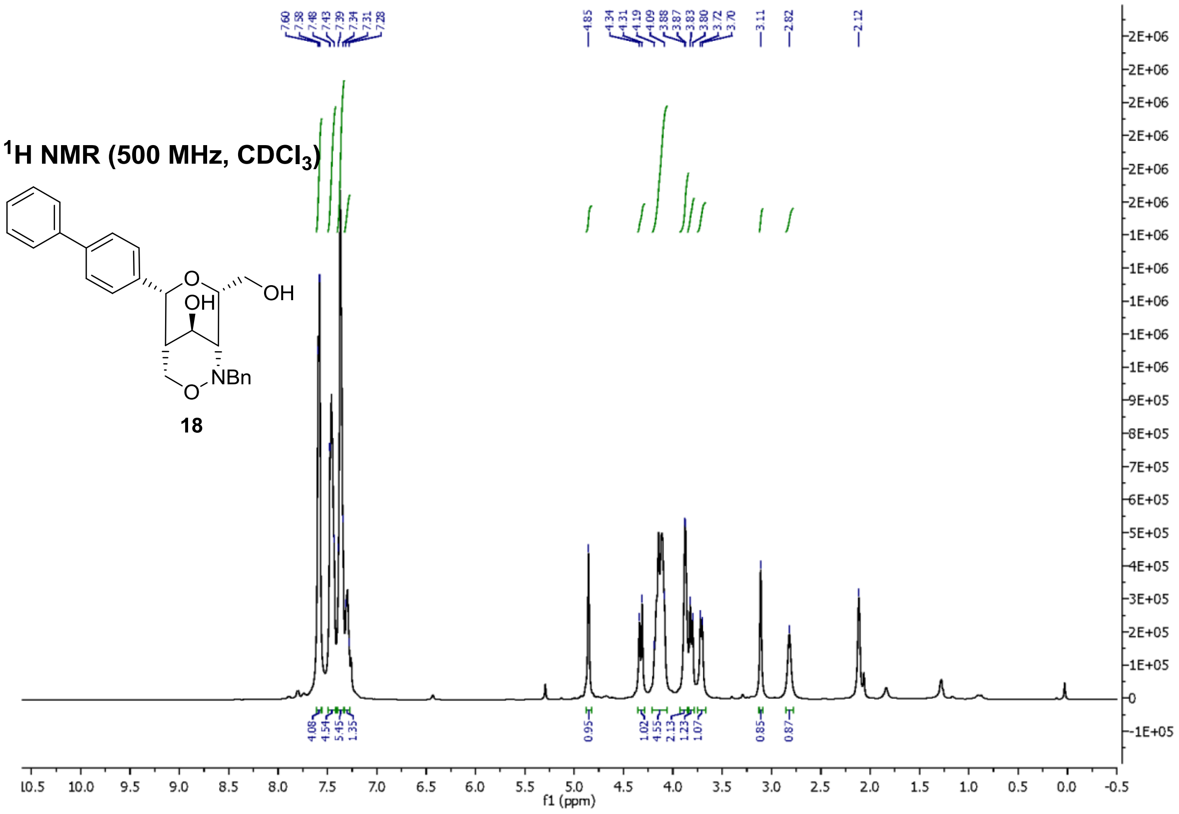
7.60 7.58 7.48 7.43 7.39 7.34 7.31 7.28
4.85 4.34 4.31 4.19 4.09 3.88 3.87 3.83 3.80 3.72 3.70
3.11
2.82
2.12
1H NMR (500 MHz, CDCl3)
O
OH
OH
NBn
O
18
f1 (ppm)

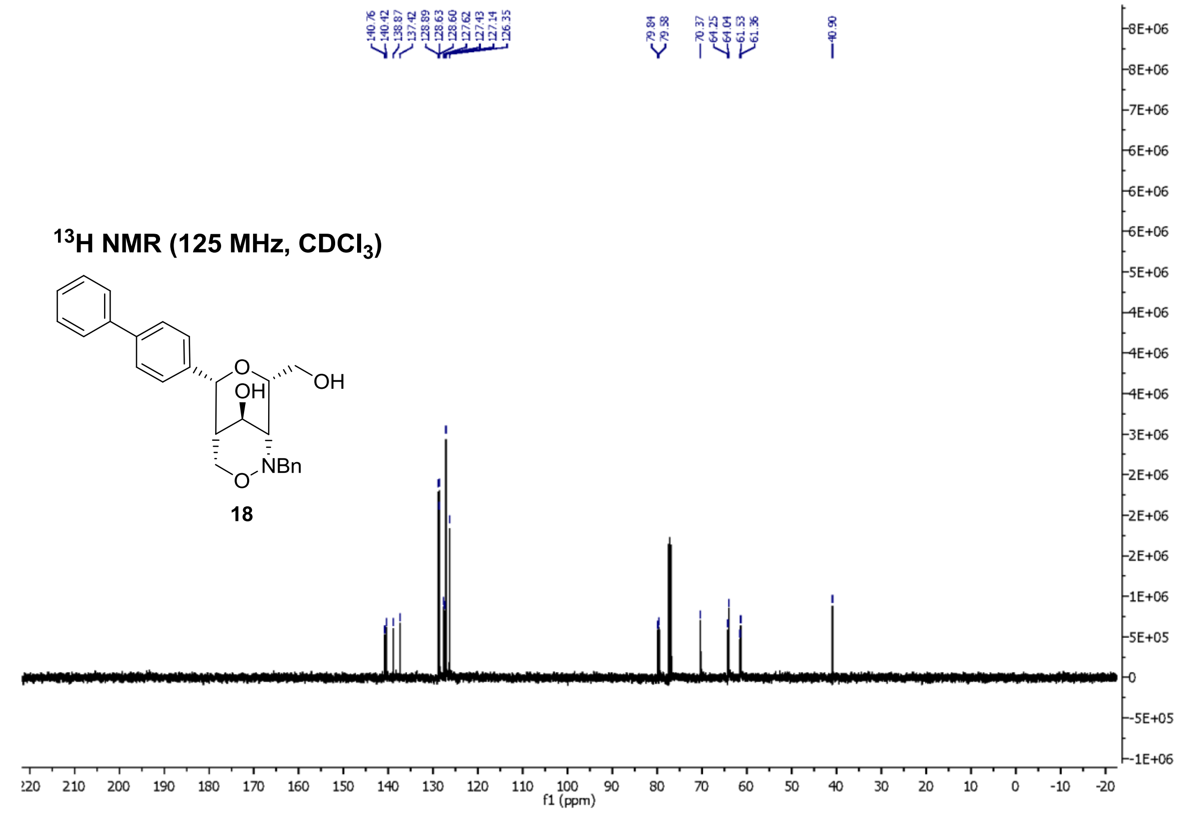
140.76 140.42 138.87 137.42 128.89 128.63 128.60 127.62 127.43 127.14 126.35
79.84 79.58
70.37 64.25 64.04 61.53 61.36
40.90
13H NMR (125 MHz, CDCl3)
O
OH
OH
NBn
O
18
f1 (ppm)

^{1}H NMR (700 MHz, $CD_3OD/CDCl_3$, 2:1)

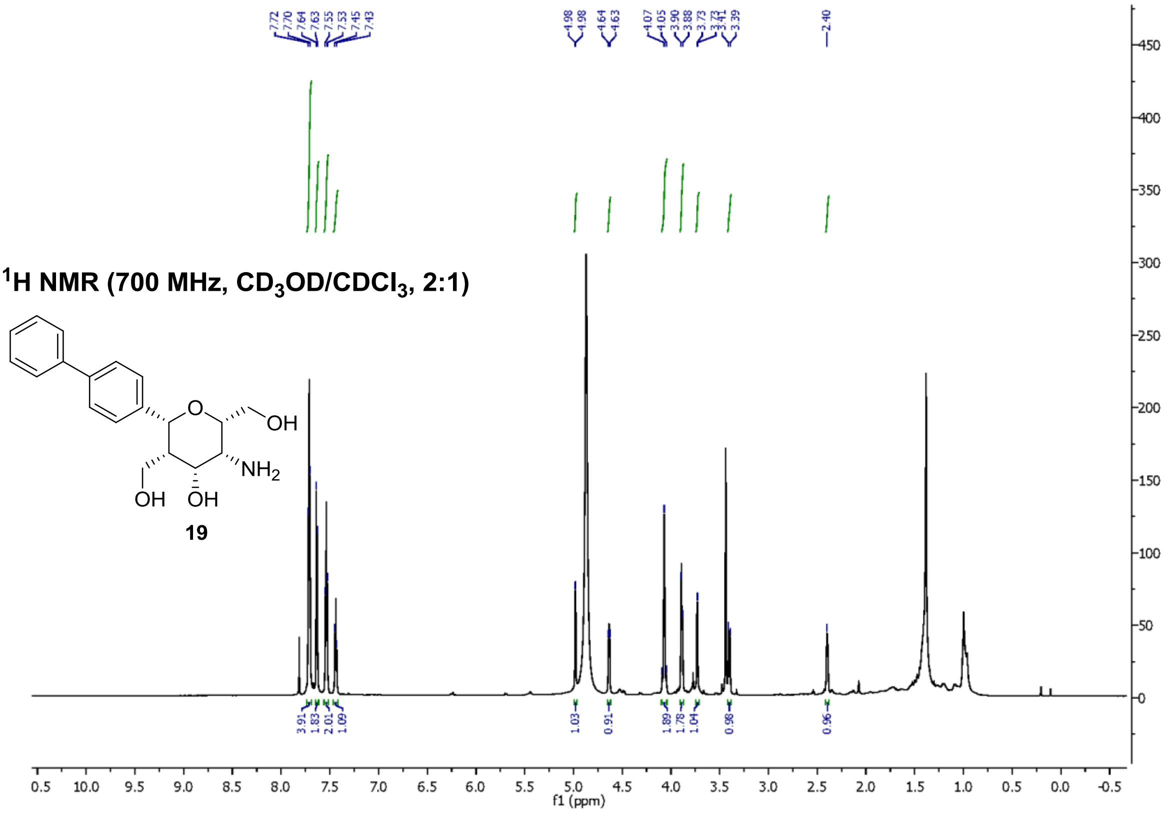

^{13}C NMR (175 MHz, $CD_3OD/CDCl_3$, 2:1)

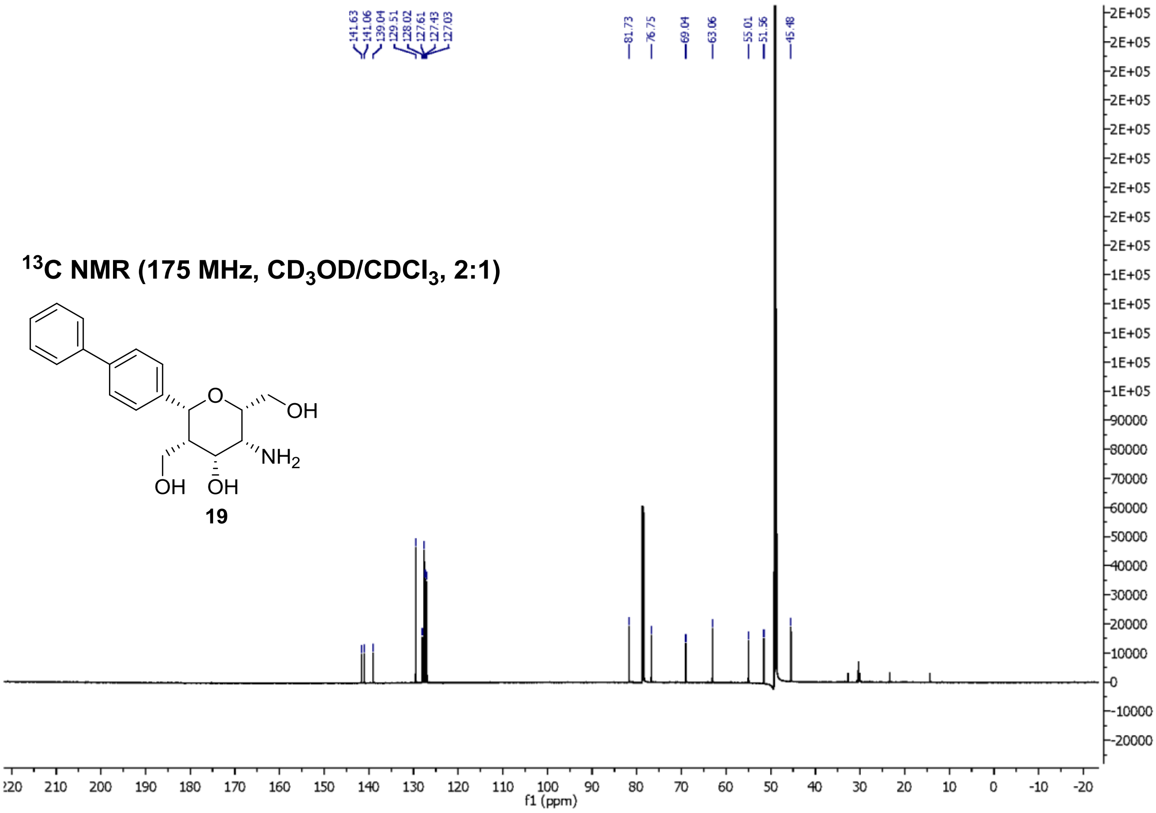

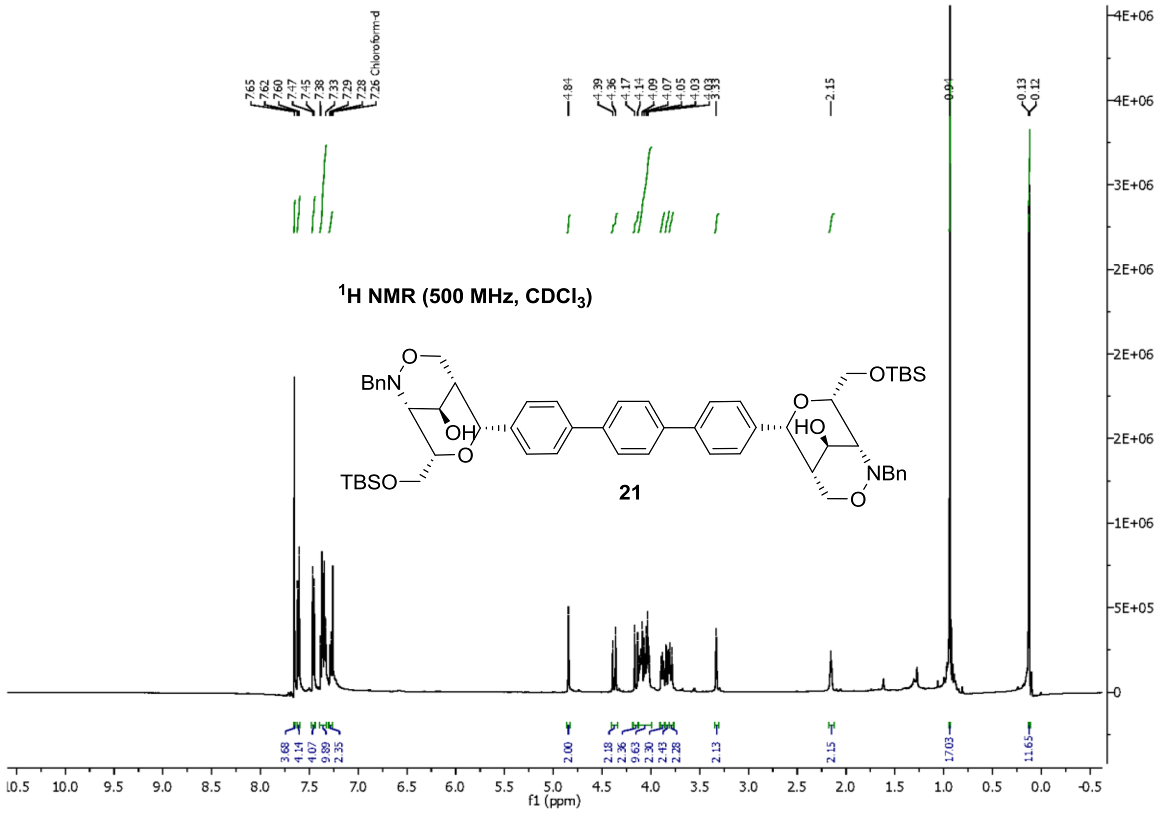
1H NMR (500 MHz, CDCl3)
BnN
OH
TBSO
OTBS
HO
NBn
21
Chloroform-d
f1 (ppm)

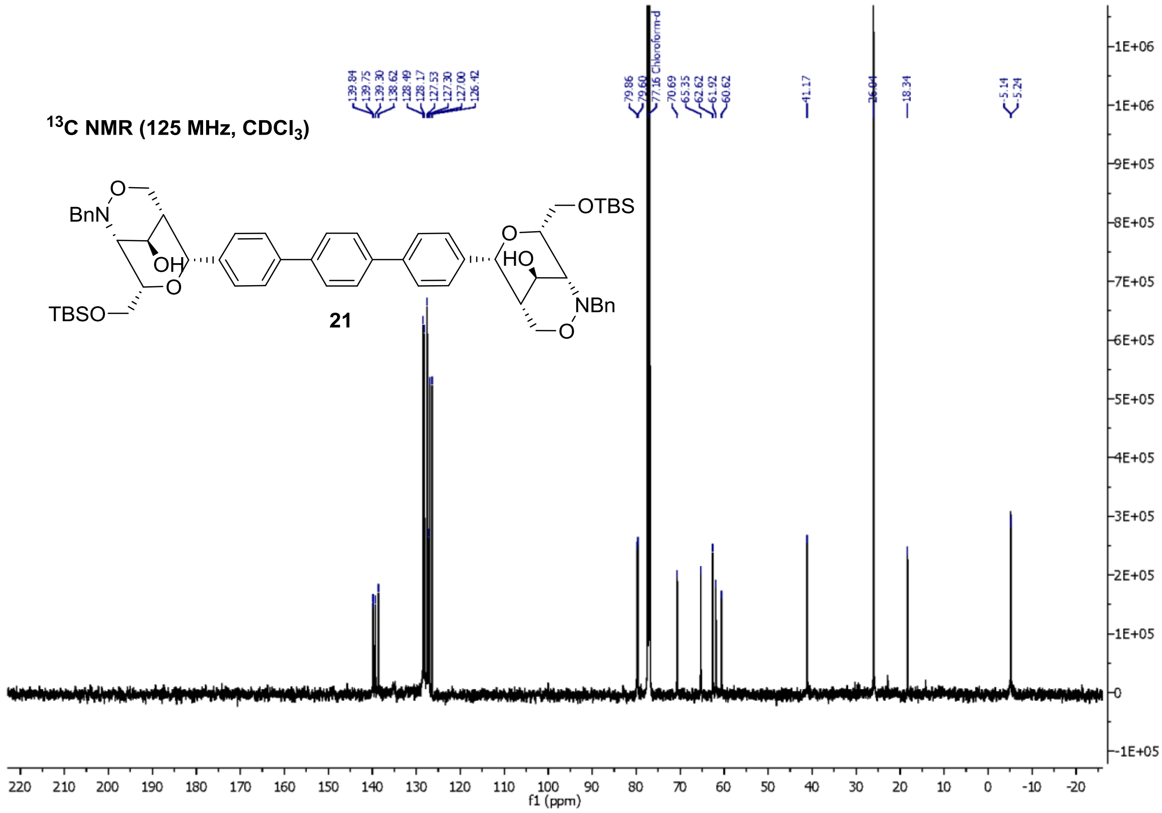
13C NMR (125 MHz, CDCl3)
BnN
OH
TBSO
OTBS
HO
NBn
21
Chloroform-d
f1 (ppm)

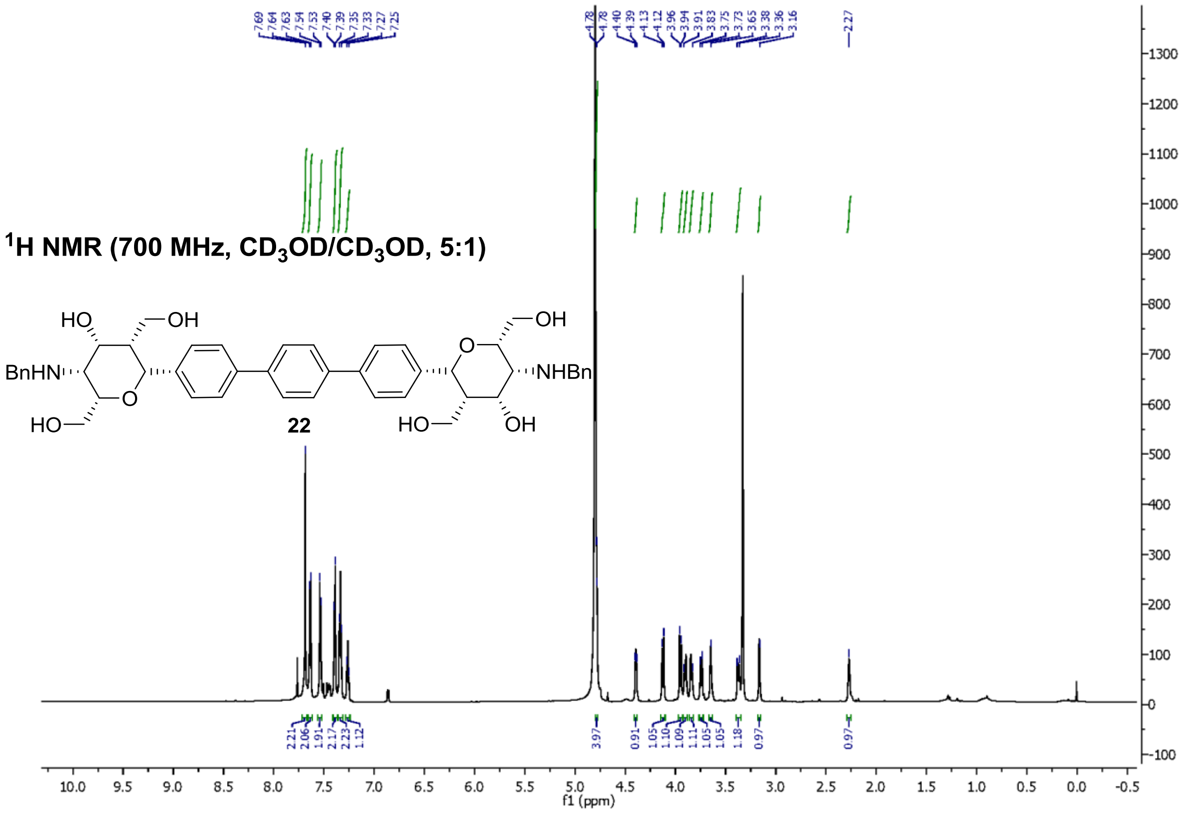

^{1}H NMR (700 MHz, CD$_3$OD/CD$_3$OD, 5:1)
HO
OH
BnHN
O
HO
22
OH
O
NHBn
HO
OH
f1 (ppm)

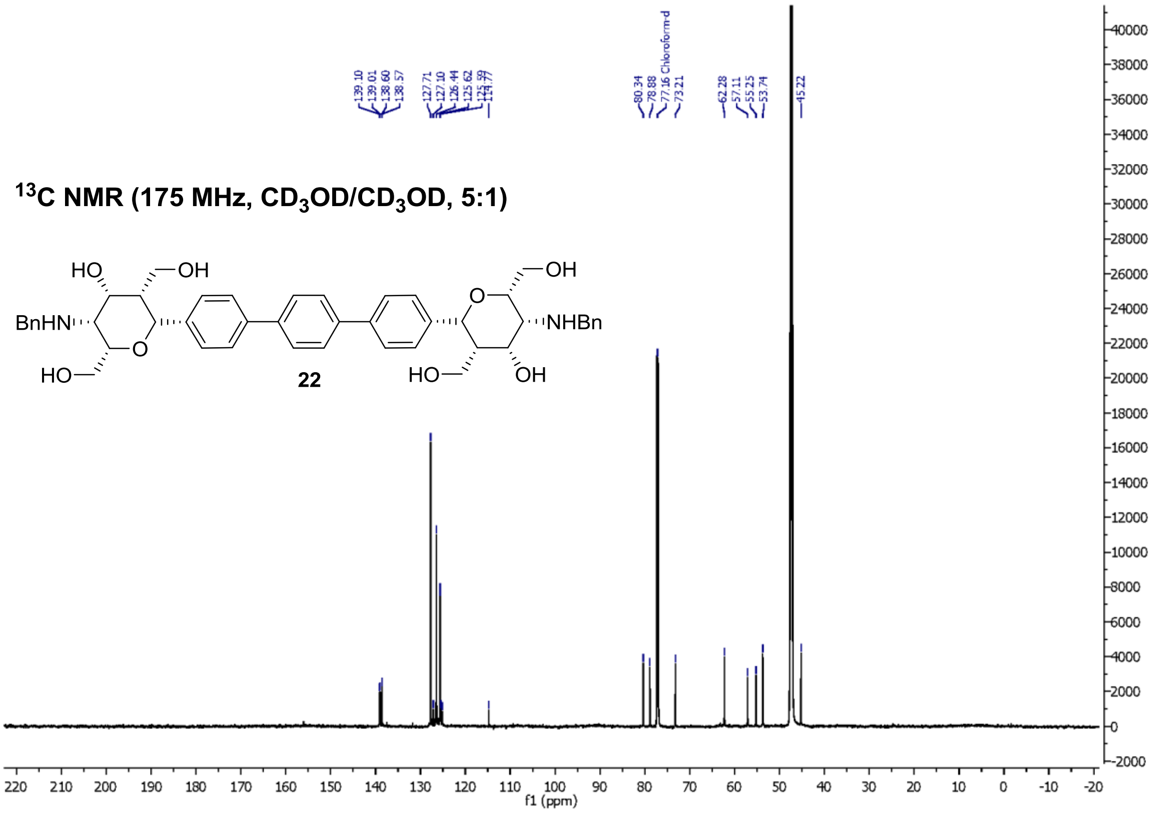

^{13}C NMR (175 MHz, CD$_3$OD/CD$_3$OD, 5:1)
HO
OH
BnHN
O
HO
22
OH
O
NHBn
HO
OH
f1 (ppm)

^{1}H NMR (500 MHz, $CDCl_3$)

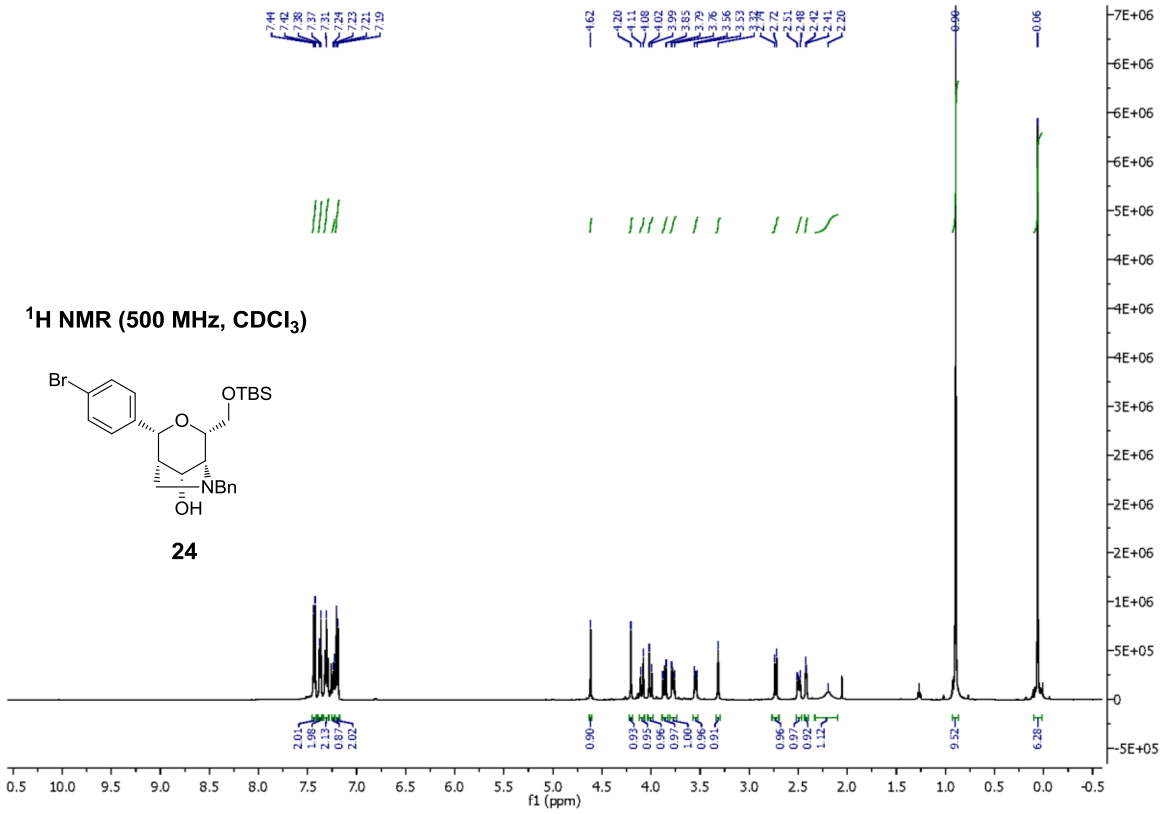

24

^{13}C NMR (125 MHz, $CDCl_3$)

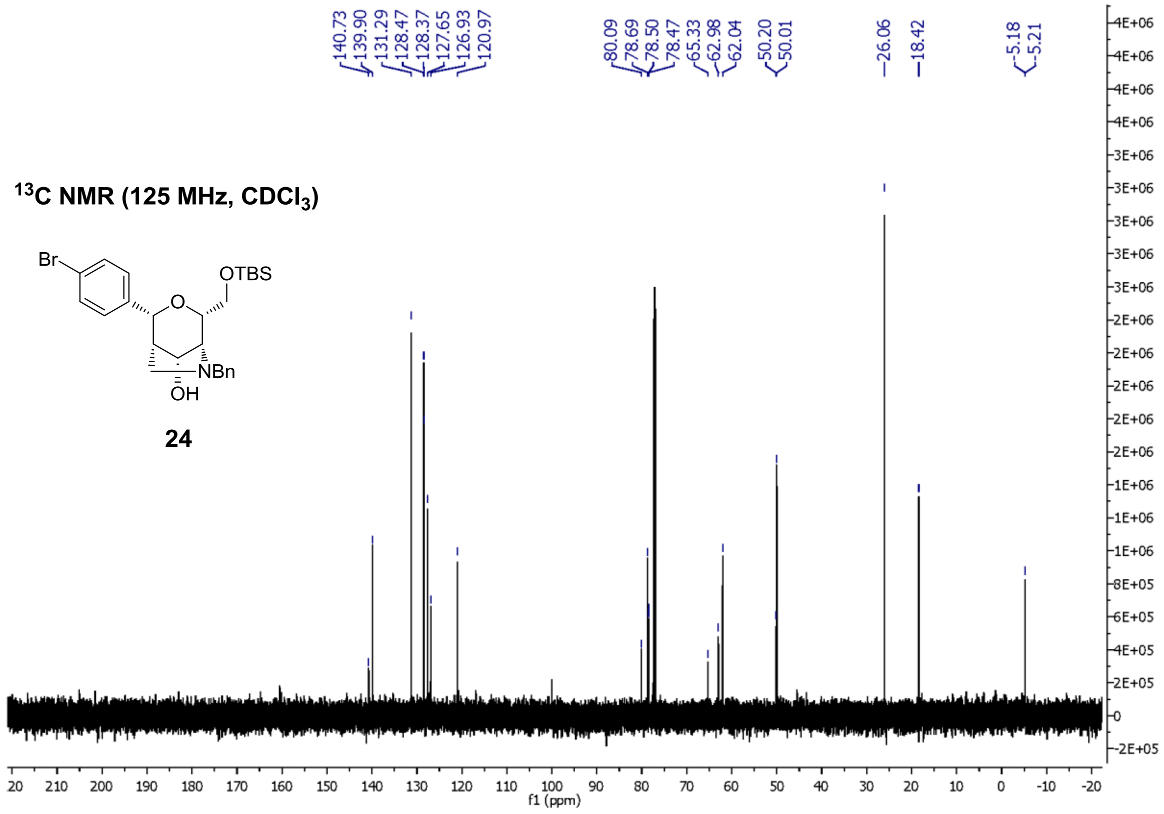

24

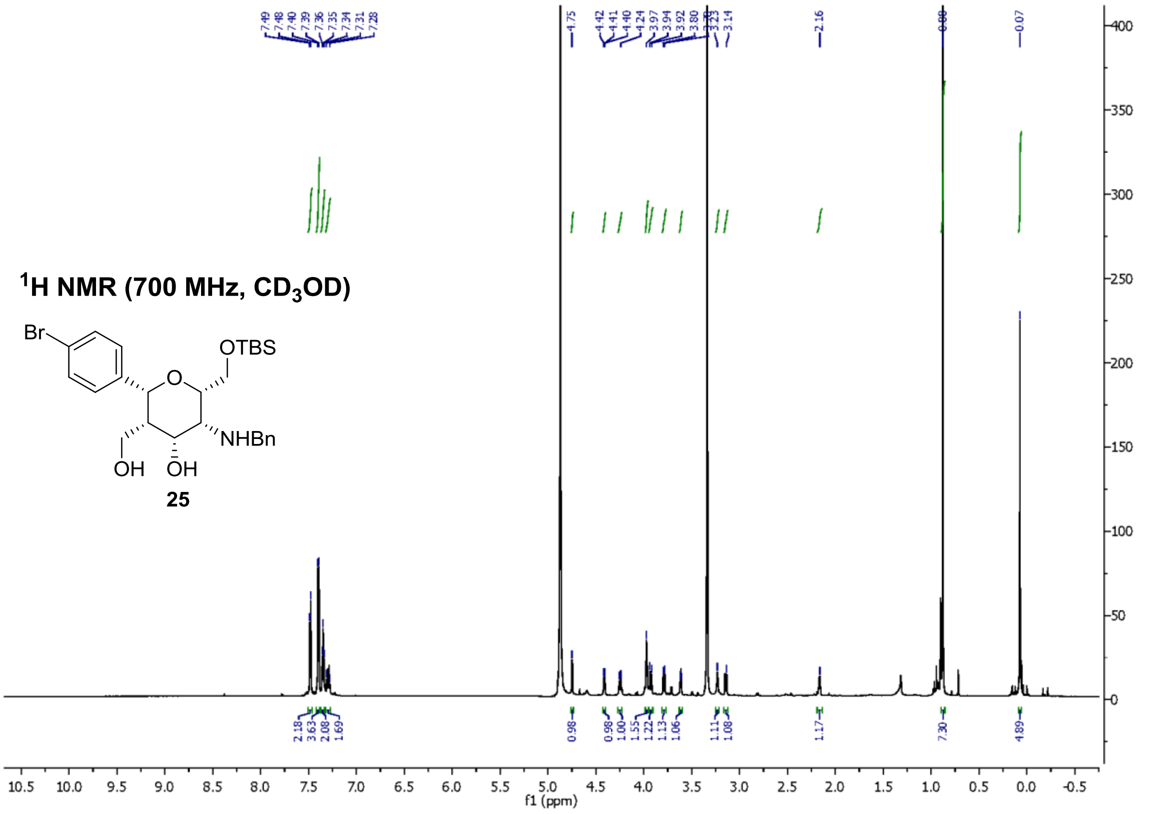
1H NMR (700 MHz, CD3OD)
Br
OTBS
O
NHBn
OH OH
25
7.49 7.48 7.40 7.39 7.36 7.35 7.34 7.31 7.28
4.75
4.42 4.41 4.40 4.24 3.97 3.94 3.92 3.80
3.23 3.14
2.16
0.88
0.07
f1 (ppm)

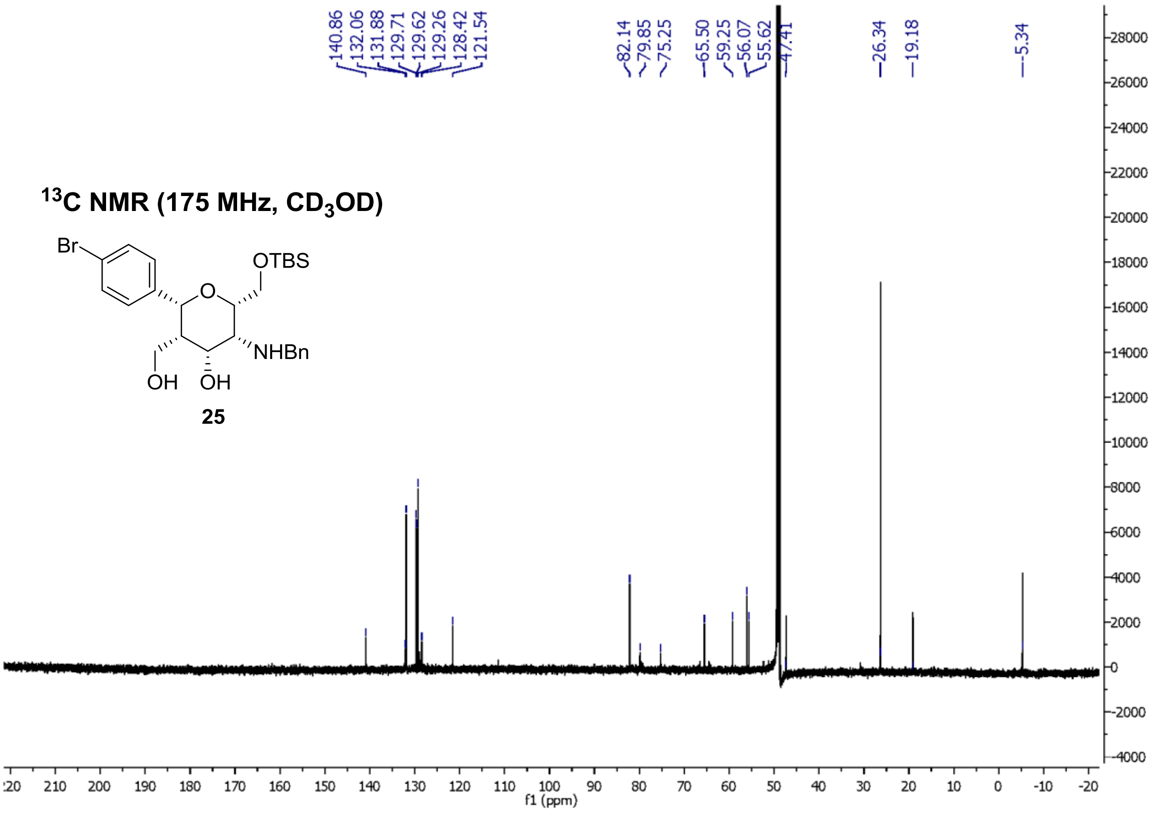
13C NMR (175 MHz, CD3OD)
Br
OTBS
O
NHBn
OH OH
25
140.86 132.06 131.88 129.71 129.62 129.26 128.42 121.54
82.14 79.85 75.25
65.50 59.25 56.07 55.62
47.41
26.34 19.18
-5.34
f1 (ppm)

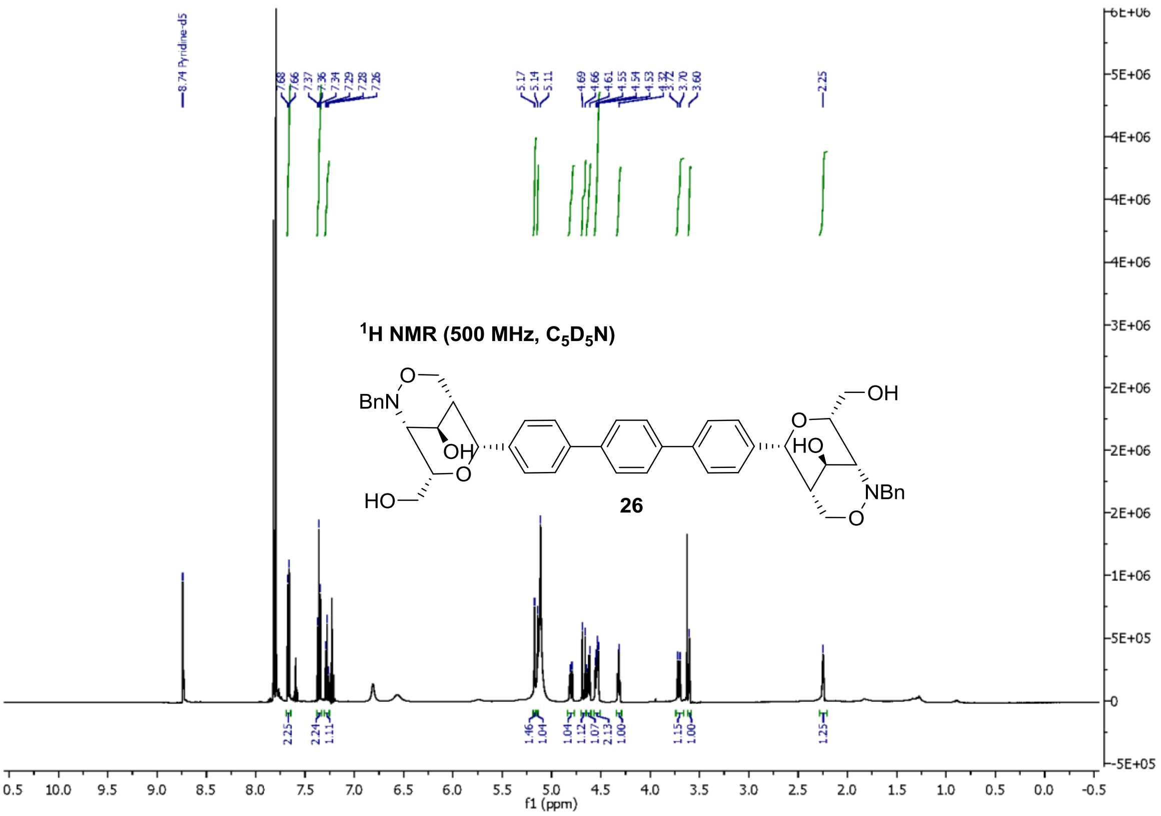

1H NMR (500 MHz, C5D5N)
8.74 Pyridine-d5
7.68
7.66
7.37
7.36
7.34
7.29
7.28
7.26
5.17
5.14
5.11
4.69
4.66
4.61
4.55
4.54
4.53
4.32
3.72
3.70
3.60
2.25
BnN
O
OH
HO
NBn
26
f1 (ppm)

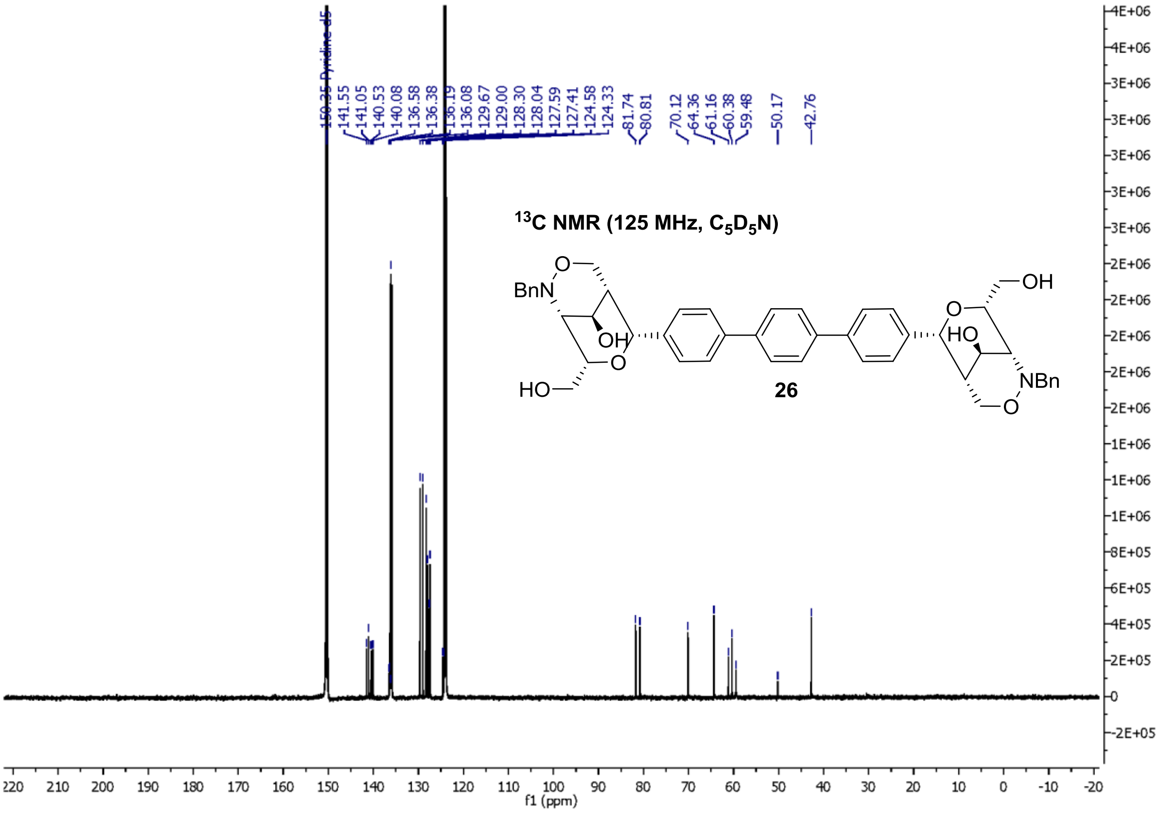

13C NMR (125 MHz, C5D5N)
150.35 Pyridine-d5
141.55
141.05
140.53
140.08
136.58
136.38
136.19
136.08
129.67
129.00
128.30
128.04
127.59
127.41
124.58
124.33
81.74
80.81
70.12
64.36
61.16
60.38
59.48
50.17
42.76
BnN
O
OH
HO
NBn
26
f1 (ppm)

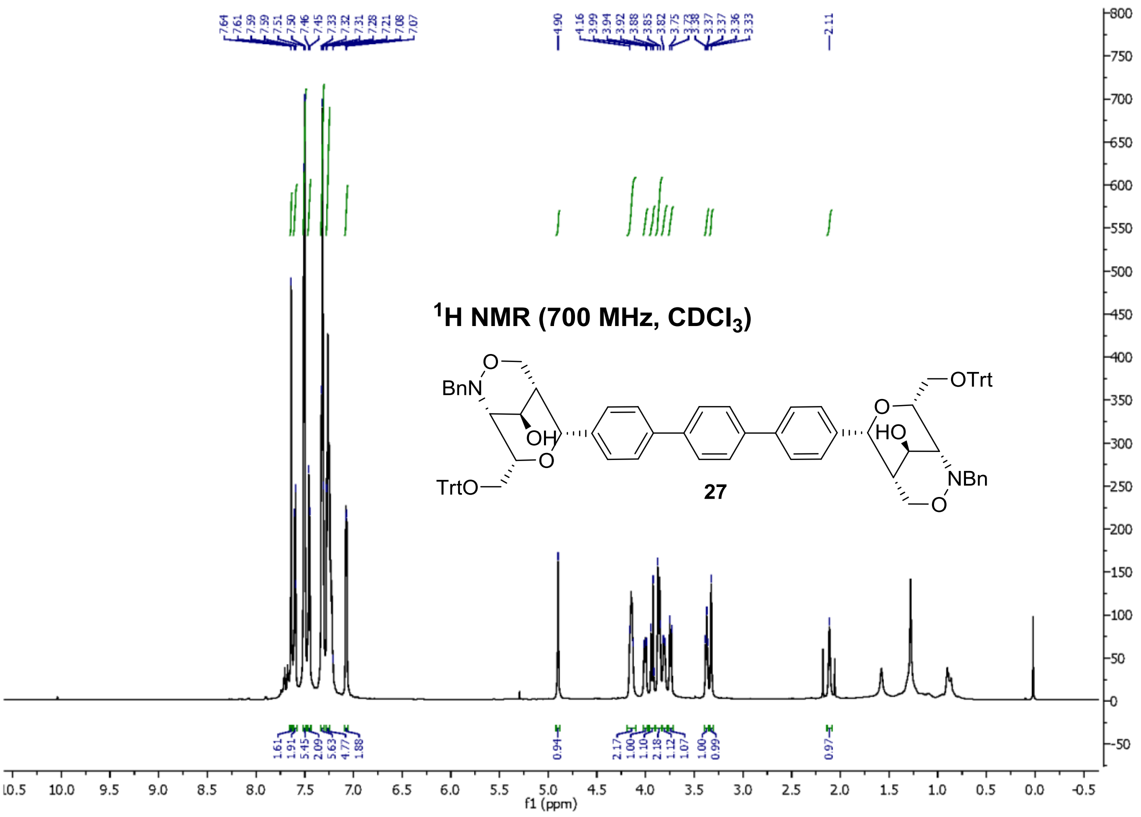

1H NMR (700 MHz, CDCl3)
BnN
OH
TrtO
OTrt
HO
NBn
27
f1 (ppm)

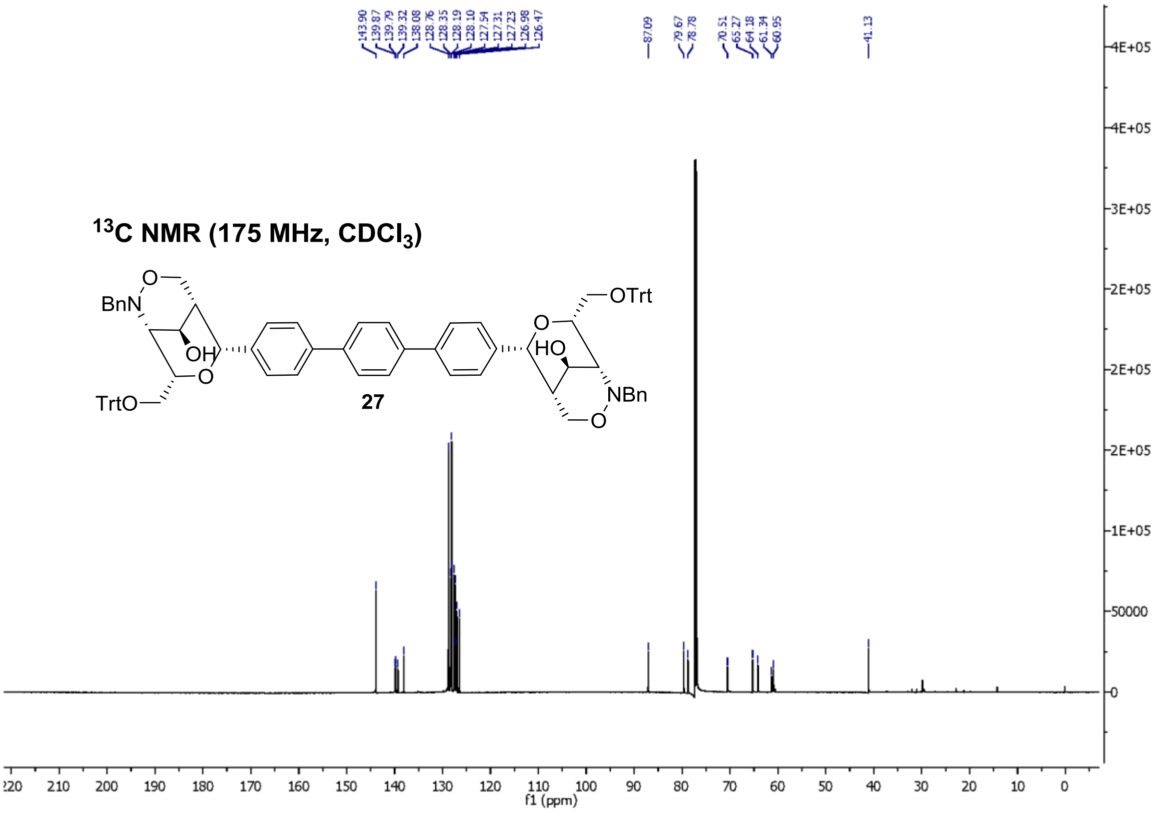

13C NMR (175 MHz, CDCl3)
BnN
OH
TrtO
OTrt
HO
NBn
27
f1 (ppm)

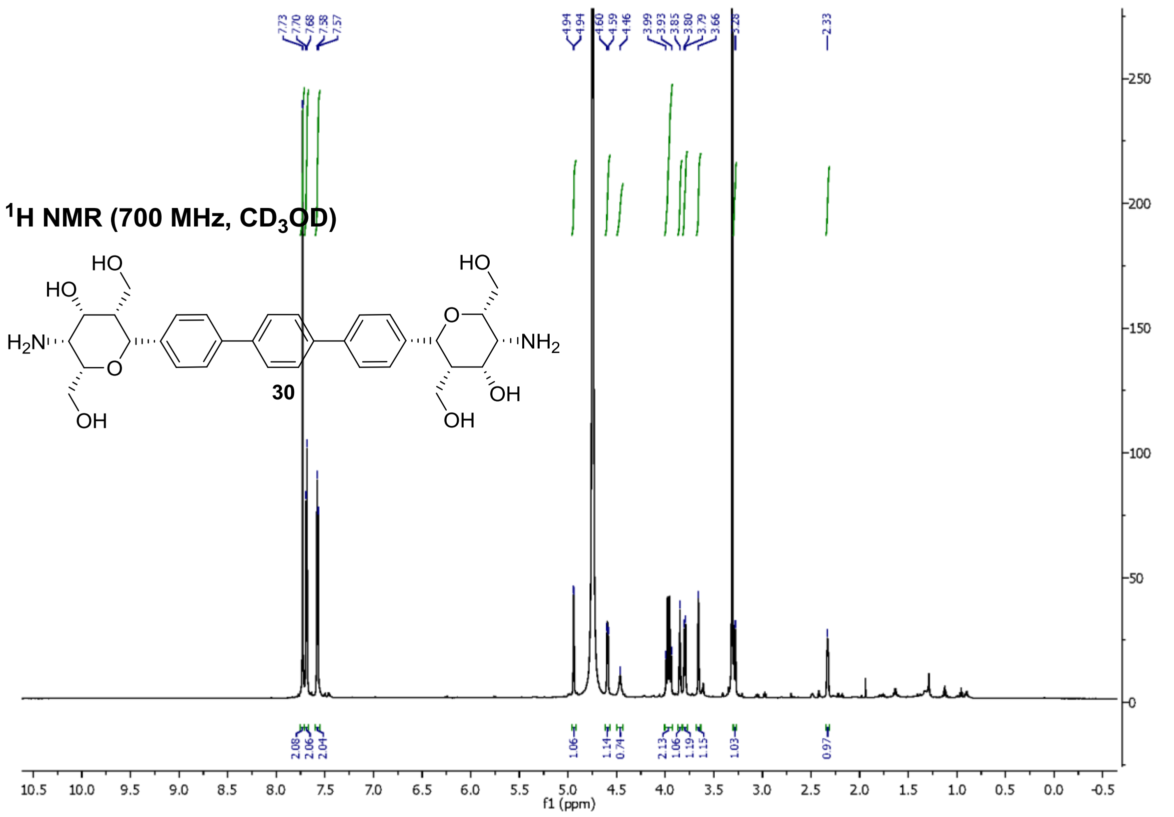

1H NMR (700 MHz, CD3OD)
30
HO
HO
H2N
O
OH
NH2
7.73 7.70 7.68 7.58 7.57
4.94 4.94 4.60 4.59 4.46
3.99 3.93 3.85 3.80 3.79 3.66 3.28
2.33
f1 (ppm)

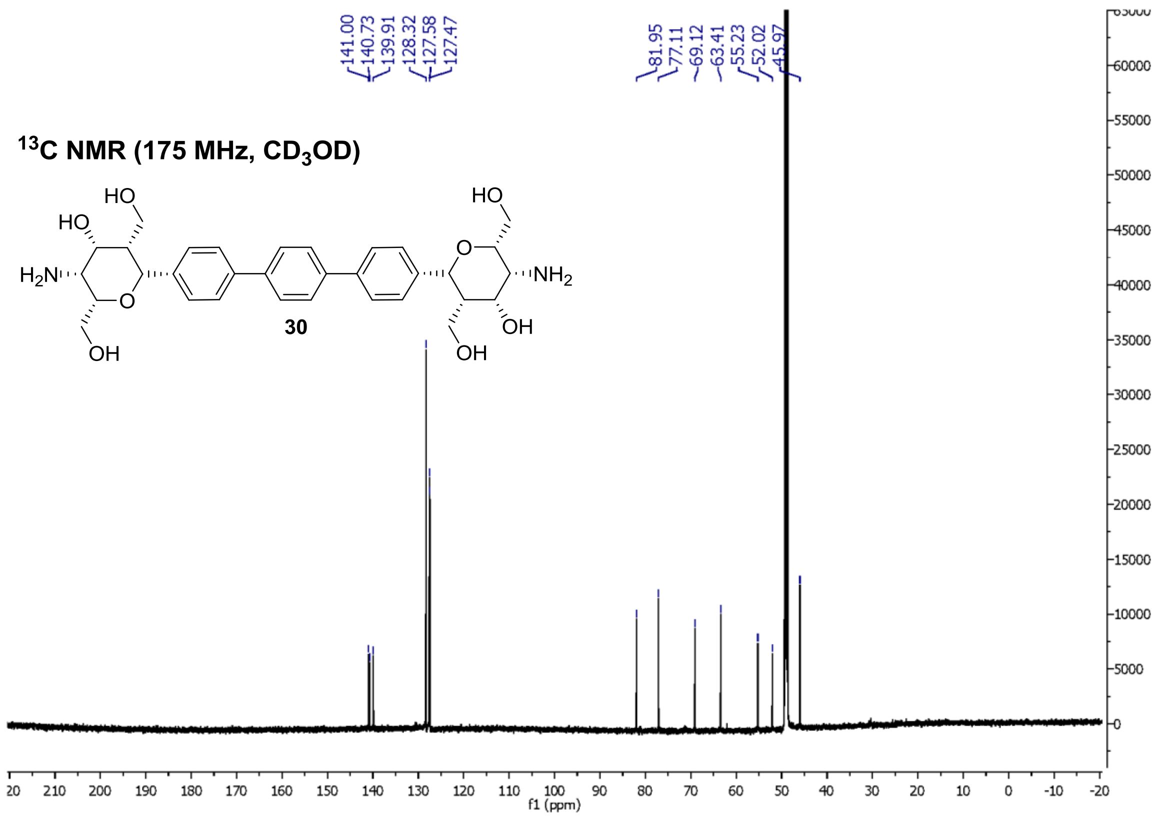

13C NMR (175 MHz, CD3OD)
30
HO
HO
H2N
O
OH
NH2
141.00 140.73 139.91 128.32 127.58 127.47
81.95 77.11 69.12 63.41 55.23 52.02 45.97
f1 (ppm)

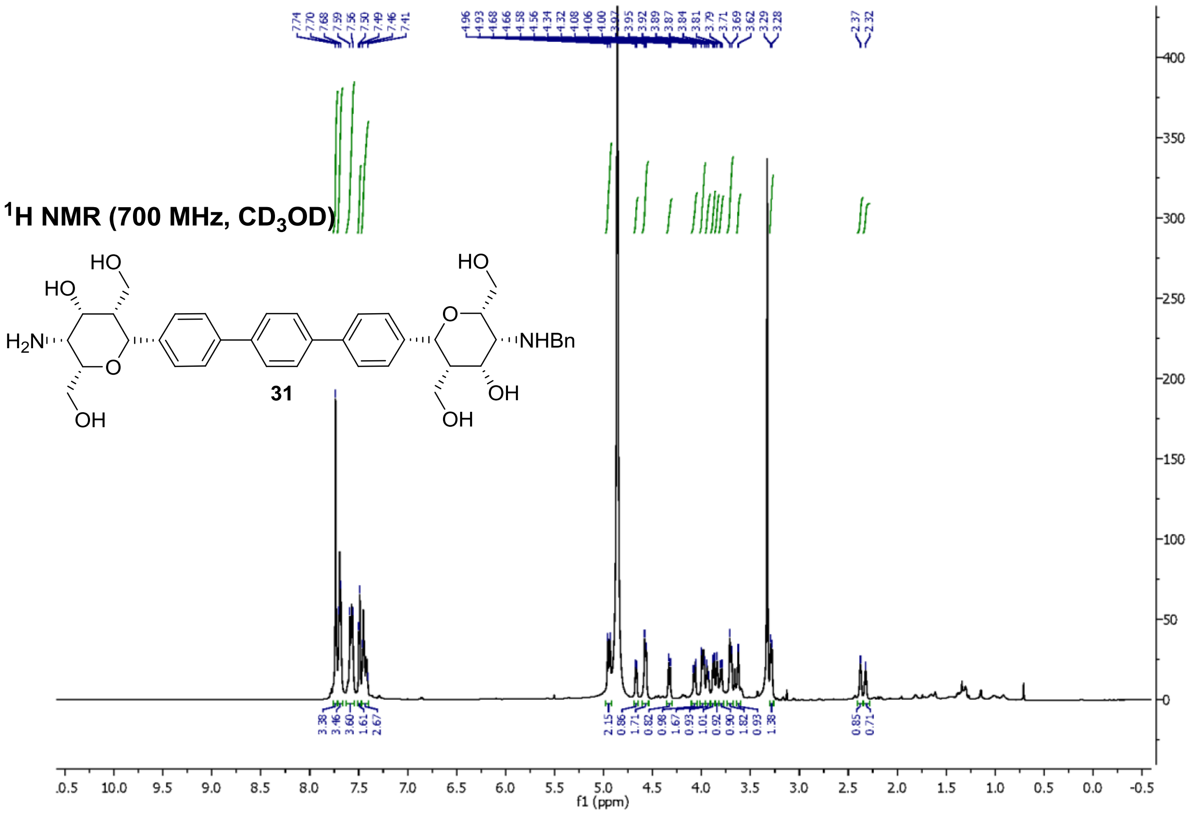
^{1}H NMR (700 MHz, CD_3OD)
HO
HO
H_2N
O
OH
31
NHBn
OH
OH
f1 (ppm)

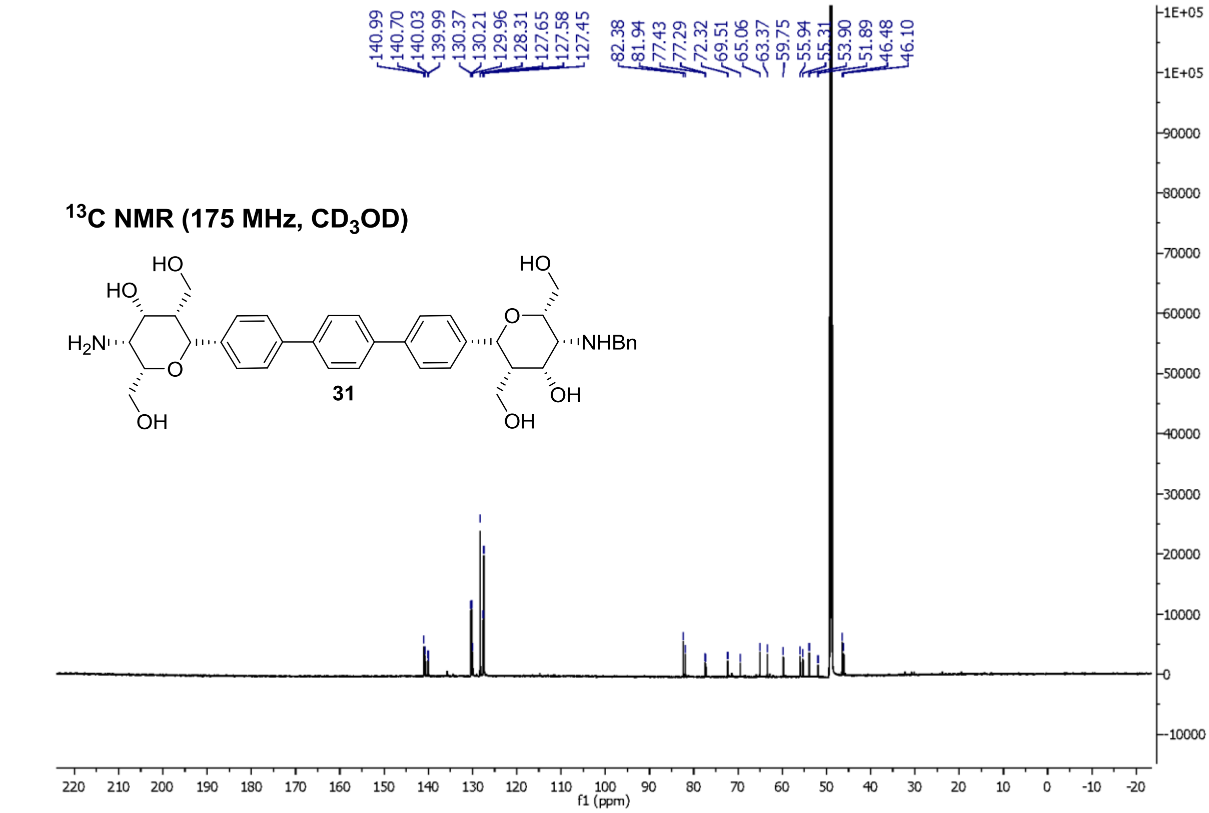
^{13}C NMR (175 MHz, CD_3OD)
140.99
140.70
140.03
139.99
130.37
130.21
129.96
128.31
127.65
127.58
127.45
82.38
81.94
77.43
77.29
72.32
69.51
65.06
63.37
59.75
55.94
55.31
53.90
51.89
46.48
46.10
HO
HO
H_2N
O
OH
31
NHBn
OH
OH
f1 (ppm)
